# TRIM62 facilitates influenza A virus entry by regulating WASH-dependent endosomal trafficking

**DOI:** 10.1038/s44319-026-00942-x

**Published:** 2026-09-19

**Authors:** Kajal Gupta, Sampurna Pal, Arpit Tyagi, Roohani Bajaj, Tejal Pathak, Gaganpreet Kaur, Deepak Sharma, Indranil Banerjee

**Affiliations:** 1 Department of Biological Sciences, Indian Institute of Science Education and Research (IISER) Mohali, Mohali, 140306 India; 2 Protein Science and Engineering, Council of Scientific and Industrial Research-Institute of Microbial Technology, Chandigarh, India; 3 Academy of Scientific and Innovative Research (AcSIR), Ghaziabad, India

**Keywords:** Membranes & Trafficking, Microbiology, Virology & Host Pathogen Interaction, Post-translational Modifications & Proteolysis

## Abstract

Endocytosis enables efficient cargo uptake while restricting uncontrolled cellular entry, yet how this balance is enforced during endocytic uptake remains poorly understood. Using influenza A virus (IAV) as a model cargo, we reveal a previously unrecognized role for the pentameric WASH complex (WASH) as a regulatory hub that integrates both permissive and inhibitory functions to control viral endocytosis. While the WASH subunits WASH1, CCDC53, SWIP, and Strumpellin collectively promote IAV entry, the FAM21 subunit acts antagonistically. Our data suggest that FAM21-mediated association of WASH with the retromer subunit VPS35 restrains the pro-endocytic activity of WASH, thereby limiting viral uptake. This inhibitory FAM21-VPS35 axis is counteracted by the E3 ubiquitin ligase TRIM62, which maintains WASH in a retromer-free, endocytically competent state that facilitates viral internalization. Beyond IAV, the WASH1 subunit and VPS35 also exhibit opposing roles during cholera toxin B uptake, suggesting a broader function in lipid raft-mediated endocytosis. Together, our findings establish WASH as a key determinant of IAV endocytosis, with TRIM62 modulating retromer-mediated restriction to facilitate productive viral entry.

## Synopsis

Beyond its canonical role in branched actin nucleation at endosomes, WASH facilitates influenza A virus endocytosis. Retromer sequesters WASH at endosomes keeping it in a non-permissive state. TRIM62 limits this association, facilitating WASH-driven IAV entry.

- Distinct subunits of the pentameric WASH complex exert opposing effects on viral entry: WASH1, CCDC53, SWIP, and Strumpellin promote, whereas FAM21 antagonizes viral entry.
- FAM21 keeps WASH sequestered on endosomes via its interaction with the retromer subunit VPS35, preventing WASH-dependent viral internalization.
- TRIM62 binds WASH via FAM21, limits WASH-retromer association, thereby releasing WASH to facilitate viral entry.

## Abstract (web summary)

Beyond its canonical role in branched actin nucleation at endosomes, WASH facilitates influenza A virus endocytosis. Retromer sequesters WASH at endosomes keeping it in a non-permissive state. TRIM62 limits this association, facilitating WASH-driven IAV entry.

## Graphical abstract

**Figure:**
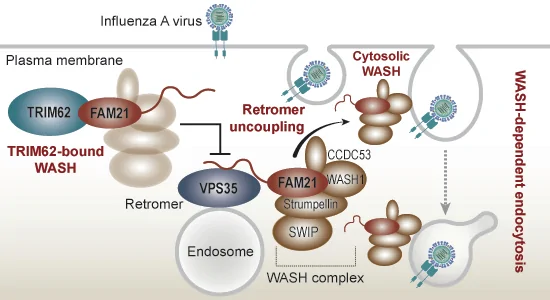

## Introduction

Endocytosis is an essential cellular process that drives the internalization of a wide variety of cargoes including extracellular proteins, receptor-ligand complexes, fluid, and plasma membrane lipids. It orchestrates membrane turnover and plays crucial role in intracellular signaling, neurotransmission, immune response, intercellular communications, thereby preserving cellular and organismal homeostasis (Conner and Schmid, 2003; Renard and Boucrot, 2021). At the plasma membrane of eukaryotic cells, multiple endocytic pathways operate concurrently to mediate cargo uptake. The canonical clathrin-mediated endocytosis (CME) supports critical house-keeping functions through the concentrative uptake of receptor-bound cargoes. In parallel to CME, clathrin-independent endocytosis operates to internalize selected cargoes and supports cellular needs that CME alone cannot meet (Renard and Boucrot, 2021; Johannes et al, 2015). Host endocytic pathways also afford pathogens, including viruses and bacteria, opportunities to exploit them as routes for host cell entry. Although several studies have illuminated the molecular mechanisms underlying endocytic uptake of pathogenic and non-pathogenic cargoes, how these pathways are regulated to prevent uncontrolled uptake remains poorly understood. Here, using influenza A virus (IAV) as a tractable model cargo, we uncover the Wiskott–Aldrich syndrome protein and SCAR homolog (WASH) complex as a critical determinant of IAV entry.

The WASH complex (hereafter referred to as WASH) is a heteropentameric complex composed of WASH1, the branched F-actin nucleation-promoting factor (NPF), and its regulatory complex (SHRC), which includes CCDC53, SWIP, Strumpellin, and FAM21 (Derivery et al, 2009; Duleh and Welch, 2010; Gomez et al, 2012). Acting in concert with the retromer complex (retromer) at endosomes, WASH plays an essential role in cargo recycling by generating F-actin coats on endosomal membrane through activation of the actin-related protein 2/3 (Arp2/3), thereby providing the force required for the scission of cargo-containing tubules (Seaman et al, 2013; Gomez et al, 2012; Duleh and Welch, 2010; Burianek and Soderling, 2013). Although WASH is classically recruited to endosomes through the well-characterized interaction between FAM21 and the core retromer subunit VPS35 (Gomez and Billadeau, 2009; Harbour et al, 2012; Jia et al, 2012; Derivery et al, 2009), accumulating evidence suggests that WASH can also be targeted to endosomal membranes by additional, retromer-independent mechanisms (Simonetti et al, 2017; MacDonald et al, 2018; Kvainickas et al, 2017; Dostál et al, 2023). MacDonald et al demonstrated that the ESCRT-0 component HRS mediates WASH recruitment to endosomes and regulate WASH-dependent cargo recycling (MacDonald et al, 2018). More recently, Dostál et al reported that WASH can also be recruited to endosomes through direct binding of SWIP to phosphatidylinositol-3,5-bisphosphate [PI(3,5)P_2_]-rich endosomal membranes, independently of retromer association (Dostál et al, 2023).

In retromer-mediated recruitment of WASH, the long disordered “tail” of FAM21 (around 1100 amino acids, aa), which contains 21 repeating motifs composed of Leu-Phe and several acidic residues (Asp or Glu) termed LFa motifs, plays a critical role. While FAM21 interacts with WASH1 and Strumpellin through its N-terminal “head” domain (200 aa), which is important for WASH complex assembly and stability, the LFa motifs within its tail directly bind VPS35 via the final three repeats (Jia et al, 2012; Guo et al, 2024; Romano-Moreno et al, 2024). While the endosomal functions of WASH and retromer in cargo recycling are well established, recent advances using pathogens as endocytic cargoes indicate that their roles extend beyond canonical endosomal recycling functions. A recent study demonstrated that WASH1 drives clathrin-independent endocytosis of human papillomavirus type 16 (HPV16) through its NPF activity with no detectable involvement of the other WASH subunits, apart from a modest involvement of FAM21 (Brinkert et al, 2025). Together, the findings redefine WASH from an endosomal actin nucleator to a spatially and functionally versatile endocytic factor, prompting a reassessment of the prevailing view that the WASH complex functions as an obligate, inseparable unit dedicated to endosomal sorting and recycling.

Non-pathogen-based studies also highlighted functional divergence among WASH subunits. In particular, growing evidence suggest that FAM21 exerts functions beyond WASH. These include its separate role in regulating nuclear factor-κB transcriptional activity (Deng et al, 2015), its ability to localize to endosomes independently of WASH (Gomez and Billadeau, 2009), and a distinct role in recycling WASH from postlysosomal compartments after completion of H^+^ vacuolar-ATPase (v-ATPase) recycling in *Dictyostelium* (Park et al, 2013). Considering the divergent role of FAM21 from the other WASH subunits, WASH has been proposed to operate as two functional units: FAM21 and WASH1/CCDC53/Strumpellin, with SWIP serving as a connector (Park et al, 2013). Despite these insights, the precise WASH-independent functions of FAM21 remain incompletely defined.

Here, by interrogating the mechanistic involvement of TRIM62, a tripartite motif (TRIM)-containing protein, in IAV entry, we uncover a previously unrecognized functional connection between TRIM62 and WASH, and reveal unanticipated and functionally distinct roles for the WASH subunits and the retromer subunit VPS35 in viral endocytosis and downstream trafficking. By revealing how IAV entry is actively governed by the WASH-retromer dynamics, this work elucidates the mechanistic basis for the divergent behavior of FAM21 relative to other WASH subunits and highlights its critical regulatory role in viral trafficking.

## Results

### Focused RNAi screening of TRIM family proteins identifies TRIM62 as a key determinant of IAV entry

The starting point for this study was the identification of TRIM62 as a host mediator of IAV entry, revealed through a focused RNAi screen targeting 20 TRIM proteins that had been reported as candidate host factors in previous CRISPR-Cas screens against IAV infection (Maes et al, 2024; King et al, 2023; Yi et al, 2022; Zhou et al, 2021; Song et al, 2021; Anderson et al, 2021; Tran et al, 2020; Li et al, 2020; Han et al, 2018). The TRIM protein family in humans comprises over 80 distinct members, characterized by the presence of a conserved N-terminal RING domain with an E3 ubiquitin ligase activity, one or two B-box domains, a central coiled-coil domain (CC), and in some members, an additional C-terminal PRY-SPRY (P-S) domain (Hatakeyama, 2017). Beyond their involvement in diverse cellular processes, several TRIM proteins are known to play critical roles in viral infections, with most functioning as antiviral factors (Wei et al, 2024). However, the CRISPR-Cas screens indicated contrasting proviral roles of twenty TRIM proteins in IAV infection.

**Figure 1:**
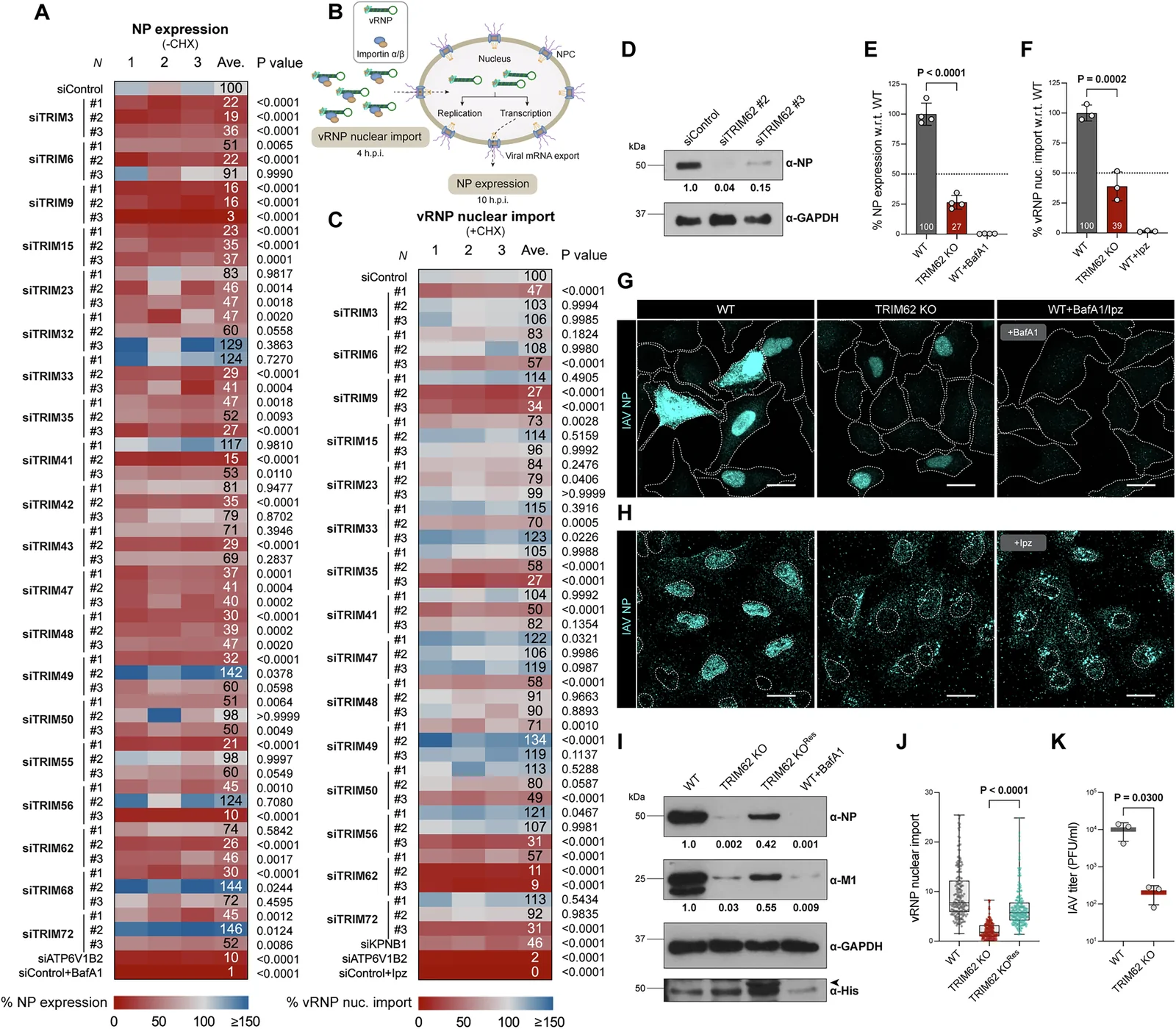
TRIM62 is required for IAV host cell entry. (**A**) Heatmap showing IAV infection in A549 cells infected with the X-31 (H3N2) strain (MOI = 0.01) in an siRNA screen targeting twenty TRIM proteins. *n* ≥ 2000 cells/siRNA. (**B**) Schematic of vRNP nuclear import and subsequent NP expression during IAV infection. (**C**) Heatmap showing normalized values of vRNP nuclear import (%) at 4 h.p.i. in an siRNA screen targeting fifteen TRIM proteins identified as supportive factors for IAV infection. *n* ≥ 800 cells/siRNA. (**D**) Western blot showing reduced NP levels at 10 h.p.i. in TRIM62 knockdown (KD) A549 cells compared to siControl. GAPDH was used as a loading control. The western blot is representative of three independent experiments. (**E**) Quantification showing reduced NP expression at 10 h.p.i. in TRIM62 knockout (KO) cells compared to WT A549 cells. *n* ≥ 5500 cells/sample (*N*  =  4 independent experiments; mean ± SD; error bars represent SD; one-way ANOVA with Dunnett’s multiple-comparisons test). *P* value for WT vs TRIM62 KO: *P*  < 0.0001. (**F**) Quantification showing reduced vRNP nuclear import at 4 h.p.i. in TRIM62 KO cells compared to WT A549 cells. *n* ≥ 1100 cells/sample (*N*  =  3 independent experiments; mean ± SD; error bars represent SD; one-way ANOVA with Dunnett’s multiple-comparisons test). *P* value for WT vs TRIM62 KO: *P*  = 0.0002. (**G**, **H**) Confocal microscopy images for Fig. 1E,F, showing IAV NP (cyan) for IAV infection (**G**) and vRNP nuclear import (**H**). Cell and nuclear boundaries are outlined. Scale bars, 20 µm. (**I**) Western blots showing restored viral NP and M1 levels at 10 h.p.i. in TRIM62 rescue cells compared to TRIM62 KO cells. Arrow marks the specific TRIM62-His band. The western blots are representative of three independent experiments. (**J**) Quantification showing restored vRNP nuclear import (NP intensity in nuclei) at 4 h.p.i. in TRIM62 rescue cells compared to TRIM62 KO cells. *n* = 200 cells/sample (*N* = 3 independent experiments; unpaired two-tailed t-test). *P* value for TRIM62 KO vs TRIM62 KO^Res^: *P*  = 8.10 × 10^−41^. Data are represented as box and whiskers plots where the center line indicates the median, the box boundaries represent the 25th and 75th percentiles (interquartile range), the whiskers extend to the minimum and maximum values, and all individual data points are overlaid. (**K**) Quantification of IAV titers by viral plaque assay. PFU, plaque-forming unit (*N*  =  3 independent experiments; mean ± SD; error bars represent SD; unpaired two-tailed *t*-test; *P*  = 0.03). Data in (**A**, **C**) were analyzed using one-way ANOVA with Dunnett’s multiple-comparisons test. *P* values are indicated in figure panel. *P* values < 0.05 were considered significant. Source data are available online for this figure.

To revalidate the involvement of these TRIM proteins as facilitators of IAV infection, we conducted a focused, imaging-based high-content RNAi screen, including genes that were identified in at least two independent screens. We included a non-targeting siRNA (siControl) as a negative control and a previously validated siRNA targeting the ATP6V1B2 subunit of the v-ATPase complex (siATP6V1B2) as a positive control (Banerjee et al, 2013). As an additional positive control, we included bafilomycin A1 (BafA1), a well-established inhibitor of the v-ATPase complex. The screen was performed in A549 cells, which are one of the most widely used lung-derived cell line for influenza research. In the screens, siRNA-transfected A549 cells were infected with the X-31 (H3N2) strain of IAV, and at 10 h post-infection (h.p.i.) the cells were processed for indirect-immunofluorescence (IIF) to detect the viral nucleoprotein (NP), an early viral protein. Using NP expression as a quantitative readout of infection, we identified 15 TRIM proteins whose depletion significantly reduced IAV infection (Fig. 1A). Since viral gene expression initiates only after the viral ribonucleoprotein complexes (vRNPs) of incoming viruses are imported into the nucleus—the final step of viral entry (Fig. 1B)—we addressed whether any of the identified proviral TRIM proteins are involved in viral entry into the host cells. To this end, we conducted a secondary RNAi screen targeting these candidate TRIM proteins for vRNP nuclear import. Cytoplasmic vRNPs that are released from the viral core upon uncoating at the late endosome, traffic into the nucleus using the importin-dependent nuclear import pathway (Eisfeld et al, 2015). Therefore, in our RNAi screen for vRNP nuclear import, we included an siRNA targeting the karyopherin/importin subunit β-1 (siKPNB1) and the importin-$\,\beta$ inhibitor importazole (Ipz) as positive controls. We also included the previously used siATP6V1B2 as an additional positive control. In this screen, siRNA-transfected cells were infected with purified viruses for 4 h to permit viral internalization and vRNP trafficking to the nucleus, followed by IIF detection of NP signals within the nuclear area. We added cycloheximide (CHX) to the infection medium to inhibit protein synthesis, thereby ensuring that the observed signals were derived from incoming vRNPs rather than newly synthesized viral proteins. In this screen, TRIM9, 35 and 62 were identified as hits involved in viral entry, among which TRIM62 emerged as the strongest candidate (Fig. 1C). Given its strong phenotypes in both the infection and vRNP nuclear import screens, we prioritized TRIM62 for further investigation to elucidate its mechanistic role in viral entry.

**Figure EV1:**
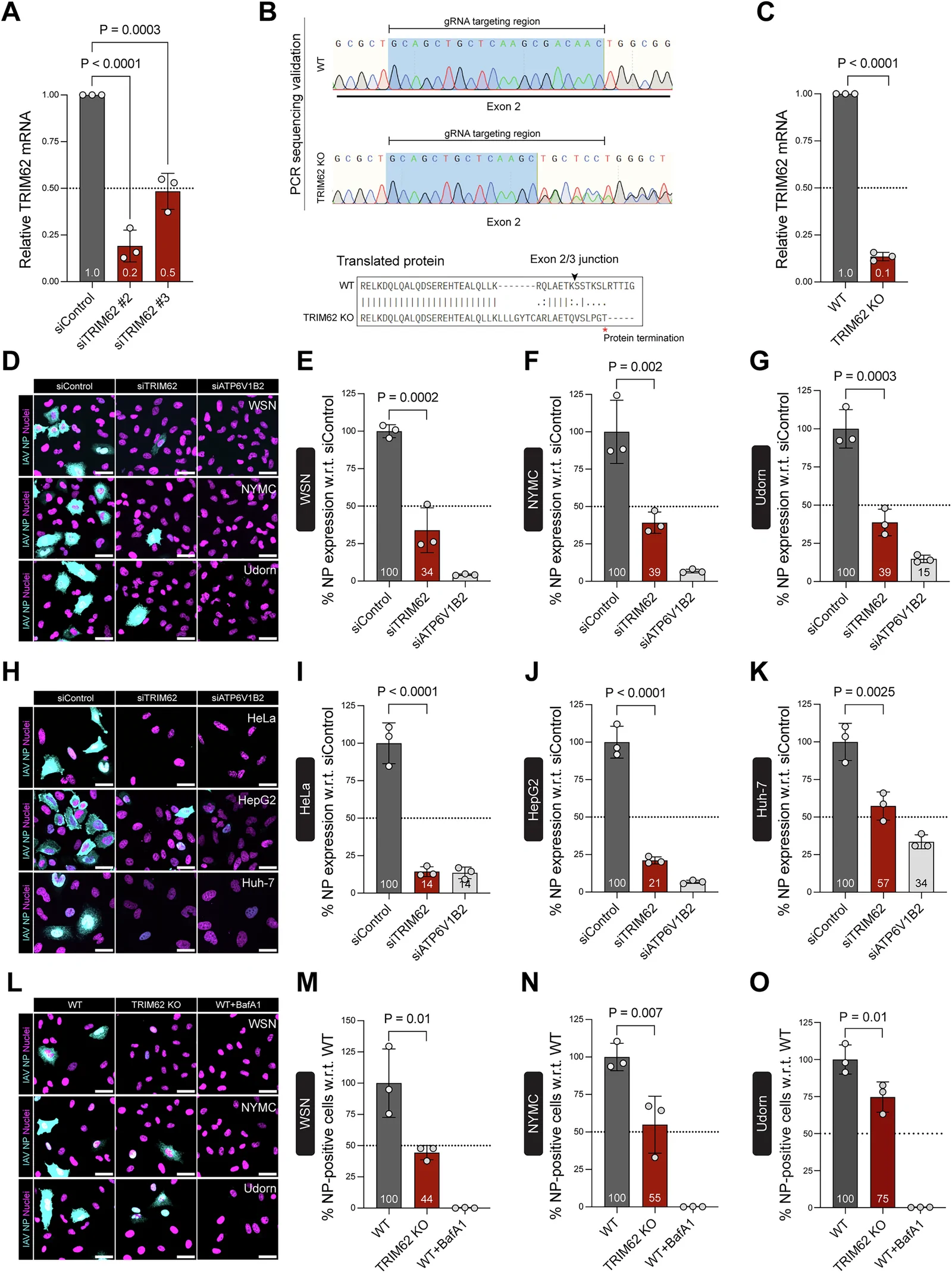
TRIM62 depletion attenuates IAV infection across different viral strains and cell lines. (**A**) Quantification of mRNA levels of *TRIM62* upon KD in A549 cells using two independent siRNAs targeting TRIM62 (*N*  =  3 independent experiments; mean ± SD; error bars represent SD; one-way ANOVA with Dunnett’s multiple-comparisons test). *P* values for siControl vs siTRIM62 #2: *P*  < 0.0001; siControl vs siTRIM62 #3: *P*  = 0.0003. (**B**) Chromatogram (top) showing DNA sequence downstream of the gRNA-targeted genomic region from WT and TRIM62 KO A549 cells. gRNA targeting region in exon 2 is highlighted. DNA sequences from WT and TRIM62 KO are translated using Expasy Translate tool and pairwise aligned using EMBOSS Needle tool (bottom), indicating premature protein termination in the KO cells. (**C**) Quantification of mRNA levels of *TRIM62* in TRIM62 KO cells compared to WT A549 cells (*N*  =  3 independent experiments; mean ± SD; error bars represent SD; unpaired two-tailed *t*-test; *P*  = 2.91 × 10⁻⁷). (**D**–**G**) High-content confocal images (**D**) and quantification of IAV infection in control and TRIM62 KD A549 cells infected with WSN (**E**), NYMC (**F**), and Udorn (**G**) for 10 h. NP (cyan), and nuclei (magenta). Scale bars, 20 µm. *n* ≥ 12,000 cells/assay (*N*  =  3 independent experiments; mean ± SD; error bars represent SD; one-way ANOVA with Dunnett’s multiple-comparisons test). *P* values for siControl vs siTRIM62: *P*  = 0.0002 (**E**); *P*  = 0.002 (**F**); *P*  = 0.0003 (**G**). (**H**–**K**) High-content confocal images (**H**) and quantification of IAV infection in HeLa (**I**), HepG2 (**J**), and Huh-7 (**K**) cells infected with IAV (X-31) for 10 h. Scale bars, 20 µm. *n* ≥ 4500 cells/assay (*N*  =  3 independent experiments; mean ± SD; error bars represent SD; one-way ANOVA with Dunnett’s multiple-comparisons test). *P* values for siControl vs siTRIM62: *P*  < 0.0001 (**I**, **J**); *P*  = 0.0025 (**K**). (**L**–**O**) High-content confocal images (**L**) and quantification of IAV infection in TRIM62 KO and WT A549 cells infected with WSN (**M**), NYMC (**N**), and Udorn (**O**) for 10 h. Scale bars, 20 µm. *n* ≥ 10,000 cells/assay (*N*  =  3 independent experiments; mean ± SD; error bars represent SD; one-way ANOVA with Dunnett’s multiple-comparisons test). *P* values for WT vs TRIM62 KO: *P*  = 0.01 (**M**); *P*  = 0.007 (**N**); *P*  = 0.01 (**O**).

Following the screens, we first sought to revalidate TRIM62’s role in viral gene expression by western blot analysis. Consistent with the screening results, silencing TRIM62 using two independent siRNAs led to a pronounced reduction in NP expression (Fig. 1D). As endogenous TRIM62 could not be reliably detected using commercially available antibodies, we assessed the knockdown (KD) efficiency of the siRNAs at the transcript level by quantitative reverse transcription polymerase chain reaction (qRT-PCR) (Fig. EV1A). Since siTRIM62 #2 demonstrated higher KD efficiency than the other, we used it (hereafter referred to as siTRIM62) for all subsequent RNAi-based experiments. Following KD experiments, we generated TRIM62 knockout (KO) in A549 cells using CRISPR-Cas9, and validated the KO cells by sequencing the gRNA-targeted genomic region and qRT-PCR (Fig. EV1B,C). Although sequencing results confirmed successful editing at the target site, the genome-edited cells still retained about 13% of TRIM62 transcripts relative to wild-type (WT) cells. Consistent with TRIM62 KD phenotypes, the KO cells exhibited a 73% reduction in IAV (X-31) infection and a 61% reduction in vRNP nuclear import, compared with WT cells (Fig. 1E–H). To further substantiate the involvement of TRIM62 in viral entry and viral gene expression, we performed rescue experiments by stably expressing His-tagged TRIM62 (TRIM62-His) in KO cells. Re-expression of TRIM62 restored both IAV infection and vRNP nuclear import to levels significantly higher than those observed in KO cells, confirming that the observed defects were specifically attributable to the loss of TRIM62 (Fig. 1I,J). Finally, we examined whether TRIM62 deficiency affects the production of infective progeny virions. Plaque assays performed with culture supernatants collected at 24 h.p.i. showed that TRIM62 KO cells produced nearly 50-fold fewer infectious IAV particles than parental cells, demonstrating a critical role for TRIM62 in the viral life cycle (Fig. 1K). Evaluation across multiple IAV strains, including WSN (H1N1, spherical), NYMC (H3N2, spherical), and Udorn (H3N2, filamentous), as well as different cell lines, including HeLa, HepG2, and Huh-7, further demonstrated that the role of TRIM62 in IAV infection is broadly conserved and not restricted to any particular viral strain or cellular background (Fig. EV1D–O).

Together, these findings establish TRIM62 as a previously unrecognized proviral host factor that promotes efficient IAV entry and productive infection across multiple viral strains and cellular contexts.

### TRIM62 facilitates IAV entry by regulating viral endocytosis and downstream trafficking

**Figure 2:**
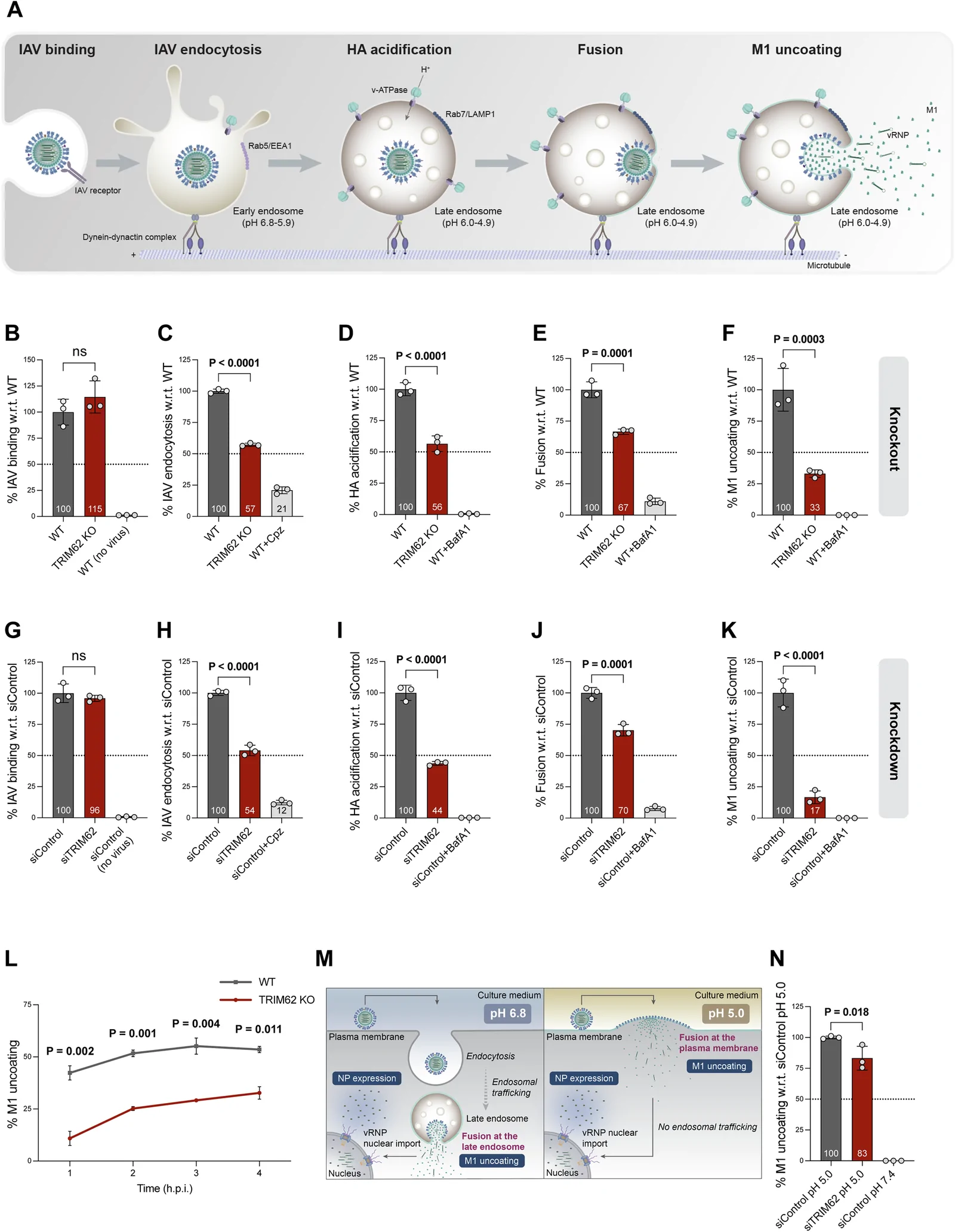
TRIM62 promotes IAV endocytosis and downstream trafficking. (**A**) Schematic showing key events during IAV vesicular trafficking including IAV binding, IAV endocytosis, HA acidification, fusion, and M1 uncoating. (**B**–**F**) Quantification of IAV binding (**B**), endocytosis (**C**), HA acidification (**D**), fusion (**E**), and M1 uncoating (**F**) at 0-, 30-, 60-, 90-, and 120-min post-infection, respectively, in WT A549 and TRIM62 KO cells. Chlorpromazine (Cpz) was included as a positive control in IAV endocytosis assay, whereas BafA1 was used as a positive control for HA acidification, fusion, and M1 uncoating. *n* ≥ 4000 cells/assay (*N*  =  3 independent experiments; mean ± SD; error bars represent SD; one-way ANOVA with Dunnett’s multiple-comparisons test). *P* values for WT vs TRIM62 KO: *P*  = 0.2742 (**B**); *P*  < 0.0001 (**C**, **D**); *P*  = 0.0001 (**E**); *P*  = 0.0003 (**F**). (**G**–**K**) Quantification of IAV binding (**G**), IAV endocytosis (**H**), HA acidification (**I**), fusion (**J**), and M1 uncoating (**K**) at 0, 30-, 60-, 90-, and 120-min post-infection, respectively, in control and TRIM62 KD A549 cells. *n* ≥ 4500 cells/assay (*N*  =  3 independent experiments; mean ± SD; error bars represent SD; one-way ANOVA with Dunnett’s multiple-comparisons test). *P* values for siControl vs siTRIM62: *P*  = 0.4829 (**G**); *P*  < 0.0001 (**H**, **I**); *P*  = 0.0001 (**J**); *P*  < 0.0001 (**K**). (**L**) Time-course analysis of M1 uncoating. Quantification of % M1 uncoating at 1, 2, 3, and 4 h.p.i. *n* ≥ 1000 cells/time point (*N*  =  3 independent experiments; mean ± SD; error bars represent SD; unpaired two-tailed *t*-test). *P* values for WT vs TRIM62 KO: *P*  = 0.002 (1 h.p.i.); *P*  = 0.001 (2 h.p.i.); *P*  = 0.004 (3 h.p.i.); *P*  = 0.011 (4 h.p.i.). (**M**) Schematic showing plasma membrane-bypass M1 uncoating and infection. IAV enters cells via endocytic routes at pH 6.8. When viral particles are induced to fuse at the plasma membrane under acidic condition (pH 5.0), the capsids disassemble, leading to M1 dispersal into the cytosol followed by vRNP nuclear import and NP expression. (**N**) Quantification of plasma membrane-bypass M1 uncoating (% cells with dispersed M1) at pH 5 and pH 7.4 (negative control) in control A549 and TRIM62 KD cells. *n* ≥ 1100 cells/sample (*N*  =  3 independent experiments; mean ± SD; error bars represent SD; one-way ANOVA with Dunnett’s multiple-comparisons test). *P* value for siControl vs siTRIM62: *P*  = 0.018 (pH 5.0). Source data are available online for this figure.

**Figure EV2:**
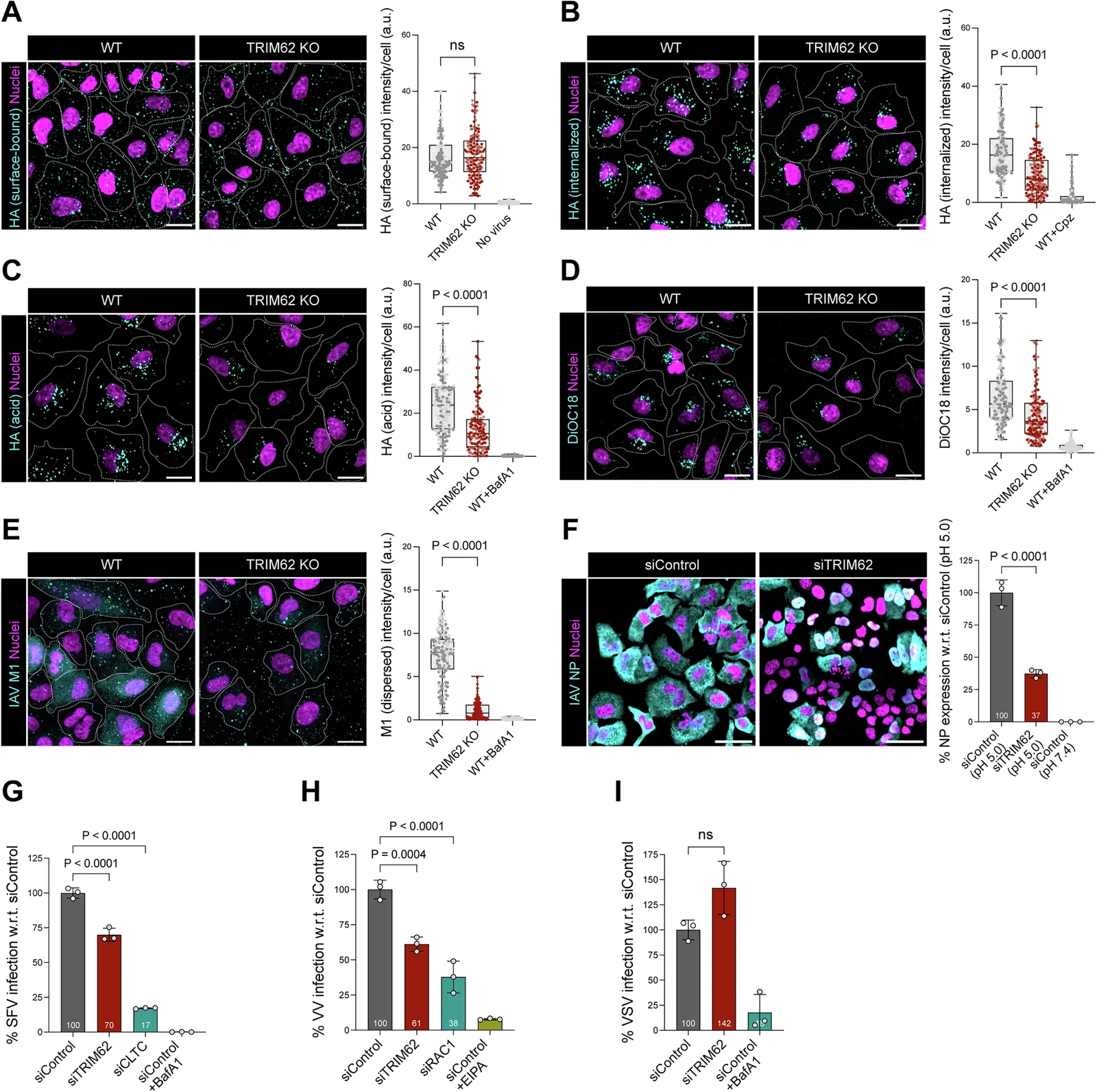
TRIM62 deficiency blocks IAV endocytosis and inhibits multiple viruses. (**A**) High-content confocal images (left) and intensity analysis (right) of surface-bound IAV particles detected by IIF against HA. HA (cyan) and nuclei (magenta). Cell boundaries are outlined. Scale bars, 20 µm. *n* = 158 cells/sample. (**B**) High-content confocal images (left) and intensity analysis (right) of internalized IAV particles (HA) at 30 min post-infection. HA (cyan) and nuclei (magenta). Scale bars, 20 µm. *n* = 156 cells/sample. (**C**) High-content confocal images (left) and intensity analysis (right) of acidified HA at 1 h.p.i. Scale bars, 20 µm. *n* = 146 cells/sample. (**D**) High-content confocal images (left) and intensity analysis (right) of DiOC18/R18-labeled IAV upon fusion at 1.5 h.p.i. DiOC18 (cyan) and nuclei (magenta). Scale bars, 20 µm. *n* = 152 cells/sample. (**E**) High-content confocal images (left) and intensity analysis (right) of cytosolic M1 (dispersed) upon M1 uncoating at 2 h.p.i. M1 (cyan) and nuclei (magenta). Scale bars, 20 µm. *n* = 150 cells/sample. (**F**) High-content confocal images (left) and quantification (right) of plasma membrane-bypass infection (NP expression) at pH 5 and pH 7.4 (negative control) in control and TRIM62 KD A549 cells. *n* ≥ 4500 cells/assay (*N*  =  3 independent experiments; mean ± SD; error bars represent SD; one-way ANOVA with Dunnett’s multiple-comparisons test). *P* values for siControl vs siTRIM62: *P*  < 0.0001 (pH 5.0). (**G**) Quantification of SFV infection in control and TRIM62 KD A549 cells at 24 h.p.i. *n* ≥ 7000 cells/siRNA (*N*  =  3 independent experiments; mean ± SD; error bars represent SD; one-way ANOVA with Dunnett’s multiple-comparisons test). *P* values for siControl vs siTRIM62: *P*  < 0.0001; siControl vs siCLTC: *P*  < 0.0001. (**H**) Quantification of VV infection in control and TRIM62 KD A549 cells at 24 h.p.i. *n* ≥ 7000 cells/siRNA (*N*  =  3 independent experiments; mean ± SD; error bars represent SD; one-way ANOVA with Dunnett’s multiple-comparisons test). *P* values for siControl vs siTRIM62: *P*  = 0.0004; siControl vs siRAC1: *P*  < 0.0001. (**I**) Quantification of VSV infection in control and TRIM62 KD A549 cells at 10 h.p.i. *n* ≥ 5000 cells/siRNA (*N*  =  3 independent experiments; mean ± SD; error bars represent SD; one-way ANOVA with Dunnett’s multiple-comparisons test). *P* values for siControl vs siTRIM62: *P*  = 0.0664. Data in (**A**–**E**) are represented as box and whiskers plots. The center line represents the median, the box boundaries correspond to the 25th and 75th percentiles (interquartile range), the whiskers extend to the minimum and maximum values, and all individual data points are overlaid (*N*  =  3 independent experiments; one-way ANOVA with Dunnett’s multiple-comparisons test). *P* values for WT vs TRIM62 KO: *P*  = 0.1420 (**A**); *P*  < 0.0001 (**B**–**E**). *P* < 0.05 (*), *P* < 0.01 (**), *P* < 0.001 (***), *P* < 0.0001 (****). *P* values < 0.05 were considered significant.

Following the identification of TRIM62 as a viral entry-promoting host factor, we next sought to determine the precise step of the viral entry process at which it functions. IAV entry is a multi-step process that includes virion binding to the cell surface, endocytic uptake, HA acidification and activation by the acidic environment of the late endosome, fusion of the viral envelope with the late endosomal limiting membrane, and M1 uncoating, which leads to capsid disassembly and vRNP release into the cytosol. The sequential entry steps of IAV involving vesicular trafficking, i.e., prior to vRNP nuclear import is shown in the schematic (Fig. 2A). To delineate the specific role of TRIM62 in IAV entry, we employed quantitative high-content, imaging-based viral entry assays that we had previously developed (Banerjee et al, 2013), to systematically interrogate each sequential step of the viral entry pathway. We found that although virion binding to the plasma membrane was unaffected in TRIM62-deficient cells (Figs. 2B,G and EV2A), endocytosis of surface-bound virions (30 min post-infection) was reduced by 43% and 46% in TRIM62 KO and KD cells, respectively (Figs. 2C,H and EV2B). Consistent with the endocytosis defects, TRIM62-deficient cells showed an impairment at all downstream post-endocytic entry steps including HA acidification (1 h.p.i.), fusion (1.5 h.p.i.), and M1 uncoating (2 h.p.i.) (Figs. 2D–F,I–K and EV2C–E). Notably, both KO and KD cells displayed comparable reductions across IAV entry steps. Since M1 uncoating phenotypes prominently reflected viral endocytosis defects and were readily quantifiable, we used uncoating as a readout for viral entry in many of our subsequent experiments. Was reduction in M1 uncoating (examined at 2 h.p.i.) due to delayed kinetics of viral entry? Time-course analysis showed consistent impairment in uncoating, excluding this possibility (Fig. 2L).

Of all entry steps examined, M1 uncoating (probed for post-fusion cytosolic M1 dispersal) displayed the most pronounced phenotypes with 67% and 83% reductions in uncoating in KO and KD cells, respectively (Fig. 2F,K), relative to controls. We next asked whether TRIM62 directly facilitates M1 uncoating post-fusion or the uncoating defect observed in TRIM62-deficient cells is a consequence of impaired endocytic trafficking. To clarify this, viral fusion was induced at the plasma membrane (PM) using a pH 5.0 medium, enabling direct capsid delivery to the cytosol, thereby bypassing entry through the endocytic route (Fig. 2M). PM-bypass uncoating exhibited only a modest decrease in viral uncoating in TRIM62 KD cells compared to control cells, with only 17% of the cells showing defects in uncoating (Fig. 2N). Thus, the uncoating defect observed upon TRIM62 silencing when the virus was allowed to enter through the endocytic pathway was largely rescued when viral fusion was induced directly at the PM. This suggested that TRIM62 primarily functions in viral endocytic trafficking rather than in uncoating process itself. Next, we sought to investigate whether TRIM62 functions exclusively during viral entry or it has additional roles in the post-entry stages of the viral life cycle. This was prompted by the observation that TRIM62 KD resulted in a substantially greater reduction in NP expression than could be explained by defects in endocytosis alone. Induced viral fusion at the PM led to a significant decrease in NP expression in TRIM62 KD cells, highlighting its additional role in the post-entry stages of infection (Fig. EV2F). Is the role of TRIM62 restricted to IAV infection alone? To address this, we examined infection by other viruses including Semliki Forest virus (SFV), vaccinia virus (VV), and vesicular stomatitis virus (VSV) in A549 cells. While TRIM62 depletion reduced SFV and VV infection by 30% and 39%, respectively (Fig. EV2G,H), VSV infection remained largely unaffected (Fig. EV2I). These findings indicate that TRIM62 functions as a proviral host factor that facilitates infection by multiple viruses, although its requirement varies among different viruses.

Although we found that TRIM62 functions at both endocytic and post-entry stages of IAV infection, we focused this study on elucidating its role in viral endocytic uptake and trafficking, given the largely unexplored involvement of TRIM family proteins in viral entry pathways.

### TRIM62 facilitates WASH-dependent lipid raft-mediated endocytic trafficking

**Figure 3:**
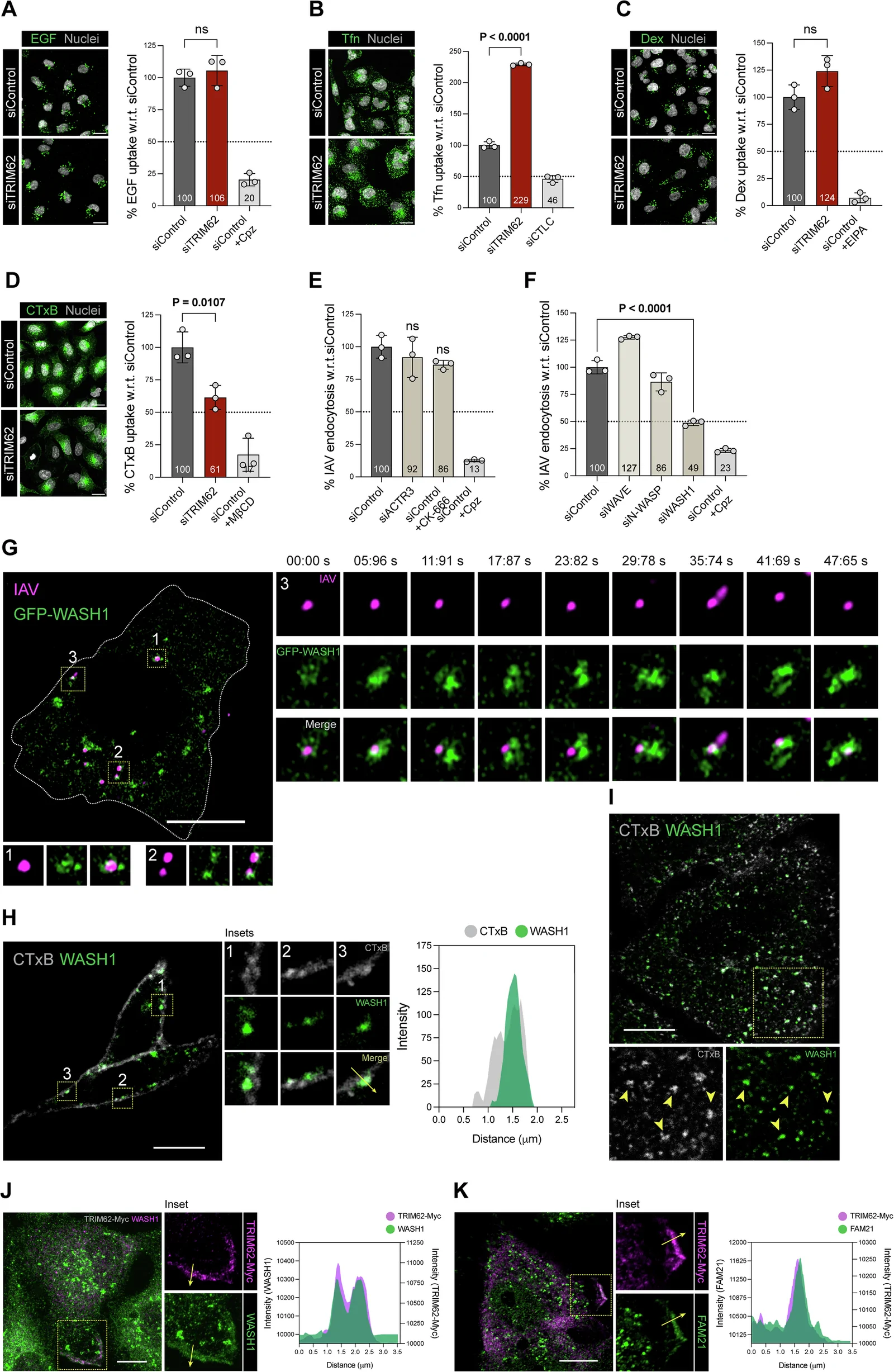
TRIM62 and WASH1 drive IAV endocytosis through a clathrin-independent pathway. (**A**–**D**) High-content confocal images (left; nuclei, gray and cargo, green) and quantification (right) of EGF (**A**), Tfn (**B**), Dex (**C**), and CTxB (**D**) uptake in TRIM62 KD A549 cells compared to controls. Scale bars, 20 µm. *n* ≥ 3000 cells/assay (*N*  =  3 independent experiments; mean ± SD; error bars represent SD; one-way ANOVA with Dunnett’s multiple-comparisons test). *P* values for siControl vs siTRIM62: *P*  = 0.6473 (**A**); *P*  <  0.0001 (**B**); *P*  = 0.0605 (**C**); *P*  = 0.0107 (**D**). (**E**) Quantification of IAV endocytosis in control, ACTR3 KD, and CK-666-treated A549 cells. *n* ≥ 1500 cells/sample (*N*  =  3 independent experiments; mean ± SD; error bars represent SD; one-way ANOVA with Dunnett’s multiple-comparisons test). *P* values for siControl vs siACTR3: *P*  = 0.5842; siControl vs siControl+CK-666: *P*  = 0.22. (**F**) Quantification of IAV endocytosis in control and WAVE, N-WASP, and WASH1 KD A549 cells. *n* ≥ 1500 cells/sample (N = 3 independent experiments; mean ± SD; error bars represent SD; one-way ANOVA with Dunnett’s multiple-comparisons test). *P* values for siControl vs siWASH1: *P*  <  0.0001. (**G**) Representative high-resolution, live-cell images of GFP-WASH1 (green)-expressing A549 cell during IAV (magenta) entry. The left panel shows a representative cell with three virus-containing regions (boxed and numbered 1–3), with enlarged views of regions 1 and 2 are shown below. Time-lapse images of region 3 (right) illustrate the dynamic association of GFP-WASH1 with an incoming IAV particle over time. Time is indicated in seconds (s). (**H**) Confocal microscopy image (left) showing surface-bound CTxB (gray) and WASH1 (green). Insets show colocalization of CTxB with WASH1 at the plasma membrane. Fluorescence intensity profiles of CTxB and WASH1 along the yellow line are shown (right). Scale bars, 10 µm. (**I**) Confocal microscopy image showing CTxB and WASH1 10 min post-internalization of CTxB. Insets show the distribution of internalized CTxB and WASH1, and yellow arrowheads indicate their colocalization. Scale bars, 10 µm. (**J**) High-resolution microscopy image (left) of a representative cell showing the presence of endogenous WASH1 (green) and overexpressed TRIM62-Myc (magenta) at lamellipodium. Insets show the colocalization of WASH1 and TRIM62-Myc at the lamellipodium. Fluorescence intensity profiles of WASH1 and TRIM62-Myc along the yellow line are shown (right). Scale bars, 10 µm. (**K**) High-resolution microscopy image (left) of a representative cell showing the presence of endogenous FAM21 (green) and overexpressed TRIM62-Myc (magenta) at lamellipodium. Insets show the colocalization of FAM21 and TRIM62-Myc at the lamellipodium. Fluorescence intensity profiles of FAM21 and TRIM62-Myc along the yellow line are shown (right). Scale bars, 10 µm. Representative images from three independent experiments are shown for Fig. 3G–K. Source data are available online for this figure.

IAV exploits multiple endocytic pathways for entry, including CME and non-clathrin pathways that include macropinocytosis and lipid raft-dependent pathways (de Vries et al, 2011; Matlin et al, 1981; Li et al, 2022; Sieczkarski and Whittaker, 2002). Since we observed that TRIM62 deficiency impairs IAV endocytosis, we next investigated whether it selectively modulates specific endocytic pathway(s). To address this, we analyzed the cellular uptake of model pathway-specific cargoes in TRIM62 KD cells. For CME, we assessed epidermal growth factor (EGF) and transferrin (Tfn) uptake for 30 min and 10 min, respectively, with chlorpromazine (Cpz) or an siRNA targeting the clathrin heavy chain (siCLTC) serving as positive controls, respectively. While EGF internalization remained unaffected, Tfn uptake was significantly increased in TRIM62-deficient A549 cells (Fig. 3A,B). Although EGF is primarily a CME cargo, it can also enter cells via non-clathrin pathways (Sigismund et al, 2005), whereas Tfn is a bona fide CME cargo. These data indicate that the IAV endocytosis defect in TRIM62-deficient cells was not due to impaired viral entry through CME.

**Figure EV3:**
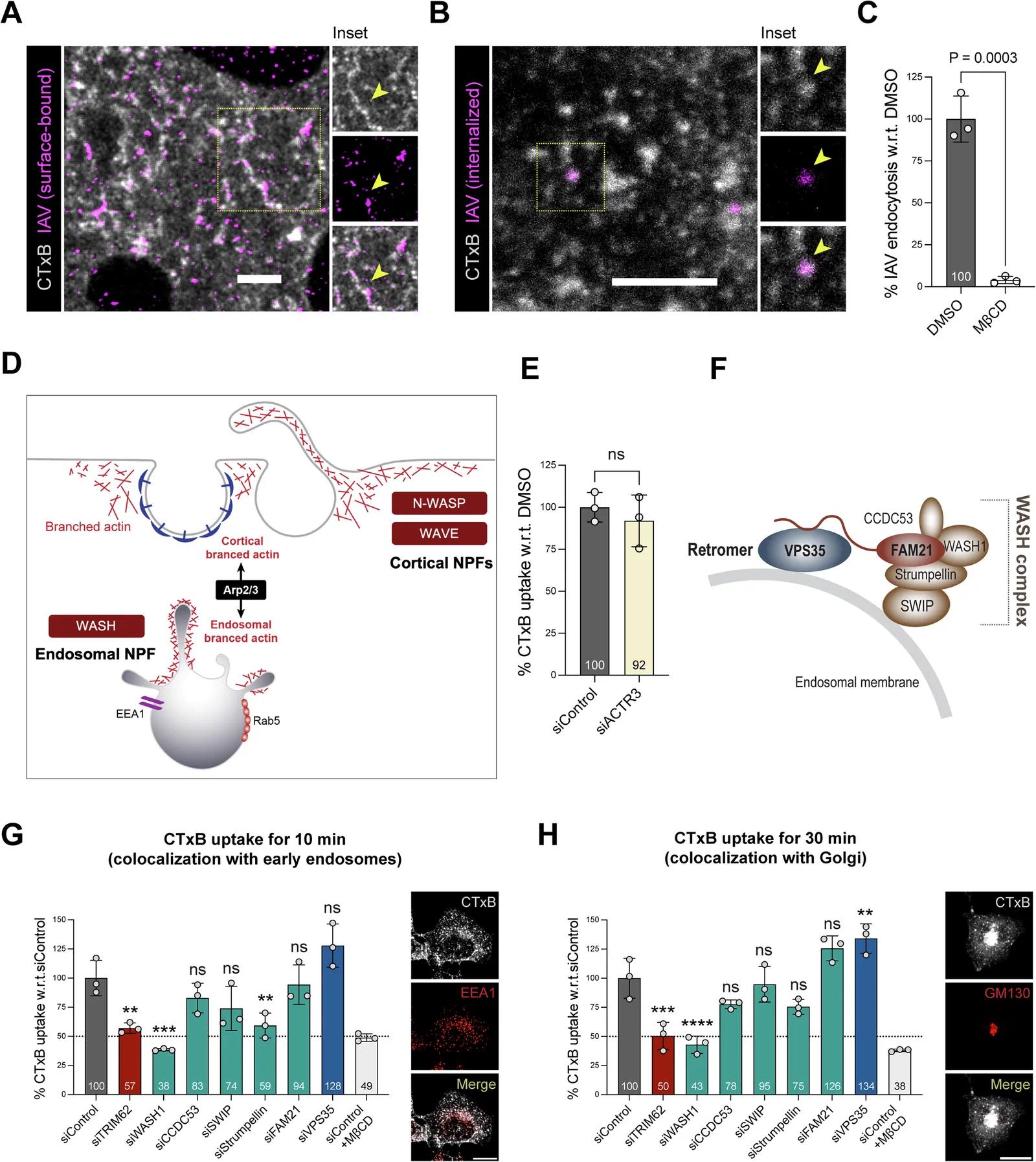
Association of IAV with CTxB-positive domains and concerted regulation of CTxB uptake by TRIM62 and WASH1. (**A**) Confocal microscopy images showing surface-bound CTxB (gray) and IAV (magenta). Insets show the distribution of CTxB and IAV on lipid rafts and yellow arrowheads indicate colocalization. Scale bars, 5 µm. Representative images from three independent experiments are shown. (**B**) Confocal microscopy images showing CTxB (gray) and IAV (magenta), 10 min post-internalization. Insets show the distribution of internalized CTxB and IAV and yellow arrowheads indicate colocalization. Scale bars, 2 µm. Representative images from three independent experiments are shown. (**C**) Quantification of IAV endocytosis in DMSO-treated and MβCD-treated A549 cells. *n* ≥ 500 cells/sample (*N*  =  3 independent experiments; mean ± SD; error bars represent SD; unpaired two-tailed *t*-test; *P*  = 0.0003). (**D**) Schematic representation of branched F-actin nucleation by Arp2/3 complex and cortical or endosomal NPFs. (**E**) Quantification of CTxB uptake in ACTR3 KD A549 cells at 30 min. *n* ≥ 2000 cells/siRNA (*N*  =  3 independent experiments; mean ± SD; error bars represent SD; unpaired two-tailed *t*-test; *P*  = 0.4737). (**F**) Schematic showing the recruitment of pentameric WASH complex via retromer at endosomal surface, specifically mediated by the interaction of FAM21 with VPS35. (**G**, **H**) Quantification of CTxB uptake in control and KD A549 cells post 10 min (**G**) and 30 min (**H**) internalization. *n* ≥ 1000 cells/sample. Images show CTxB uptake through colocalization of CTxB with EEA1-positive endosomes at 10 min (**G**) and with GM130-positive Golgi compartments at 30 min (**H**), respectively. Scale bars, 20 µm. Data in (**G**) were analyzed using one-way ANOVA with Dunnett’s multiple-comparisons test. *N*  =  3 independent experiments; mean ± SD; error bars represent SD. *P* values for siControl vs siTRIM62: *P*  = 0.0051; siControl vs siWASH1: *P*  = 0.0001; siControl vs siCCDC53: *P*  = 0.4889; siControl vs siSWIP: *P*  = 0.1273; siControl vs siStrumpellin: *P*  = 0.0079; siControl vs siFAM21: *P*  = 0.9948; siControl vs siVPS35: *P*  = 0.0924. Data in (**H**) were analyzed using one-way ANOVA with Dunnett’s multiple-comparisons test. *N*  =  3 independent experiments; mean ± SD; error bars represent SD. *P* values for siControl vs siTRIM62: *P*  = 0.0002; siControl vs siWASH1: *P*  < 0.0001; siControl vs siCCDC53: *P*  = 0.1089; siControl vs siSWIP: *P*  = 0.9920; siControl vs siStrumpellin: *P*  = 0.0684; siControl vs siFAM21: *P*  = 0.0537; siControl vs siVPS35: *P*  = 0.0073. *P* < 0.05 (*), *P* < 0.01 (**), *P* < 0.001 (***), *P* < 0.0001 (****). *P* values < 0.05 were considered significant.

To determine TRIM62’s specific involvement in IAV entry via non-clathrin pathways, we examined macropinocytosis and lipid raft-mediated endocytosis. To assess macropinocytosis, we monitored the uptake of 70 kDa dextran (Dex), which specifically internalizes via the macropinocytic pathway. While the macropinocytosis inhibitor 5-(N-ethyl-N-isopropyl)-amiloride (EIPA) almost completely inhibited Dex uptake in A549 cells, TRIM62 depletion had no effect (Fig. 3C). However, uptake of CTxB, a lipid raft-dependent cargo, was reduced by 39% in TRIM62-deficient A549 cells (Fig. 3D), recapitulating a comparable defect in IAV endocytosis (Fig. 2H). Moreover, we observed colocalization of surface-bound IAV and lipid raft domains at the plasma membrane (as probed by CTxB), as well as colocalization of endocytosed IAV particles with CTxB, suggesting that IAV engages lipid raft pathway during entry, similar to CTxB (Fig. EV3A,B). Since lipid raft-mediated endocytosis is cholesterol dependent, we examined whether IAV endocytosis is sensitive to cholesterol depletion. To deplete cholesterol, we treated A549 cells with methyl-β-cyclodextrin (MβCD). In MβCD-treated cells, we observed a severe reduction in viral internalization, underscoring a critical role for cholesterol-dependent, raft-mediated pathways in viral entry (Fig. EV3C). Together, these findings indicate that TRIM62 regulates a clathrin-independent, but cholesterol-dependent, lipid raft-mediated endocytic pathway exploited by IAV.

Since clathrin-independent pathways of IAV entry are less studied compared to CME and since several non-clathrin pathways rely on branched actin dynamics, we next tested whether branched actin networks are required for viral uptake. To address this, we perturbed two key classes of branched actin regulators: the actin-nucleating Arp2/3 complex and its nucleation-promoting factors (NPFs) (Fig. EV3D). Disruption of the Arp2/3 complex, either by siRNA targeting the Arp3 (ACTR3) subunit or by pharmacological inhibition with CK-666, had no effect on IAV uptake (Fig. 3E). Similarly, ACTR3 depletion did not affect CTxB uptake (Fig. EV3E), suggesting that both CTxB and IAV internalization in A549 cells are not strongly dependent on branched actin networks. However, analysis of NPFs showed that while depletion of the surface regulators WAVE2 or N-WASP had only mild effects, loss of the endosomal NPF WASH1 markedly impaired viral internalization (Fig. 3F), highlighting its crucial role in IAV entry. To examine whether WASH1 associates with incoming viral particles during early entry stage, we performed time-lapse video microscopy. To this end, we labeled purified IAV particles with lipophilic fluorescent dye R18 and added to A549 cells expressing green fluorescent protein (GFP)-tagged mouse WASH1 (GFP-WASH1). At 10 min post virus addition, we performed high-resolution live-cell microscopy and observed colocalization of incoming virus particles with GFP-WASH1, further indicating the involvement of WASH1 in IAV entry (Fig. 3G). Based on our findings that TRIM62 facilitates IAV entry via a lipid-raft pathway and that WASH1 associates with IAV during internalization, we next examined whether WASH1 also associates with CTxB and supports its uptake. Interestingly, we observed pronounced colocalization of WASH1 with CTxB both when CTxB was bound at the cell surface in cold and at 10 min post internalization (Fig. 3H,I). The pentameric WASH complex comprises the NPF WASH1 and the SHRC which includes CCDC53, SWIP, Strumpellin, and FAM21. WASH is recruited to endosomal membrane via the retromer subunit VPS35 (Fig. EV3F). To determine whether all WASH subunits contribute to the regulation of the lipid raft pathway, we individually depleted each subunit, as well as the retromer component VPS35. KD efficiency of the siRNAs was confirmed by RT-PCR (Appendix Fig. S1A). CTxB uptake was then monitored in cells depleted of WASH subunits or VPS35 at 10- and 30-min post-internalization, assessing colocalization with the early endosomal marker EEA1 and Golgi marker GM130, respectively. We found that while WASH1 is a critical regulator of CTxB uptake, SHRC is dispensable (Fig. EV3G,H). Strikingly, VPS35 depletion enhanced CTxB uptake, in contrast to the effect of WASH1 depletion (Fig. EV3H). The differential involvement of the SHRC in IAV and CTxB uptake is particularly intriguing, which suggests that WASH1 may have a function that is at least partially independent of the complex during CTxB internalization. Although WASH1 is generally considered to function together with SHRC, our findings indicate that individual WASH subunits play distinct, cargo-specific roles, specifically in CTxB uptake. Notably, we did not detect a requirement for branched actin polymerization during either IAV or CTxB internalization in A549 cells, raising the possibility that WASH1 supports these endocytic pathways through a non-canonical scaffolding role rather than its well-established function in Arp2/3-mediated branched actin assembly. Why individual WASH subunits function differently during the uptake of these cargoes, and why the SHRC appears dispensable for CTxB uptake, remains an open question and will require further mechanistic investigations.

However, given the shared requirement of both TRIM62 and WASH1 in IAV and CTxB uptake, and the observed colocalization of WASH1 with both the cargoes during their internalization, we next addressed whether TRIM62 and WASH1 associate within cells. Airyscan high-resolution microscopy in A549 cells revealed an enrichment of TRIM62-Myc at the branched actin-rich lamellipodium, where endogenous WASH1 was also detected (Fig. 3J). We also found colocalization of another WASH subunit, FAM21, with TRIM62 at the lamellipodium (Fig. 3K). However, no colocalization was detected at the perinuclear region where the WASH typically associates with endosomal compartments. Although the lamellipodial presence of WASH has been debated (Derivery et al, 2009; Linardopoulou et al, 2007), our observations in A549 cells show its presence at the lamellipodium, where it colocalizes with TRIM62.

Collectively, our findings reveal that both TRIM62 and WASH1 facilitate the uptake of IAV and CTxB, that WASH1 associates with these cargoes during internalization, and that both factors colocalize at branched-actin rich lamellipodia, suggesting shared functions in the lipid raft-dependent endocytic pathways utilized by both IAV and CTxB.

### WASH subunits and VPS35 differentially regulate IAV entry

The functional intersection between TRIM62 and the endosomal cargo sorting machinery including WASH and VPS35 in the lipid raft pathway prompted us to examine their potential involvement in IAV infection. We next evaluated the effects of depleting WASH subunits and VPS35 on IAV infection and found an involvement of all WASH subunits and VPS35 in viral NP expression. Surprisingly, we found striking functional dichotomy among WASH subunits: depletion of WASH1, CCDC53, SWIP and Strumpellin suppressed NP expression by 56–86%, whereas loss of FAM21, the WASH subunit that directly engages VPS35, boosted NP expression by 53%. Like FAM21, VPS35 depletion also enhanced infection (Appendix Fig. S1B), consistent with their functional relationship and suggesting the existence of an antiviral FAM21-VPS35 axis.

**Figure 4:**
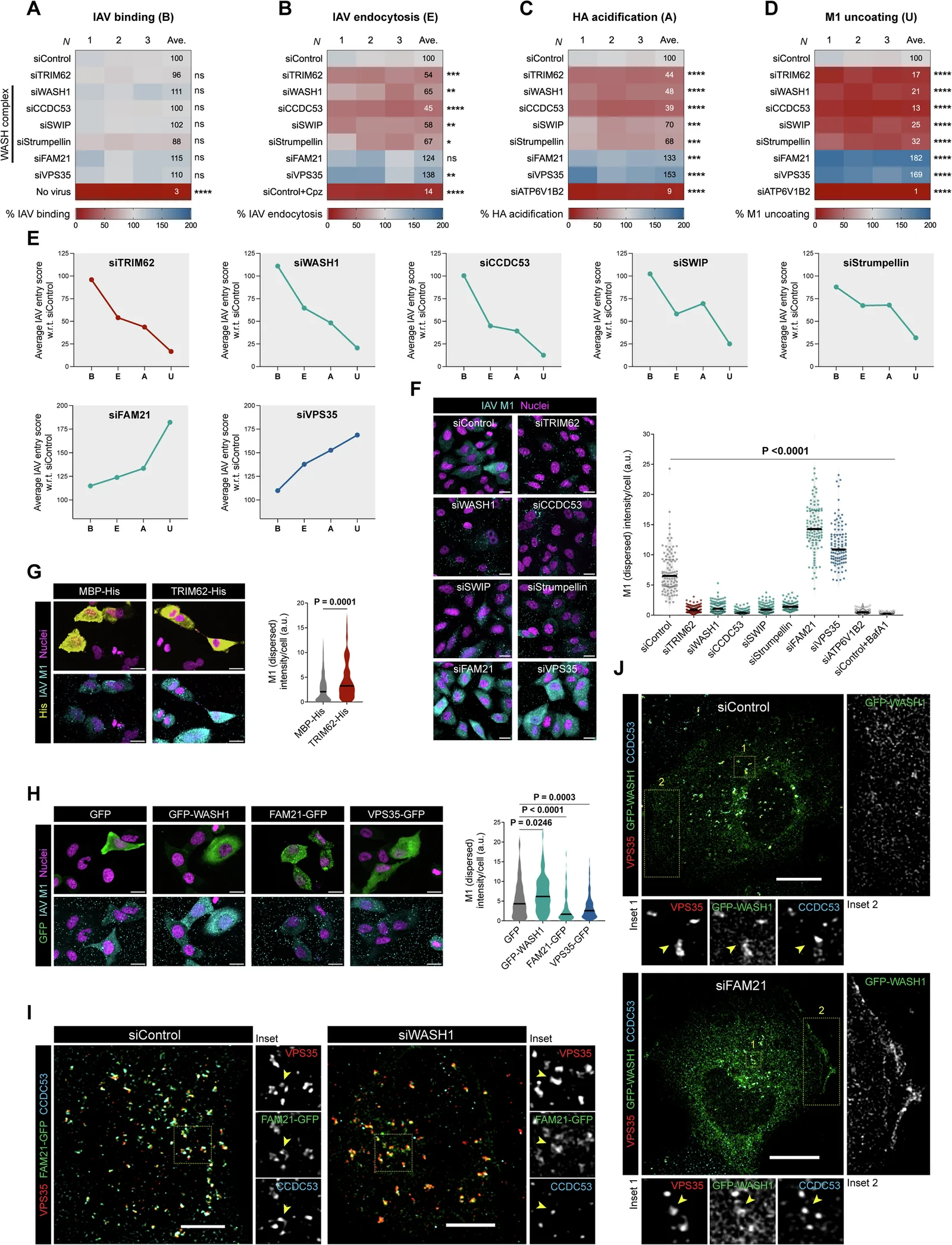
VPS35 and WASH subunits exert opposing effects on IAV entry. (**A**–**D**) Heatmaps showing IAV binding (**A**), IAV endocytosis (**B**), HA acidification (**C**), and M1 uncoating (**D**) in siRNA screens targeting WASH subunits and VPS35. *n* ≥ 500 cells/siRNA. (**E**) Line graphs showing average values (derived from Fig. 4**A**–**D**) for IAV binding (B), endocytosis (E), HA acidification (A), and M1 uncoating (U) upon KD of individual factors. (**F**) High-content confocal images (left; IAV M1, cyan and nuclei, magenta) and scatter plot (right) showing the quantification of cytosolic M1 (dispersed) intensity per cell. Scale bars, 20 µm. *n* ≥ 99 cells/siRNA (*N* = 3 independent experiments; one-way ANOVA with Dunnett’s multiple-comparisons test). Individual data points are overlaid on violin plots. *P* values for each comparison: *P*  <  0.0001. (**G**) High-content confocal images (left; IAV M1, cyan; His, yellow and nuclei, magenta) and violin plots (right) showing the quantification of cytosolic M1 (dispersed) intensity in cells expressing MBP-His or TRIM62-His at 120 min post-infection. Scale bars, 20 µm. *n* = 120 cells/sample (*N*  =  3 independent experiments; unpaired two-tailed *t*-test; *P*  = 0.0001). Violin plots show the distribution of the data. (**H**) High-content confocal images (left; IAV M1, cyan; GFP, green and nuclei, magenta) and violin plot (right) showing the quantification of cytosolic M1 (dispersed) intensity in cells expressing GFP and GFP-tagged WASH1, FAM21 or VPS35 at 120 min post-infection. GFP intensities are differently adjusted for optimum visualization. Scale bars, 20 µm. *n* ≥ 125 cells/sample. (*N*  =  3 independent experiments; one-way ANOVA with Dunnett’s multiple-comparisons test). Violin plots show the distribution of the data. *P* values for GFP vs GFP-WASH1: *P*  = 0.0246; GFP vs FAM21-GFP: *P*  < 0.0001; GFP vs VPS35-GFP: *P*  = 0.0003. (**I**) Super-resolution (SIM) images of control and WASH1 KD A549 cells showing FAM21-GFP (green), CCDC53 (cyan), and VPS35 (red). Insets show the distribution of FAM21-GFP, CCDC53, and VPS35 on endosomes. Yellow arrowheads indicate colocalization. Scale bars, 5 µm. Representative images from three independent experiments are shown (**J**) Super-resolution (SIM) images of control and FAM21 KD A549 cells showing GFP-WASH1 (mouse; green), CCDC53 (cyan), and VPS35 (red). Insets show the distribution of GFP-WASH1, CCDC53, and VPS35 on endosomes and cell surface. Yellow arrowheads indicate colocalization. Scale bar, 10 µm. Representative images from three independent experiments are shown. Data in (**A**–**D**) were analyzed using one-way ANOVA with Dunnett’s multiple-comparisons test. *P* < 0.05 (*), *P* < 0.01 (**), *P* < 0.001 (***), *P* < 0.0001 (****). *P* values < 0.05 were considered significant. Source data are available online for this figure.

After observing the contrasting roles of WASH subunits (except FAM21) and the VPS35-FAM21 axis in IAV infection, we next investigated whether these factors contribute to early viral entry, thereby influencing NP expression at later stages. To this end, we monitored sequential steps of IAV entry, beginning with viral binding to the cell surface, followed by internalization and endosomal trafficking in cells deficient of WASH subunits or VPS35. Along with these factors, TRIM62 was also evaluated, given our earlier results pointing to a potential link with WASH in viral endocytic trafficking. We observed that while virus binding was unaffected, IAV endocytosis and the downstream entry steps were altered in A549 cells deficient of WASH subunits and VPS35 (Fig. 4A–D). Consistent with infection phenotypes, depletion of the WASH subunits (WASH1, CCDC53, SWIP, and Strumpellin) and TRIM62, blocked viral entry, whereas suppression of FAM21 and VPS35 showed an opposite phenotype (Fig. 4A–D). To gain clearer insight into the role of each factor, we plotted average values for each entry step as line graphs. This visualization further emphasized a striking dichotomy: while VPS35 and FAM21 depletion progressively boosted IAV entry, depletion of TRIM62, WASH1 and other SHRC subunits (CCDC53, SWIP, and Strumpellin) led to a gradual decline (Fig. 4E). Quantification of cytosolic M1 intensity at the single-cell level confirmed this perturbation and supported the population-level trends (Fig. 4F). Microscopy images revealed a stark divergence in uncoating (M1 dispersal) phenotypes between FAM21/VPS35 depletion and depletion of TRIM62 and other WASH subunits (Fig. 4F). To further confirm the roles of some of these key factors in IAV entry, we performed M1 uncoating assay in cells overexpressing them. Overexpression experiments further validated their respective pro- or antiviral roles in IAV entry: TRIM62-His and GFP-WASH1 significantly boosted uncoating, whereas FAM21-GFP and VPS35-GFP markedly suppressed it (Fig. 4G,H).

We next examined how silencing individual WASH subunits or VPS35 affects the protein levels of the other subunits in A549 cells. Western blot analysis showed that depletion of WASH1 or other SHRC subunits reduce the levels of remaining components, consistent with their function as an interdependent complex. In contrast, VPS35 depletion did not alter the levels of WASH1 or SHRC subunit (CCDC53, FAM21, and SWIP) (Appendix Fig. S1C), indicating that although loss of VPS35 enhances IAV infection, it does so without affecting WASH complex integrity. Next, we depleted WASH1 in A549 cells and analyzed the localization of the WASH subunit CCDC53 and FAM21, together with VPS35, to assess complex integrity and WASH recruitment to VPS35. We overexpressed FAM21-GFP in WASH1 KD and control cells and performed IIF to detect endogenous CCDC53 and VPS35. By super-resolution structured illumination microscopy (SIM), we examined whether these factors colocalize under WASH1 deficiency conditions. We observed that CCDC53 levels were reduced in WASH1 KD cells compared to controls, accompanied by reduced co-localization with FAM21 and VPS35 (Fig. 4I). Interestingly, WASH1 deficiency did not affect FAM21 recruitment to VPS35, indicating that FAM21 can independently associate with VPS35 even when WASH complex integrity is compromised. Intrigued by this finding, we next addressed how the localization of other WASH components changes upon FAM21 silencing. To this end, we overexpressed GFP-WASH1 (mouse) in FAM21 KD cells, and by probing endogenous VPS35 and CCDC53, we observed colocalization of all three factors, suggesting that a fraction of WASH subunits can still be recruited to retromer-positive spots in a FAM21-independent manner. Strikingly, SIM imaging revealed an enrichment of GFP-WASH1 at the cell surface in FAM21-deficient cells, which was not observed in control cells (Fig. 4J). However, the CCDC53 signal was largely undetectable at the GFP-WASH1-enriched cell surface region, indicating that WASH1, while majorly existing in a complex, may also exhibit independent activity outside the complex.

Collectively, our findings reveal a critical role of WASH subunits and VPS35 in differentially regulating IAV entry. While WASH1, CCDC53, SWIP and Strumpellin promote viral entry, the FAM21-VPS35 axis exerts an antiviral effect. Our data further suggest that individual components of the WASH complex can function in a distinct manner, with important implications for the regulation of IAV entry.

### Retromer-uncoupled WASH promotes IAV endocytosis independently of its canonical NPF activity

**Figure 5:**
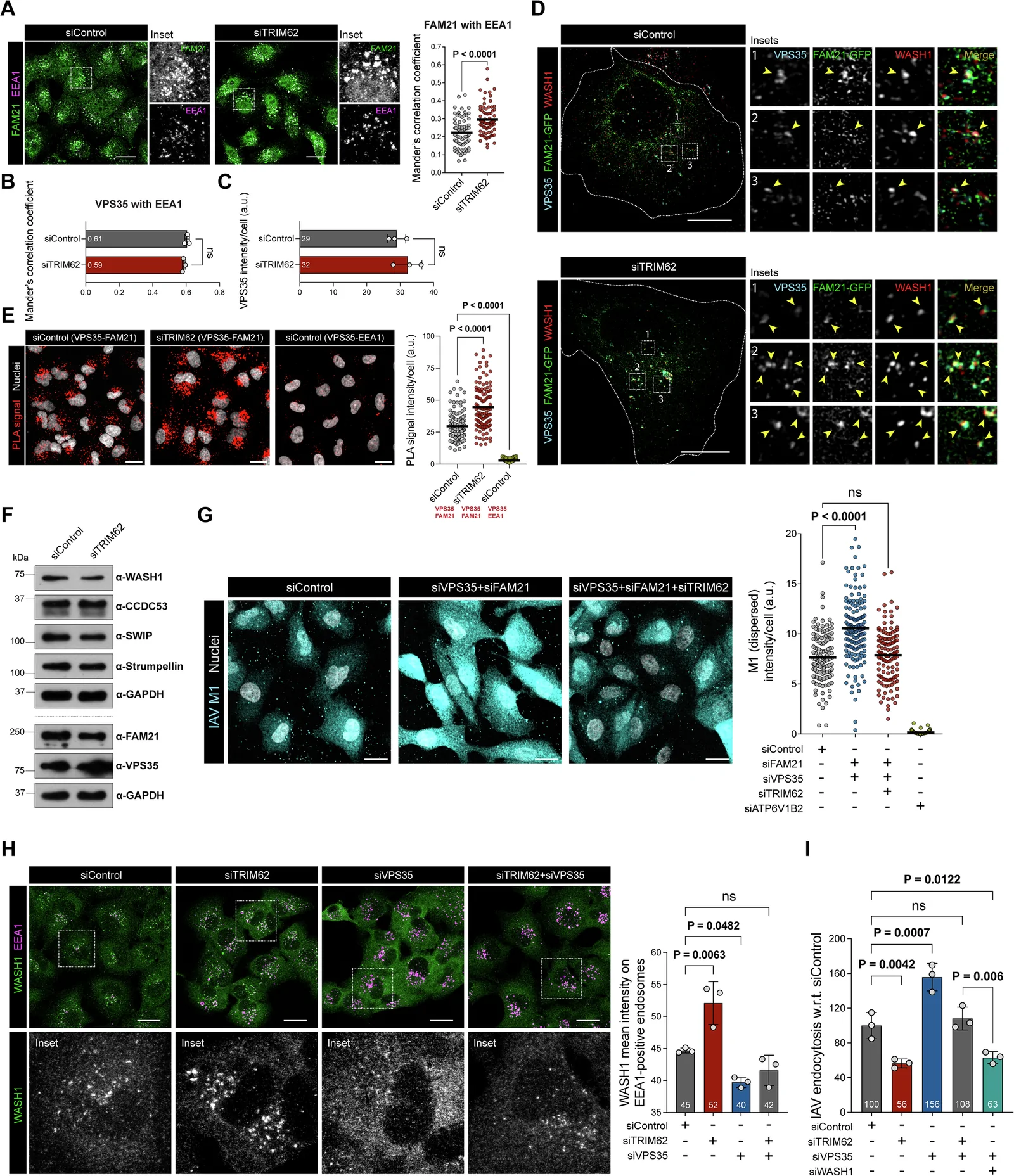
TRIM62 promotes WASH1-driven viral entry by regulating WASH-retromer association. (**A**) Confocal microscopy images (left) showing FAM21 (green) and EEA1-positive endosomes (magenta) in control and TRIM62 KD A549 cells. Insets show the distribution of FAM21 and EEA1. Scale bars, 20 µm. Quantification (right) of FAM21-EEA1 colocalization based on Mander’s correlation coefficient. *n* = 74 cells/siRNA (*N*  =  3 independent experiments; mean ± SD; error bars represent SD; unpaired two-tailed *t*-test; *P*  = 6.24 × 10⁻⁷). (**B**) Quantification of VPS35-EEA1 colocalization based on Mander’s correlation coefficient. *n* ≥ 1800 cells/siRNA (*N*  =  3 independent experiments; mean ± SD; error bars represent SD; unpaired two-tailed *t*-test; *P*  = 0.1055). Representative images are shown in Fig. EV4D. (**C**) Quantification of VPS35 intensity on EEA1-positive endosomes. *n* ≥ 800 cells/siRNA (*N*  =  3 independent experiments; mean ± SD; error bars represent SD; unpaired two-tailed *t*-test; *P*  = 0.3099). Representative images are shown in Fig. EV4D. (**D**) Super-resolution (SIM) images of control and TRIM62 KD A549 cell showing VPS35 (cyan), FAM21-GFP (green), and WASH1 (red). Cell boundaries are outlined. Insets show the distribution of FAM21-GFP, WASH1, and VPS35. Yellow arrowheads indicate colocalization. Scale bars, 10 µm. Representative images from three independent experiments are shown. (**E**) High-content confocal images (left) and quantification (right) of PLA signals (red) from FAM21-VPS35 and EEA1-VPS35 interactions. Scale bars, 20 µm. *n* = 118 cells/sample (*N*  =  3 independent experiments; mean ± SD; error bars represent SD; one-way ANOVA with Dunnett’s multiple-comparisons test). *P* values for each comparison: *P*  < 0.0001. (**F**) Western blots showing the levels of WASH complex proteins and VPS35 in control and TRIM62 KD A549 cells. The western blots are representative of three independent experiments. (**G**) High-content confocal images (left; IAV M1, cyan and nuclei, gray) and scatter plot (right) showing the quantification of cytosolic M1 (dispersed) intensity per cell. Scale bars, 20 µm. *n* ≥ 1500 cells/siRNA (*N*  =  3 independent experiments; mean ± SD; error bars represent SD; one-way ANOVA with Dunnett’s multiple-comparisons test). *P* values for siControl vs siVPS35+siFAM21: *P*  < 0.0001; siControl vs siVPS35+siFAM21+siTRIM62: *P*  = 0.8308. (**H**) Confocal microscopy images (left) showing WASH1 (green) and EEA1-positive endosomes (magenta) in control and TRIM62, VPS35 KD and TRIM62 + VPS35 double KD A549 cells. Lower panel shows zoomed-in images of WASH1. Scale bars, 20 µm. Quantification (right) of WASH1 intensity on EEA1-positive endosomes. *n* ≥ 70 cells/siRNA (*N*  =  3 independent experiments; mean ± SD; error bars represent SD; one-way ANOVA with Dunnett’s multiple-comparisons test). *P* values for siControl vs siTRIM62: *P*  = 0.0063; siControl vs siVPS35: *P*  = 0.0482; siControl vs siTRIM62+siVPS35: *P*  = 0.24. (**I**) Quantification of IAV endocytosis. *n* ≥ 1500 cells/siRNA (*N*  =  3 independent experiments; mean ± SD; error bars represent SD; one-way ANOVA with Dunnett’s multiple-comparisons test). *P* values for siControl vs siTRIM62: *P*  = 0.0042; siControl vs siVPS35: *P*  = 0.0007; siControl vs siTRIM62+siVPS35: *P*  = 0.8205; siControl vs siVPS35+siWASH1: *P*  = 0.0122; siTRIM62+siVPS35 vs siVPS35+siWASH1: *P*  = 0.006. Source data are available online for this figure.

**Figure EV4:**
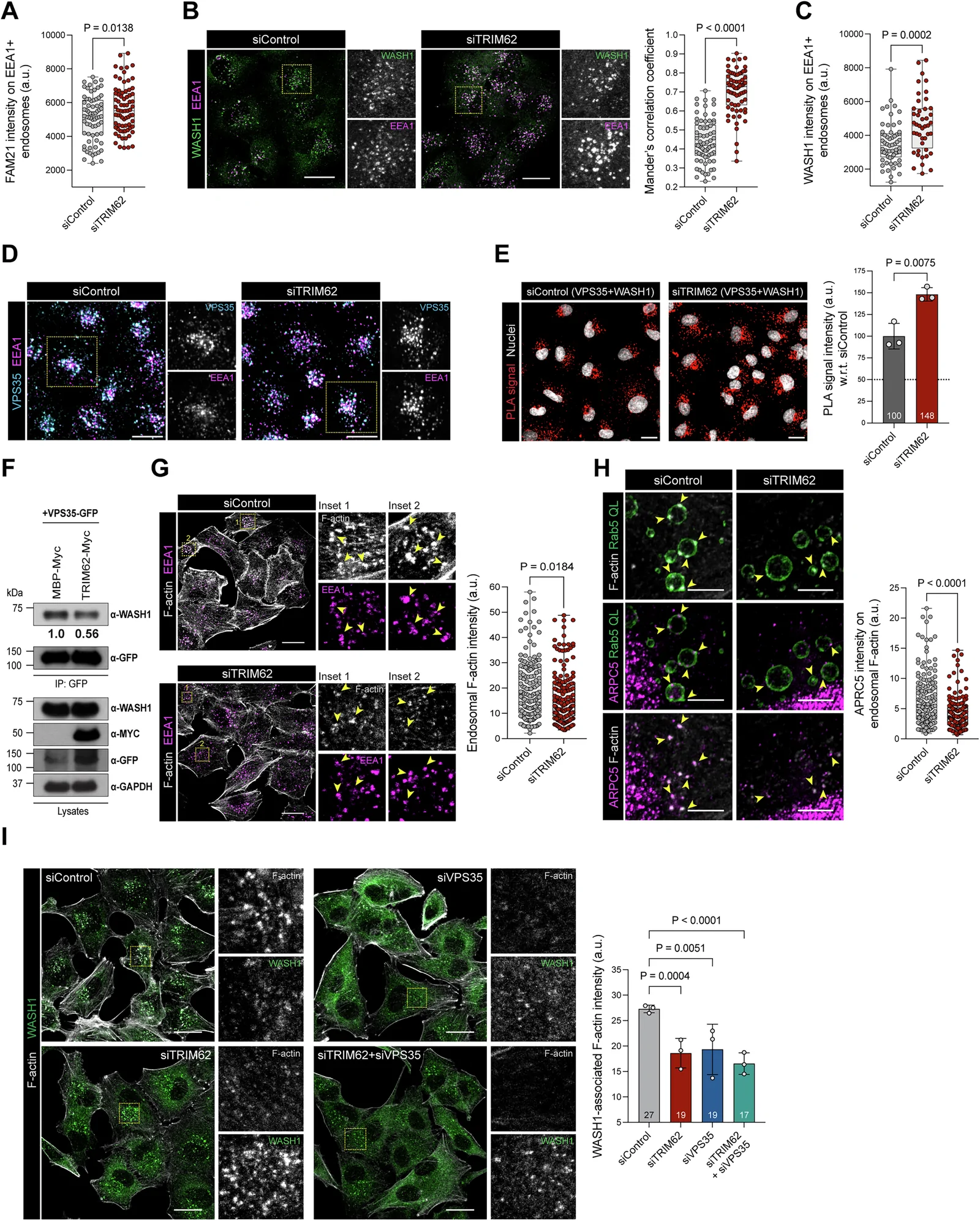
TRIM62 deficiency enhances endosomal recruitment of WASH but impairs its NPF activity. (**A**) Quantification of FAM21 intensity on EEA1-positive endosomes. *n* ≥ 45 cells/siRNA (*N*  =  3 independent experiments; mean ± SD; error bars represent SD; unpaired two-tailed *t*-test; *P*  = 0.0138). Representative images are shown in Fig. 5A. (**B**) Confocal microscopy images (left) showing WASH1 (green) and EEA1-positive endosomes (magenta) in control and TRIM62 KD A549 cells. Insets show the distribution of WASH1 and EEA1. Scale bars, 20 µm. Quantification (right) of WASH1-EEA1 colocalization based on Mander’s correlation coefficient. *n* = 70 cells/siRNA (*N*  =  3 independent experiments; mean ± SD; error bars represent SD; unpaired two-tailed *t*-test; *P*  = 2.46 × 10^−27^). (**C**) Quantification of WASH1 intensity on EEA1-positive endosomes. *n* ≥ 45 cells/siRNA (*N*  =  3 independent experiments; mean ± SD; error bars represent SD; unpaired two-tailed *t*-test; *P*  = 0.0002). (**D**) High-content confocal images showing VPS35 (cyan) and EEA1-positive endosomes (magenta) in control and TRIM62 KD A549 cells. Insets show the distribution of VPS35 and EEA1. Scale bars, 20 µm. For quantification, see Fig. 5B,C. (**E**) High-content confocal images (left) and quantification (right) of PLA signals from VPS35-WASH1 interaction. Scale bars, 20 µm. *n* ≥ 750 cells/sample (*N*  =  3 independent experiments; mean ± SD; error bars represent SD; unpaired two-tailed *t*-test; *P*  = 0.0075). (**F**) Pulldown assay showing interactions between VPS35-GFP and WASH1. HEK293T cells were co-transfected with VPS35-GFP and MBP-Myc or TRIM62-Myc, and lysates were incubated with GFP-Trap beads. Western blotting was performed to detect endogenous WASH1 in the precipitates. The western blots are representative of three independent experiments. (**G**) Confocal microscopy images (left) showing F-actin (gray) and EEA1-positive endosomes (magenta) in control and TRIM62 KD cells. Yellow arrowheads within the insets show the distribution of F-actin at endosomes. Scale bar, 20 µm. Scatter plot (right) showing F-actin intensity on EEA1-positive endosomes. *n* = 150 F-actin patches/siRNA (*N*  =  3 independent experiments; mean ± SD; error bars represent SD; unpaired two-tailed *t*-test; *P*  = 0.0184). (**H**) Confocal microscopy images (left) showing ARPC5 on GFP-Rab5QL-positive endosomes in control and TRIM62-depleted cells. Yellow arrowheads indicate F-actin and/or ARPC5 localization on enlarged, Rab5-QL-positive endosomes. Scale bars, 5 µm. Scatter plot (right) showing ARPC5 intensity within F-actin-positive regions on GFP-Rab5QL-positive endosomes. *n* = 150 F-actin patches/siRNA (*N*  =  3 independent experiments; mean ± SD; error bars represent SD; unpaired two-tailed *t*-test; *P*  = 1.68 × 10^−8^). (**I**) Confocal microscopy images (left) showing WASH1 (green) and F-actin (gray) in control, TRIM62, VPS35 KD, and TRIM62 + VPS35 double KD A549 cells. Insets showing zoomed-in images of F-actin patches on WASH1 spots. Scale bars, 20 µm. Quantification (right) showing WASH1-associated F-actin intensity. *n* ≥ 90 cells/siRNA (*N*  =  3 independent experiments; mean ± SD; error bars represent SD; one-way ANOVA with Dunnett’s multiple-comparisons test). *P* values for siControl vs siTRIM62: *P*  = 0.0004; siControl vs siVPS35: *P*  = 0.0051; siControl vs siTRIM62+siVPS35: *P*  < 0.0001. Data in (**A**–**C**, **G**, **H**) are represented as box and whiskers plots. The center line represents the median, the box boundaries correspond to the 25th and 75th percentiles (interquartile range), the whiskers extend to the minimum and maximum values, and all individual data points are overlaid.

Given the antiviral role of the FAM21-VPS35 axis, the proviral function of TRIM62 and other WASH subunits, and the observed colocalization of TRIM62 with WASH1 and FAM21, we sought to explore potential functional crosstalk between TRIM62 and WASH-VPS35 machinery. Therefore, we first examined if TRIM62 deficiency has any consequence on WASH distribution, majorly focusing on two key WASH components, FAM21 and WASH1. Since FAM21 function has been most extensively defined at endosomes, we examined FAM21 distribution on endosomal membrane upon TRIM62 depletion by confocal microscopy. Strikingly, TRIM62 KD in A549 cells led to a marked increase in the association of FAM21 with EEA1-positive early endosomes compared to control cells (Fig. 5A), accompanied by a significant increase in FAM21 intensity on endosomal compartments (Fig. EV4A). Similarly, WASH1 also showed enhanced association with early endosomes and increased intensity in TRIM62 KD cells (Fig. EV4B,C). In addition to early endosomes, we also examined the presence of WASH1 on Rab7-positive late endosomes in TRIM62 KD cells by high-resolution live-cell imaging. We co-expressed GFP-WASH1 (mouse) and red fluorescent protein (RFP)-tagged Rab7 (RFP-Rab7) in A549 cells and treated them with the PIKfyve inhibitor apilimod to induce endosomal enlargement (Uwada et al, 2025), thereby enabling clearer visualization of WASH subdomains on endosomes. We observed that compared to controls, TRIM62 depletion markedly enhanced WASH1 recruitment to Rab7-positive endocytic vesicles (Appendix Fig. S2A). To determine if the enhanced association of WASH with endosomes upon TRIM62 silencing resulted from increase in VPS35 accumulation on endosomes, thereby promoting WASH recruitment, we examined VPS35 association with early endosomes. We found that both VPS35 endosomal association and overall levels remained unchanged in TRIM62 KD cells (Figs. 5B,C and EV4D). These results suggested that TRIM62 specifically regulates endosomal WASH recruitment without altering VPS35 distribution or levels.

To further validate the observations from confocal microscopy, we examined the co-localization of FAM21 and WASH1 together with VPS35 using SIM. Consistent with the confocal microscopy observations, SIM imaging also revealed an enhanced association of both WASH1 and FAM21 with VPS35 (Fig. 5D). Since, WASH complex is primarily recruited to endosomal membranes via FAM21-VPS35 interaction, we examined whether the enhanced recruitment of WASH to endosomal subdomains following TRIM62 depletion resulted from increased interaction of FAM21 with VPS35. To this end, we examined WASH recruitment to retromer-enriched foci by assessing VPS35-FAM21 interaction using proximity ligation assay (PLA), which enables detection of proteins in close proximity (< 40 nm). Despite unchanged VPS35 levels (Fig. 5B,C), TRIM62-deficient A549 cells displayed significantly enhanced PLA signals (Fig. 5E), indicating enhanced association between VPS35 and FAM21. To verify assay specificity, we also checked PLA signals between EEA1 and VPS35, two proteins that reside on the same endosomal compartment but are not known to engage in direct molecular interactions. The near-background PLA signals observed for the VPS35-EEA1 pair confirmed the specificity of the assay (Fig. 5E). We next addressed whether the enhanced association of WASH with VPS35 is specific to A549 cells. To this end, we examined VPS35-FAM21 interaction in TRIM62-deficient HeLa cells and observed markedly elevated PLA signals, demonstrating that the effect is not cell-type specific (Appendix Fig. S2B). The specificity of PLA signals was validated by VPS35 depletion, which markedly reduced signal intensity, thereby confirming assay specificity (Appendix Fig. S2C). To determine whether TRIM62 depletion in A549 cells enhances the association of FAM21 alone or the WASH complex as a whole with VPS35, we performed PLA between WASH1 and VPS35 in TRIM62-deficient A549 cells. The increased WASH1-VPS35 PLA signals indicated enhanced recruitment of WASH to VPS35 (Fig. EV4E).

To complement our loss-of-function studies, we sought to determine whether overexpression of TRIM62 exerts a reciprocal effect and attenuates VPS35-WASH interaction. To this end, VPS35-GFP was overexpressed in the presence of TRIM62-Myc or MBP-Myc in HEK293T cells, and the interaction of VPS35 with endogenous WASH1 was examined by VPS35-GFP pulldown. Consistent with our PLA data, TRIM62-Myc overexpression reduced VPS35-WASH1 interaction by 44% relative to MBP-Myc overexpression (Fig. EV4F), suggesting that TRIM62 acts as a negative regulator of VPS35-WASH association. We next asked whether enhanced endosomal recruitment of WASH in TRIM62-deficient cells resulted from altered protein abundance, and therefore, we examined the levels of individual WASH subunits and VPS35 in TRIM62 KD or KO cells. However, we observed no appreciable differences in the protein levels of the WASH subunits or VPS35 (Fig. 5F; Appendix Fig. S2D), indicating that TRIM62 specifically regulates the subcellular localization of WASH, likely through modulating VPS35-WASH association.

The enhanced endosomal localization of WASH observed in TRIM62-deficient cells, together with the established role of WASH as an NPF, prompted us to examine branched F-actin assembly at endosomes. Unexpectedly, despite the increased WASH abundance on endosomes, F-actin patches associated with EEA1-positive early endosomes were significantly diminished in TRIM62-deficient cells compared with controls (Fig. EV4G). Since WASH promotes branched F-actin assembly on endosomes by activating the Arp2/3 complex, we next examined the endosomal localization of ARPC5, an Arp2/3 subunit. For this analysis, we utilized GFP-Rab5Q67L, a constitutively active GTP-bound Rab5 mutant that induces endosomal enlargement, thereby enabling improved spatial resolution for visualization of protein subdomains on endosomal membranes. We quantified the intensity of endogenous ARPC5 signals coinciding with F-actin patches and observed a significant reduction in their intensity in TRIM62-deficient cells relative to controls (Fig. EV4H). We next asked whether the impaired NPF activity of WASH observed in TRIM62-deficient cells translates into endosomal cargo sorting defects. To address this, we examined glucose transporter 1 (GLUT1), one of the prototypical cargoes of retromer/WASH. Previous studies have shown that VPS35 and FAM21 depletion leads to the mislocalization of GLUT1 from the plasma membrane to late endosomes/lysosomes or the Golgi, respectively (Evans et al, 2020; Lee et al, 2016). Notably, TRIM62 depletion in HeLa cells similarly caused steady-state accumulation of GLUT1 within the Golgi, as evidenced by its colocalization with the Golgi marker GM130 and further validated by brefeldin A- and monensin-induced Golgi perturbation (Appendix Fig. S2E). These findings indicate that despite enhanced recruitment of WASH to endosomes, WASH-dependent endosomal branched actin assembly and cargo sorting remain impaired in the absence of TRIM62, suggesting a previously undescribed role for TRIM62 as a regulator of WASH function.

Our finding that TRIM62 silencing reduces endosomal branched actin assembly raised the possibility that impaired NPF activity of WASH contributes to the IAV entry defects observed in TRIM62-deficient cells. However, this interpretation was difficult to reconcile with our earlier observation that global Arp2/3 inhibition had no significant effect on IAV entry (Fig. 3E). We therefore sought to determine whether the canonical NPF function of WASH is required for viral entry using an independent approach i.e., by silencing TRIM27, a factor with established role in activating WASH-mediated endosomal actin nucleation. TRIM27 specifically activates the WASH1 subunit through K63-linked ubiquitination, thereby relieving its autoinhibition and exposing its VCA domain, which in turn stimulates the Arp2/3 complex to drive actin nucleation at endosomes (Hao et al, 2013) (Appendix Fig. S2F). Although depletion of TRIM27 using a previously validated siRNA (Hao et al, 2013) expectedly reduced endosomal actin assembly (Appendix Fig. S2G), it had no measurable effect on IAV endocytosis (Appendix Fig. S2H). These findings suggest that the role of WASH in viral entry is unlikely to depend on its canonical NPF activity and instead may involve a mechanism that is largely separable from its established function in actin nucleation.

To understand the specific contribution of TRIM62 to viral entry in the context of WASH function, we next turned our attention to the antiviral VPS35-FAM21 axis. Given the enhanced VPS35-FAM21 association observed in TRIM62-deficient cells, we hypothesized that this alteration may be functionally linked to the impaired viral entry phenotypes and that TRIM62 may promote entry by limiting this association. To test this, we performed co-depletion experiments and monitored IAV uncoating as a readout of viral entry. Co-depletion of VPS35 and FAM21 markedly increased IAV uncoating, which was restored to control levels by additional TRIM62 silencing (Fig. 5G), These genetic perturbation experiments indicate that TRIM62 acts upstream of this regulatory node, further indicating its crucial role in fine-tuning VPS35-FAM21 association, thereby supporting viral entry.

Given the contrasting roles of the non-WASH factors TRIM62 and VPS35 in viral entry, we next sought to determine if they differentially regulate WASH subcellular distribution and thereby exert opposing effects on viral entry. To this end, we silenced TRIM62 and VPS35 individually or in combination, and examined WASH1 distribution on EEA1-positive early endosomes. Additionally, we also quantified IAV endocytic uptake in similar genetic perturbation experiments. We found that TRIM62 silencing alone increased endosomal WASH1 and suppressed viral endocytosis, whereas VPS35 loss redistributed WASH1 to the cytosol and enhanced viral uptake (Fig. 5H,I), highlighting reciprocal functions of these factors. Notably, enhanced endosomal WASH1 localization correlated with reduced viral entry, whereas increased cytosol WASH1 (non-endosomal pool) correlated with more efficient viral entry. Co-depletion of TRIM62 and VPS35 counterbalanced their opposing effects, restoring WASH1 distribution to that observed in control cells and rescuing viral endocytosis to basal levels (Fig. 5H,I), suggesting that TRIM62 and VPS35 act antagonistically to regulate WASH1 localization without altering its levels and that WASH1 localization in turn determines viral entry efficiency. Given our earlier findings that WASH1 itself is a determinant of viral entry (Fig. 4), we hypothesized that silencing WASH1 in VPS35 depleted cells would impair viral entry. We co-depleted WASH1 and VPS35 and verified their KD by IIF, followed by quantitative analysis, which showed robust reductions of both proteins in co-depleted cells (Appendix Fig. S3). We observed that indeed, co-depletion of WASH1 and VPS35 significantly reduced IAV endocytosis (Fig. 5I), underscoring the criticality of WASH1 in this process. In cells co-depleted of TRIM62 and VPS35, where IAV internalization was restored, endosomal actin still remained reduced (EV4I), further confirming that WASH supports IAV entry independently of its NPF activity.

Collectively, our data suggest that WASH1 can support IAV entry even when its NPF activity is compromised. Furthermore, we establish that while the presence of WASH1 is a prerequisite for successful viral entry, its subcellular localization, oppositely regulated by TRIM62 and VPS35, also critically determines viral entry efficiency.

### TRIM62 interacts with FAM21 and regulates WASH-retromer association

**Figure 6:**
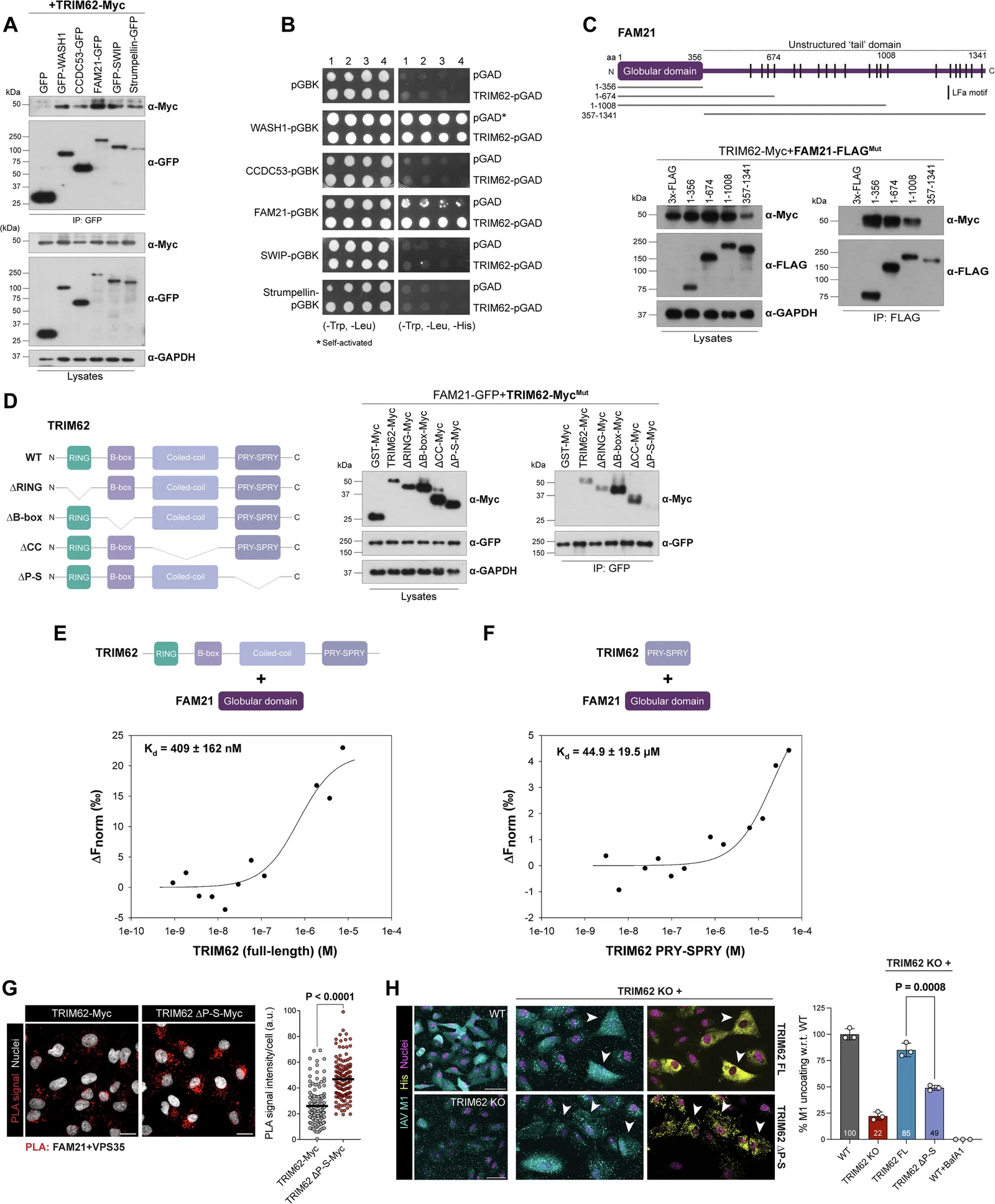
The PRY-SPRY domain of TRIM62 mediates interaction with FAM21 and plays a critical role in IAV entry. (**A**) Pulldown assay showing interactions between TRIM62 and WASH subunits. HEK293T cells were co-transfected with TRIM62-Myc and GFP-tagged constructs of WASH subunits including WASH1, CCDC53, FAM21, SWIP and Strumpellin, and lysates were incubated with GFP-Trap beads. Western blotting was performed to detect TRIM62-Myc in the precipitates. The western blots are representative of three independent experiments. (**B**) Y2H assay showing a direct interaction between TRIM62 and FAM21. Yeast co-transformants were spotted on -Trp, -Leu plates (left) to indicate growth, and -Trp, -Leu, -His plates (right) to confirm interaction. * Indicates self-activation for WASH1. Representative images from three independent experiments are shown. (**C**) Schematic showing FAM21 domain organization (top). Pulldown assay (bottom) showing interactions between TRIM62 and FAM21 truncation mutants. HEK293T cells were co-transfected with TRIM62-Myc and FAM21-FLAG mutant constructs, and lysates were incubated with FLAG-immunotrap beads. Western blotting was performed to detect TRIM62-Myc in the precipitates. The western blots are representative of three independent experiments. (**D**) Schematic showing TRIM62 domain organization and mutants (left). Pulldown assay (right) showing interactions between FAM21 and TRIM62 domain-deletion mutants. HEK293T cells were co-transfected with FAM21-GFP and TRIM62-Myc mutant constructs, and lysates were incubated with GFP-Trap beads. Western blotting was performed to detect TRIM62-Myc in the precipitates. The western blots are representative of three independent experiments. (**E**, **F**) MST binding curves showing interaction of labeled FAM21 (1–356 aa) with TRIM62-FL (**E**) and TRIM62 P-S (**F**). Each data point represents experimental measures as mean ± SEM from three independent experiments and solid line indicates best-fit binding curves. (**G**) High-content confocal images (left) and quantification (right) of PLA signals (red) from VPS35-FAM21 interaction in cells stably expressing either TRIM62-Myc or TRIM62 ∆P-S-Myc. Scale bars, 20 µm. *n* = 130 cells/sample (*N*  =  3 independent experiments; mean ± SD; error bars represent SD; unpaired two-tailed *t*-test; *P*  = 1.45 × 10^−24^). (**H**) High-content confocal images (left) showing M1 uncoating in WT, TRIM62 KO, and KO cells stably expressing WT TRIM62-His or its mutants. M1 (cyan), His (yellow), and nuclei (magenta). White arrowheads indicate uncoating in His-positive cells. Scale bars, 50 µm. Quantification of % M1 uncoating, normalized to WT. *n* ≥ 450 cells/sample (*N*  =  3 independent experiments; mean ± SD; error bars represent SD; unpaired two-tailed *t*-test). *P* value for TRIM62 FL vs TRIM62 ∆P-S: *P*  = 0.0008. Source data are available online for this figure.

**Figure EV5:**
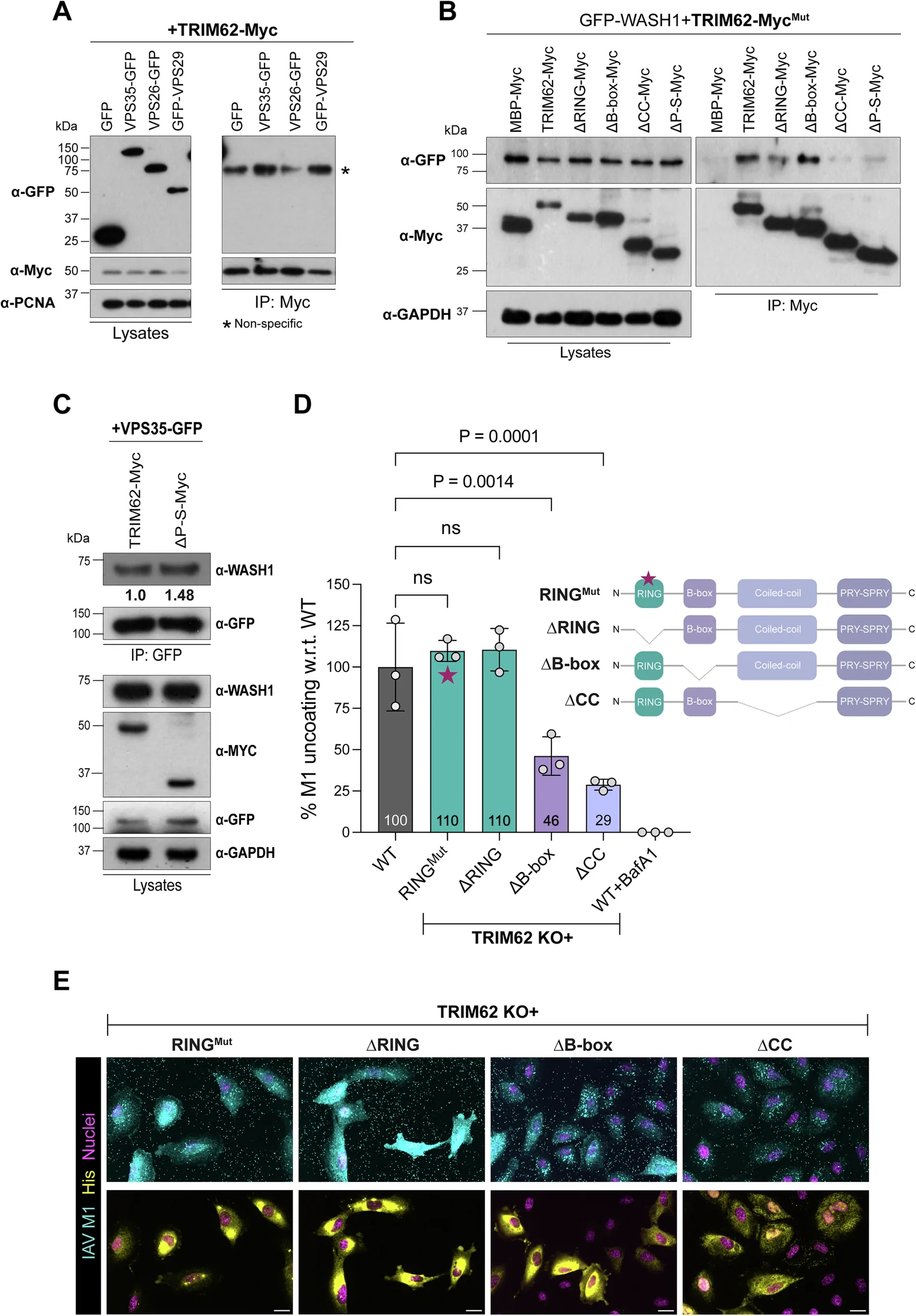
TRIM62 interacts with WASH via its C-terminal region and promotes IAV entry independent of its E3 ubiquitin ligase activity. (**A**) Pulldown assay examining interactions between TRIM62 and retromer subunits: VPS35, VPS26, and VPS29. HEK293T cells were transfected with TRIM62-Myc and GFP-tagged retromer subunits and lysates were incubated with Myc-Trap beads. Western blotting was performed to detect GFP-tagged retromer subunits in the precipitates. PCNA was used as a loading control, and asterisk (*) indicates non-specific bands. (**B**) Pulldown assay showing interactions between WASH1 and TRIM62 truncation mutants. HEK293T cells were co-transfected with GFP-WASH1 and TRIM62-Myc mutant constructs, and lysates were incubated with Myc-Trap beads. Western blotting was performed to detect GFP-WASH1 in the precipitates. (**C**) Pulldown assay showing interactions between VPS35-GFP and WASH1. HEK293T cells were co-transfected with VPS35-GFP and TRIM62-Myc or TRIM62 ΔP-S-Myc, and lysates were incubated with GFP-Trap beads. Western blotting was performed to detect endogenous WASH1 in the precipitates. (**D**) Quantification (left) of % M1 uncoating normalized to WT. *n* ≥ 450 cells/sample (*N*  =  3 independent experiments; mean ± SD; error bars represent SD; one-way ANOVA with Dunnett’s multiple-comparisons test). *P* values for WT vs RING^Mut^: *P*  = 0.8337; WT vs ΔRING: *P*  = 0.7950; WT vs ΔB-box: *P*  = 0.0014; WT vs ΔCC: *P*  = 0.0001. Schematic showing TRIM62 domain organization (right). (**E**) High-content confocal images for Fig. EV5D showing M1 uncoating in WT, TRIM62 KO, and KO cells stably expressing His-tagged TRIM62 mutants. M1 (cyan), His (yellow), and nuclei (magenta). Scale bars, 20 µm. All western blots are representative of three independent experiments.

Based on our observations that TRIM62 regulates WASH accumulation on endosomes via limiting VPS35-FAM21 association and it co-localizes with WASH at the leading edge of cells, we sought to examine whether TRIM62 exerts its regulatory activity by physically interacting with WASH. To assess interactions between TRIM62 and WASH subunits, we performed pulldown assays using GFP-nanotrap immunocapture on extracts from HEK293T cells that co-expressed TRIM62-Myc and GFP-tagged WASH subunits. Interestingly, we detected interaction between TRIM62 and all WASH subunits (Fig. 6A). Given the increased association between VPS35 and FAM21 in TRIM62-deficient cells, we also examined whether TRIM62 interacts with VPS35 or the other core retromer subunits, VPS26 or VPS29. However, no interaction was detected with VPS35, VPS26, or VPS29 (Fig. EV5A), highlighting the specificity of TRIM62 for WASH. Next, we examined whether TRIM62 directly interacts with WASH subunits by yeast two-hybrid (Y2H) assay. Since yeast lacks conserved WASH components (Derivery and Gautreau, 2010; Seaman, 2012), this system was chosen to detect direct interactions. Among WASH subunits, yeast growth was observed in the presence of FAM21, despite some degree of self-activation, indicating a direct interaction with TRIM62. However, strong self-activation of WASH1 in yeast precluded reliable determination of its direct interaction with TRIM62 (Fig. 6B).

After identifying a direct interaction of TRIM62 with FAM21, we next aimed to map the domains responsible for this interaction. Using FAM21 truncation mutants, we found that the N-terminal globular region (1–356 aa) of FAM21, which includes the “head” domain (1–200 aa), mediates interaction with TRIM62 (Fig. 6C). This “head” domain of FAM21 is essential for its incorporation into the WASH complex (Guo et al, 2024; Jia et al, 2010; Harbour et al, 2012), while its ~1100 amino acid-long disordered “tail” region, which harbors multiple leucine-phenylalanine-acidic (LFa) motifs, mediates endosomal anchoring via interaction with VPS35 (Gomez and Billadeau, 2009; Jia et al, 2010; Helfer et al, 2013). Co-immunoprecipitation assays with TRIM62 truncation mutants revealed that the N-terminal globular region of FAM21 interacts specifically with the C-terminal P-S domain of TRIM62. Loss of the P-S domain abolished FAM21 interaction, whereas mutants lacking the RING, B-box, or CC domains did not (Fig. 6D). In addition to mediating interaction with FAM21, the P-S domain also contributes to WASH1 binding, as evidenced by co-immunoprecipitation analysis. The CC domain of TRIM62 was also found to contribute to WASH1 interaction (Fig. EV5B).

After identifying the N-terminal globular domain of FAM21 as critical for mediating the interaction with TRIM62, we sought to further validate this interaction using purified proteins. To this end, we purified the FAM21 globular domain (1–356 aa), full-length (FL) TRIM62 (TRIM62 FL), and the P-S domain of TRIM62 (TRIM62 P-S), (Appendix Fig. S4A–C), and performed MicroScale Thermophoresis (MST). FAM21 (1–356 aa), was labeled with RED-NHS, and its interaction was monitored at varying concentrations of each of the analytes. The obtained dose–response curves were best fitted to one-site binding model, with dissociation constants (K_D_) of 0.40 μM and 44.9 μM for TRIM62 FL and the TRIM62 P-S domain, respectively (Fig. 6E,F). MST analyses indicated that while FAM21 globular domain directly interacts with the TRIM62 P-S domain, it binds TRIM62 FL with higher affinity.

Having established a direct interaction of TRIM62 (via its P-S domain) with FAM21, we next sought to examine the functional relevance of this domain in regulating VPS35-FAM21 association and IAV entry. We generated A549 cells stably expressing either TRIM62-Myc or a P-S domain truncation mutant (TRIM62 △P-S-Myc), and examined VPS35-FAM21 association by PLA. Cells expressing TRIM62 △P-S-Myc displayed significantly enhanced PLA signals relative to TRIM62-Myc (Fig. 6G), indicating that the P-S domain negatively regulates this association, with possible consequences on WASH recruitment. To further delineate the function of the P-S domain in VPS35-WASH association, we performed pulldown assays using GFP-nanotrap immunocapture on extracts from HEK293T cells co-expressing VPS35-GFP together with either TRIM62-Myc (FL) or the TRIM62 ΔP-S-Myc mutant, and analyzed co-precipitation of endogenous WASH1. Consistent with our PLA data showing enhanced VPS35-FAM21 association, VPS35- WASH1 interaction was increased by 48% in the presence of TRIM62$\,\Delta$P-S-Myc, compared with TRIM62-Myc (FL) (Fig. EV5C), suggesting a critical role for the P-S domain in the regulation of VPS35-WASH association. Finally, we investigated the functional relevance of the P-S domain in IAV entry. To this end, we performed rescue experiments in TRIM62 KO cells stably expressing either TRIM62 FL or the TRIM62 ΔP-S mutant. While re-expression of TRIM62 FL in the KO background almost fully rescued the M1 uncoating defect observed in the KO cells, TRIM62$\,\Delta$P-S failed to restore it (Fig. 6H), underscoring the importance of the P-S domain in viral entry. Importantly, we also found that the RING mutant lacking E3 ubiquitin ligase activity (RING^Mut^) and the RING domain deletion mutant (ΔRING) fully rescued uncoating in KO cells, whereas mutants lacking the B-box (ΔB-box) or CC (ΔCC) domains failed to do so (Fig. EV5D,E). Although the present study focused on delineating the role of the TRIM62 P-S domain in regulating VPS35-WASH association and its impact on IAV entry, the contribution of other TRIM62 domains in viral entry remains to be explored.

Taken together, these findings implicate TRIM62 as a key regulator of WASH-retromer association, likely through its interaction with FAM21 via the P-S domain. Our data support a model in which TRIM62 limits WASH-retromer association, thereby maintaining a retromer-free pool of WASH that promotes efficient IAV entry.

## Discussion

Endocytosis provides key opportunities for diverse viruses to internalize into host cells, allowing them to bypass cortical barriers, navigate cytosolic crowding, and move deep into the cytoplasm where they can release their genomes for replication (Sempere Borau and Stertz, 2021; Marsh and Helenius, 2006). Multiple endocytic processes operate at the eukaryotic plasma membrane, enabling viruses to utilize a preferred pathway or co-opt parallel entry routes for efficient internalization and establishment of infection. These coexistent pathways are subject to stringent regulation, thereby preventing uncontrolled pathogen invasions. For IAV, a pathogen responsible for some of the deadliest pandemics in human history and a continuing threat, the mechanisms of host cell entry remain incompletely understood. Although IAV predominantly relies on CME for cellular entry, a previous study reported that co-suppression of CME and the caveolar pathway in HeLa cells does not prevent IAV infection (Sieczkarski and Whittaker, 2002). A subsequent study reported that silencing Epsin 1, a CME-specific adapter essential for IAV endocytosis, does not reduce viral internalization (Chen and Zhuang, 2008), revealing a remarkable capacity of IAV to readily switch from clathrin-dependent to clathrin-independent endocytic pathways when CME is compromised. These observations underscore the significant contribution of clathrin-independent pathways to IAV entry.

In the cellular entry program of IAV, this study identifies WASH as a previously unrecognized regulator of viral internalization and endosomal trafficking. We find that WASH acts as a dual regulatory hub during viral entry, with WASH1, CCDC53, SWIP, and Strumpellin facilitating clathrin-independent viral uptake, whereas the FAM21 subunit acts antagonistically to restrict this process. We further find that WASH-dependent viral entry is restrained by FAM21 through its coupling of WASH to the retromer subunit VPS35, which spatially restricts WASH from supporting viral endocytic uptake. Here, we show that a clathrin-independent, lipid raft pathway utilized by CTxB for internalization is also co-opted by IAV and is supported by both WASH1 and TRIM62, revealing a functional convergence of these factors in promoting endocytic uptake through this route. We observed IAV binding to CTxB-positive lipid raft domains at the plasma membrane and colocalization of IAV with CTxB during endocytic uptake, consistent with previous findings implicating lipid rafts in IAV entry (Verma et al, 2018; Li et al, 2022). Interestingly, we also found colocalization of WASH1 with CTxB both at the cell surface and during its internalization, as well as with IAV during endocytic uptake, implicating WASH1 in the lipid raft-dependent entry pathway co-opted by IAV. In addition to WASH1, we find that other WASH subunits including CCDC53, SWIP and Strumpellin also support viral endocytosis, whereas FAM21 exerts an antiviral function despite being an integral component of the same complex, uncovering a striking functional dichotomy with the WASH machinery. Although FAM21 has been reported to be crucial for maintaining WASH1 levels and WASH complex stability in mammalian cells (Gomez and Billadeau, 2009; Romano-Moreno et al, 2024; Visweshwaran et al, 2018), the complex was found to remain intact without FAM21 in *Dictyostelium* (Park et al, 2013), indicating that the requirement for FAM21 in WASH stability is context-dependent rather than universal, reflecting evolutionary and regulatory differences across organisms. This functional antagonism within the complex points to a role for FAM21 as a context-dependent regulatory element, highlighting its divergent behavior relative to the other WASH subunits. Notably, studies on VV entry reported that the WASH subunit FAM21 colocalizes with CTxB and VV at the HeLa cell plasma membrane and that WASH1 and FAM21, but not other WASH subunits, are required for VV entry (Huang et al, 2008; Hsiao et al, 2015).

A recent study described a clathrin-independent endocytic pathway for HPV16, driven by WASH1 through its NPF activity (Brinkert et al, 2025). In this study, the authors found the involvement of WASH1 in the late stage of viral endocytosis, where it promotes endocytic vesicle scission via F-actin polymerization. Although both this study and ours highlight a critical role for WASH1 in HPV16 and IAV endocytosis, respectively, the SHRC subunits were found to exhibit divergent functions. Contrasting to our findings on IAV entry, CCDC53, SWIP, and Strumpellin showed no role in HPV16 entry, while FAM21 had only a modest effect (Brinkert et al, 2025). Similar results were obtained for CTxB uptake in A549 cells, where depletion of individual SHRC subunits did not significantly impair internalization. At first glance, this may seem unexpected, as loss of SHRC components would be anticipated to destabilize the WASH complex and compromise its function. However, depletion of individual SHRC subunits only partially reduced WASH1 levels, indicating that a residual pool of WASH1 still remained. This residual WASH1 may retain sufficient functional activity, which could explain why SHRC depletion does not fully phenocopy WASH1 depletion. Consistent with this idea, knockdown of WASH1 and individual SHRC components produced distinct effects on both IAV and CTxB uptake. Together, these findings suggest that the consequences of SHRC perturbation extend beyond simply reducing WASH1 abundance and point to additional cargo-specific roles for SHRC components in regulating endocytic trafficking.

Given the distinct trafficking routes of IAV and HPV16, where IAV follows the degradative pathway to reach late endosomes, whereas HPV16 traffics to the *trans*-Golgi without involving major endocytic regulators such as clathrin, caveolin, dynamin, or cholesterol, we reason that the differential involvement of WASH subunits in their entry is due to differences in the endocytic machinery exploited by these fundamentally distinct viruses. Another key difference between WASH function in HPV16 and IAV entry lies in its NPF activity: while HPV16 relies on WASH-mediated branched actin nucleation at the plasma membrane for vesicle scission, we found that IAV endocytosis in A549 cells occurs independently of branched actin, yet requires WASH. The non-essentiality of WASH NPF activity in IAV entry is supported by the observation that depletion of TRIM27, an essential activator of WASH, does not impair viral endocytosis. Consistent with previous findings that TRIM27 silencing fails to activate WASH for its NPF activity (Hao et al, 2013), we also observed markedly reduced dendritic actin networks at endosomes upon TRIM27 depletion, yet viral internalization remained unaffected. These findings highlight a non-canonical, actin-independent role for WASH in IAV endocytosis and point to a potential scaffolding function in endocytic processes, raising the possibility that WASH1 may directly contribute to viral uptake. Although we observed strong colocalization of WASH1 with IAV during early entry, the present study did not investigate whether WASH1 directly drives viral internalization. While WASH1 enrichment was detected at the plasma membrane in FAM21-depleted cells, the mechanism by which WASH1 associates with the plasma membrane in the absence of an intact complex remains to be determined. This question was not addressed in the present study, and whether WASH1 can autonomously mediate IAV endocytosis remains to be explored.

The present study further investigated how WASH1 activity, along with that of other WASH subunits with proviral functions, is antagonized by FAM21. Since FAM21 directly interacts with VPS35, linking WASH to retromer, we interrogated the role of VPS35 in IAV entry and found its restrictive function similar to FAM21. The functional significance and underlying mechanisms that distinguish FAM21 from the rest of the WASH subunits remain poorly understood. In the context of IAV entry, we find that FAM21, together with VPS35, negatively regulates viral entry by constraining WASH mobilization to endocytic sites for viral internalization. This may explain why FAM21 exerts a distinct function in IAV entry relative to other WASH subunits, as our study shows that the FAM21-VPS35 interaction, which keeps WASH coupled to retromer, limits the availability of free pool of WASH required for viral uptake. Our findings also suggest that the inhibitory FAM21-VPS35 axis is antagonized by TRIM62, which reduces WASH-retromer association and promotes IAV entry. This TRIM62-mediated antagonism is supported by the observations that TRIM62 silencing enhances WASH-retromer association, whereas its overexpression reduces it, leading to decreased or increased viral internalization, respectively. Interestingly, we observe that VPS35 depletion produces the opposite phenotypes, underscoring the reciprocal roles of TRIM62 and VPS35 in regulating WASH localization and viral entry. Furthermore, we find that TRIM62 selectively interacts with all WASH components, but not with retromer subunits, and directly binds FAM21. Based on these observations, we propose a model in which TRIM62 limits WASH-retromer association through its interaction with FAM21, thereby increasing the availability of retromer-free WASH to support viral endocytosis.

Although our data collectively support the proposed model, several limitations and unresolved mechanistic questions warrant consideration. First, our pulldown experiments demonstrated that TRIM62 associates with all WASH subunits, and subsequent Y2H assay and MST showed a direct interaction with FAM21, suggesting that TRIM62 associates with WASH through FAM21. However, we found that this interaction is mediated by the N-terminal globular region (1–356 aa) of FAM21, which encompasses the “head” domain (1–200 aa) responsible for its incorporation into WASH through interactions with WASH1 and Strumpellin (Guo et al, 2024; Jia et al, 2010; Harbour et al, 2012). If TRIM62 associates with the intact WASH, even transiently, it remains unclear how it interacts with the N-terminal globular region of FAM21, given that the “head” domain remains buried within the assembled complex. One possibility is that the residues in the globular region that lie beyond the 200 aa-long “head” domain mediate this interaction. Alternatively, since the structure of the FAM21 globular region within the assembled WASH complex has not yet been resolved, it remains possible that surface-exposed elements of the globular region are accessible to TRIM62, providing an interface for its binding. Future biochemical and structural studies will be required to resolve the TRIM62-FAM21 binding interface and determine whether TRIM62 binds FAM21 within the assembled complex or during transient stages of complex assembly. Second, the mechanism by which TRIM62-FAM21 interaction antagonizes WASH recruitment to retromer via VPS35 remains unclear. The interaction between FAM21 and VPS35 is mediated by the extended disordered “tail” of FAM21, whereas our data indicate that TRIM62 binds the N-terminal globular region of FAM21, making it unlikely that TRIM62 directly competes with VPS35 for FAM21 binding. One possibility is that TRIM62 induces allosteric or conformational changes within the complex that reduce the accessibility or affinity of the FAM21 tail for VPS35. Resolving this mechanism will require biochemical and high-resolution structural analyses. Third, we observed in MST analyses that full-length TRIM62 binds the FAM21 globular region with high (nanomolar) affinity, whereas its isolated P-S domain binds the same region with relatively lower (micromolar) affinity. Given our pulldown data indicating that the P-S domain is critical for this interaction, it remains unclear why the isolated P-S domain binds FAM21 with only low affinity in MST analyses. This observation raises the possibility that although the P-S domain provides the primary binding interface for FAM21, additional regions of TRIM62 contribute to stabilizing the interaction and enhancing its overall affinity. Future studies with systematic domain deletion mutants, coupled with quantitative biophysical measurements of binding affinity, will be required to define the contribution of individual domains and determine whether they act corporately to achieve high-affinity binding to FAM21. Fourth, although endogenous WASH1 was detected in VPS35 pulldown samples, endogenous FAM21 could not be detected under the conditions used in this study. Given that the FAM21-VPS35 interaction is highly sensitive to biochemical conditions used, further optimization of the pulldown assay will be needed to reliably capture this interaction. Fifth, despite identifying the proviral roles of the WASH subunits WASH1, CCDC53, SWIP, and Strumpellin, the mechanistic basis of their contributions to IAV endocytosis and trafficking remains largely unclear. Whether these subunits act cooperatively within the assembled WASH complex during viral entry or exert distinct functions outside the complex to support endocytic trafficking remains to be determined.

While our findings implicate all WASH subunits, VPS35, and TRIM62 in IAV entry, we cannot exclude the possibility that their depletion elicits broader cellular perturbations that contribute to the observed entry phenotypes. However, previous studies showing that WASH deficiency does not affect EGF or Tfn uptake (Gomez and Billadeau, 2009; Duleh and Welch, 2010), together with our observations that TRIM62 silencing likewise does not impair these uptake pathways, suggest that despite potential perturbations to the cellular trafficking landscape, the endocytic pathways utilized by these cargoes remain largely unaffected. In contrast, we found that both IAV and CTxB uptake were impaired in cells depleted of either WASH1 or TRIM62, suggesting a specific role for these factors in lipid raft-mediated endocytic uptake and highlighting a functional intersection between them. In this study, we establish that WASH coupling to retromer via FAM21-VPS35 interaction has important functional consequence for IAV entry. However, the possibility remains that additional factors involved in the endosomal sorting and recycling machinery are involved in regulating viral entry. For example, we found that VPS35 depletion enhances viral entry, likely by liberating WASH from retromer-mediated sequestration, thereby increasing its availability to support viral endocytosis. However silencing this key retromer subunit may have broader consequences, given the established role of retromer in preventing lysosomal degradation of several transmembrane receptors, a function that is critical for preserving their homeostasis (Temkin et al, 2011; Hesketh et al, 2014). Retromer subunits lack intrinsic lipid-binding domains and therefore, retromer relies on the small GTPase Rab7, in its active GTP-bound form, for endosomal recruitment (Seaman et al, 2009; Rojas et al, 2008; Harrison et al, 2014). While dominant-negative Rab7 (T22N) has been reported to impair IAV entry (Sieczkarski and Whittaker, 2003), the mechanisms underlying Rab7-dependent viral trafficking and its intersection with VPS35-mediated retromer activity remain to be elucidated. Following recruitment, retromer is uncoated from the endosomal membrane by the activity of TBC1D5, a GTPase-activating protein (GAP) for Rab7 (Seaman et al, 2009; Jia et al, 2016). Interestingly, a recent study demonstrated that autophagy induced by nutrient deprivation or mTOR inhibition leads to the capture of TBC1D5-retromer complex by phagophores, resulting in bulk degradation of TBC1D5-positive endosomes and consequent suppression of receptor recycling (Carosi et al, 2024). Together, these observations raise the possibility that IAV entry is governed by a broader regulatory network. Therefore, future studies will be required to elucidate how retromer dynamics, Rab7 activity, and autophagy converge to influence viral entry.

Taken together, our data support a model in which WASH acts as a key determinant of IAV endocytosis, with its partitioning between endosomal membranes and the cytosol exerting opposing effects on viral entry. Our findings suggest that while retromer coupling biases WASH toward its established role in endosomal cargo recycling, dissociation from retromer increases the availability of WASH to facilitate viral internalization. Within this framework, TRIM62 antagonizes WASH-retromer association and promotes viral entry. Our data also suggest that WASH supports IAV endocytosis independently of its branched actin-nucleation activity, likely through a non-canonical scaffolding mechanism. However, the precise molecular basis of this activity remains unresolved and will require further investigation. Finally, the WASH-dependent mechanisms described here likely represent only part of a broader host regulatory network governing viral entry, and identifying additional components of this network and defining their mechanisms of action will be important objectives for future studies.

## Methods

**Table:** Reagents and tools table

Reagent/Resource	Reference or Source	Identifier or Catalog Number
**Experimental models**		
A549	ATCC	CCL-185
MDCK-II	ATCC	CRL-2936
HeLa	ATCC	CCL-2
HepG2	ATCC	HB-8065
Huh-7	Merck	01042712
HEK293T	ATCC	CRL-3216
IAV X-31 (H3N2)	Microbiologics (formerly Virapur)	NA
IAV UDORN (H3N2)	Yohei Yamauchi, ETH Zurich	A/UDORN/72
IAV NYMC_X311 (H3N2)	Yohei Yamauchi, ETH Zurich	A/Brisbane/1/2018 x A/PR/8/34
IAV WSN (H1N1)	Yohei Yamauchi, ETH Zurich	A/WSN/1933
Semliki forest virus (SFV)	Yohei Yamauchi, ETH Zurich	SFV-ZsG-GFP
Vaccinia virus (VV)	Yohei Yamauchi, ETH Zurich	VV-GFP
Vesicular stomatitis virus (VSV)	Dr. Santosh Chauhan, CCMB, Hyderabad	VSV-GFP
**Recombinant DNA**		
Plasmids		See Appendix Table S1
**Antibodies**		
Mouse anti-IAV-NP (HB65)	ATCC	H16-L10-4R5
Rabbit anti-GAPDH	Cell Signalling Technology	2118s
Mouse anti-IAV-M1 (HB64)	ATCC	M2-1C6-4R3
Mouse anti-His tag	Santa Cruz Biotechnology	sc-53073
Rabbit anti-IAV-HA (PINDA)	Yohei Yamauchi, ETH Zurich	NA
Mouse anti-IAV-HA (H3SKE)	Yohei Yamauchi, ETH Zurich	NA
Mouse anti-IAV-HA (A1)	Yohei Yamauchi, ETH Zurich	NA
Rabbit anti-His tag	Cell Signalling Technology	12698s
Rat anti-Myc tag	ChromoTek	9E1
Rabbit anti-GFP	Abgenex	Purified
Rabbit anti-EEA1	Cell Signalling Technology	3288s
Mouse anti-EEA1	BD Biosciences	610457
Rabbit anti-FAM21	Invitrogen	PA5-35883
Rabbit anti-WASH1	Invitrogen	PA5-51731
Mouse anti-VPS35	Santa Cruz Biotechnology	374372
Mouse anti-PCNA	Cell Signalling Technology	2586s
Rabbit anti-CCDC53	Proteintech	24445-1-AP
Rabbit anti-SWIP	Bethyl Laboratories	A304-919A-T
Rabbit anti-ARPC5	Abcam	AB51243
Mouse anti-FLAG	Merck	F1804
Rabbit anti-GLUT1	Abcam	AB115730
Mouse anti-GM130	BD Biosciences	610823
Goat anti-Mouse IgG (H + L) Cross-Adsorbed Secondary Antibody, Alexa Fluor™ 488	Invitrogen	A-11001
Goat anti-Mouse IgG (H + L) Cross-Adsorbed Secondary Antibody, Alexa Fluor™ 568	Invitrogen	A-11004
Goat anti-Rabbit IgG (H + L) Cross-Adsorbed Secondary Antibody, Alexa Fluor™ 488	Invitrogen	A-11008
Goat anti-Rabbit IgG (H + L) Cross-Adsorbed Secondary Antibody, Alexa Fluor™ 568	Invitrogen	A-11011
Goat anti-Mouse IgG (H + L) Cross-Adsorbed Secondary Antibody, Alexa Fluor™ 647	Invitrogen	A-21235
Goat anti-Rabbit IgG (H + L) Cross-Adsorbed Secondary Antibody, Alexa Fluor™ 647	Invitrogen	A-21244
Goat anti-Rat IgG (H + L) Cross-Adsorbed Secondary Antibody, Alexa Fluor™ 568	Invitrogen	A-11077
Anti-mouse IgG, HRP-linked Antibody	Cell Signalling Technology	7076s
Anti-rabbit IgG, HRP-linked Antibody	Cell Signalling Technology	7074s
Anti-rat IgG, HRP-linked Antibody	Cell Signalling Technology	7077s
**Oligonucleotides and other sequence-based reagents**		
gRNA TRIM62	Eurofins Scientific	GCAGCTGCTCAAGCGACAAC
siRNA sequences	Qiagen	See Appendix Table S2
RT-PCR Primers	Eurofins Scientific	See Appendix Table S3
**Chemicals, Enzymes and other reagents**		
Alexa Fluor™ 488-EGF	Invitrogen	E13345
Alexa Fluor™ 488-Tfn	Invitrogen	T13342
Alexa Fluor™ 488-CTxB	Invitrogen	C34775
FITC-labeled Dex	Invitrogen	D1822
Hoechst 33342	Thermo Scientific	62249
Phalloidin Alexa Fluor™ 568	Invitrogen	A12380
SP-DiOC_18_(3) (3,3’-Dioctadecyl-5,5’-Di(4-Sulfophenyl) Oxacarbocyanine, Sodium Salt)	Invitrogen	D7778
Octadecyl Rhodamine B Chloride (R18)	Invitrogen	O246
DMEM, high glucose, GlutaMAX™ Supplement	Gibco	#10566016
0.25% Trypsin EDTA (1X)	Gibco	25200-056
MEM Non-Essential amino acid (NEAA)	Gibco	#11140050
Penicillin Streptomycin Glutamine	Gibco	#10378016
Fetal bovine serum	Merck	F2442
BSA	HIMEDIA	MB083
DMSO (dimethyl sulfoxide)	Merck	D8418
CK666	Merck	SML0006
Bafilomycin A1	Merck	B1793
Cycloheximide	Merck	C1988
Chlorpromazine	Merck	C8138
EIPA	MedChemExpress	L593754
Brefeldin A	Merck	B6542
Monensin	Invitrogen	00-4505-51
Low melting agarose	Genaxy	GEN-LE-100
TPCK trypsin	Merck	T1426
Crystal violet solution	HIMEDIA	S012
Lipofectamine™ RNAimax	Invitrogen	13778-150
Lipofectamine™ LTX Reagent with PLUS™ Reagent	Invitrogen	#15338100
Importazole	Cell Signalling Technology	10451 s
HEPES	HIMEDIA	MB016
Paraformaldehyde (PFA)	HIMEDIA	TC703
Polyethylenimine, branched (PEI)	Merck	408727
Polybrene	Merck	H9268
Protease inhibitor cocktail	Merck	P8340
TRI Reagent	Merck	T9424
Nuclease-Free Water	Qiagen	129114
Apilimod	Merck	SML2974
Brefeldin A	Merck	B6542
IPTG	Merck	16758
eBioscience™ Monensin Solution (1000X)	Invitrogen	00-4505-51
Lithium acetate dihydrate	Merck	L6883
Poly(ethylene glycol)	Merck	P4338
Adenine	Merck	A8626
Yeast Synthetic Drop-out Medium Supplements –Leu/–Trp	Merck	Y0750
Yeast media, Dropout (DO) Supplement –Ade/–His/–Leu/–Trp	TaKaRa	630428
GFP-Trap® Agarose	Proteintech	gta
Myc-Trap® Agarose	Proteintech	yta
Anti-DYKDDDDK Tag (L5) Affinity Gel	BioLegend	651501
Glutathione Resin	G-Biosciences	786-311
HisPur™ Cobalt Resin	Thermo Fisher Scientific	89965
Clarity™ Western ECL Substrate	Bio-Rad	170-5060
Fluoromount-G®	Southern Biotech	0100-01
iTaq™ Universal SYBR® Green Supermix	Bio-Rad	1725121
**Software**		
GraphPad Prism 9	GraphPad	NA
CRISPOR	https://crispor.gi.ucsc.edu/	NA
ImageJ (2.0.0-rc-69/1.52p)	ImageJ	NA
Zen 3.4 blue	Zeiss	NA
CellProfiler (2.2.0)	CellProfiler	NA
KNIME (3.7.2)	KNIME	NA
Adobe illustrator	Adobe	NA
**Other**		
Duolink^®^ In Situ Red Starter Kit Mouse/Rabbit	Merck	DUO92101-1KT
Cell Line Nucleofector^®^ 2b Kit T	Lonza	VCA-1002
Protein Labeling Kit RED-NHS 2nd Generation	NanoTemper Technologies	MO-L011
PrimeScript™ 1st strand cDNA Synthesis Kit	TaKaRa	6110A
Q5^®^ High-Fidelity DNA Polymerase	New England Biolabs	M0491S

### Cell lines

A549, MDCK, HeLa, HepG2, and HEK293T cells were purchased from American Type Culture Collection (ATCC) and Huh-7 cells were purchased from Merck, and cultured in Dulbecco’s modified Eagle medium (DMEM) (Gibco, 10566016), supplemented with 10% fetal bovine serum (FBS) (Merck, F2442), 1x Minimum Essential Medium (MEM) non-essential amino acids (Gibco, 11140050), 1x penicillin-streptomycin-glutamine (Gibco, 10378016) and were maintained at 37 °C in 5% CO_2_. All cell lines were routinely tested and confirmed to be free of mycoplasma contamination. Cells were maintained at 70–80% confluency, whereas TRIM62 knockout (KO) cells were maintained at ~60% confluency.

### Viruses, bacteria, and yeast

Purified IAV X-31 (an H3N2 reassorted strain derived from the A/Puerto Rico/8/34 (PR8) and A/Hong Kong/1/68 strains was purchased from Microbiologics (previously Virapur), USA and stored at −80 °C until use. The median tissue culture infectious dose (TCID_50_) of purified viruses was determined as 1.95 × 10^9^ infectious units/ml. The IAV strains NYMC_X311 (A/Brisbane/1/2018) (H3N2), Udorn (A/Udorn/72) (H3N2), WSN (A/WSN/1933) (H1N1), Semliki Forest virus (SFV-ZsG-GFP) and Vaccinia-GFP (VV-GFP) were kind gifts from Yohei Yamauchi (ETH Zurich, Switzerland). Vesicular stomatitis virus (VSV-GFP) was a kind gift from Santosh Chauhan (CCMB Hyderabad, India). All IAV strains were propagated in MDCK cells and stored at −80 °C until use. SFV-ZsG-GFP and VV-GFP were propagated in BHK-21, and VSV-GFP was propagated in Vero E6. Virus stocks were stored at −80 °C until use. All experiments involving viruses were conducted in a Biosafety Level 2 (BSL-2) laboratory at IISER Mohali in accordance with the biosafety guidelines. The study was reviewed and approved by the Institutional Biosafety Committee (IBSC), IISER Mohali (approval number: BT/IBKP/471/2021). Bacterial transformations were done in chemically competent Stbl3 strain (Invitrogen, C737303) of *Escherichia coli* (*E. coli*). Recombinant protein expression for protein purification was carried out in *E. coli* Rosetta (DE3) cells (Merck, 70954). Yeast 2-hybrid experiments were performed in the Y2H Gold strain of *Saccharomyces cerevisiae* (Takara Bio, Inc.) (kind gift from Mahak Sharma, IISER Mohali).

### Plasmids and transfection

All plasmids used in this study are listed in Appendix Table S1. Plasmids for transient overexpression were generated by inserting amplified DNA into pEGFP-N1 or pEGFP-C1. Plasmids for establishing stable cell lines were generated by inserting amplified DNA into pLenti CMV GFP puro (Addgene #17448). For plasmid transfection into A549 cells, Lipofectamine™ LTX Reagent with PLUS™ Reagent (Invitrogen, 15338100) was used as per the manufacturer’s protocol. For transfection of HEK293T cells, 1 µg of plasmid DNA was transfected using 3 µl of 0.1% polyethylenimine (PEI; Merck, 408727) at a DNA:PEI ratio of 1:3 (w/v). Prior to plasmid transfection, cell culture medium was replaced with fresh culture medium. DNA/PEI mixture was incubated at room temperature (RT) for 15 min and then added to the cells.

### Generation of TRIM62 knockout (KO) cell line

To generate TRIM62 KO in A549 cells, CRISPR/Cas9 technique was employed. Guide RNA (gRNA) sequence (GCAGCTGCTCAAGCGACAAC) targeting TRIM62 genomic region was designed using CRISPOR (https://crispor.gi.ucsc.edu/), synthesized and cloned into pSpCas9(BB)-2A-Puro (PX459) (Addgene #62988) vector. The gRNA encoding plasmid was transfected into A549 cells using the Cell Line Nucleofector® 2b Kit T (Lonza, VCA-1002) according to the manufacturer’s protocol. In summary, 1 × 10^6^ cells were harvested by centrifugation at 90 × *g* for 10 min, and the cell pellet was resuspended in 100 µl of Solution T. Immediately, 4 µg plasmid DNA was added to the resuspended cells and the sample was loaded into the cuvette and pulsed twice using program X-001 in Nucleofector^TM^ 2b (Lonza). Transfected cells were incubated at 37 °C for 3 days in CO_2_ incubator and then passaged and validated for KO by sequencing and quantitative reverse transcription polymerase chain reaction (qRT-PCR).

### Generation of cell lines stably expressing TRIM62 and its mutants

TRIM62-His wild-type (WT) and mutants (RING^m^, ΔRING, ΔB-box, ΔCC, ΔP-S) were stably expressed in TRIM62 KO cells using the lentiviral vector system. To generate lentiviruses encoding TRIM62 or its mutants, HEK293T cells were transfected with pCMVR8.74 (Addgene #22036) and pMD2.G (Addgene #12259) lentiviral packaging plasmids along with TRIM62-His (wild type, WT) or mutants cloned in the pLenti CMV GFP puro vector, using PEI. Six to eight hours after transfection, cells were washed with 1x phosphate-buffered saline (PBS) and prewarmed cell culture medium was added. Cell culture medium containing viruses were collected 72 h post-transfection and centrifuged to remove cellular debris. TRIM62 KO cells were infected with lentiviruses containing polybrene (1 µg/ml) (Merck, H9268). Sixty to seventy-two hours post transduction, cells were passaged and used for experiments after confirming the expression of His using indirect immunofluorescence (IIF).

### siRNA transfection

siRNAs targeting human genes were purchased from Qiagen. Non-targeting/scrambled siRNA (siControl) and siATP6V1B2 were used as negative and positive controls, respectively. For siRNA-mediated knockdown (KD), A549 cells were reverse transfected with 10 nM siRNA using Lipofectamine™ RNAiMAX (Invitrogen, 13778-150) and incubated with the siRNA for 72 h. All siRNAs used in this study are listed in Appendix Table S2. KD efficiency of each siRNA was validated by qRT-PCR or western blotting or both. Primers used for qRT-PCR in this study are listed in Appendix Table S3. qRT-PCR was performed using iTaq™ Universal SYBR® Green Supermix (Bio-Rad, 1725121) on a CFX96 system (Bio-Rad). Data analysis was performed using 2^−ΔΔCT^ method.

### RNAi screening of TRIM proteins for IAV infection and vRNP nuclear import

Three independent siRNAs targeting 20 TRIM proteins were purchased from Qiagen. A549 cells were reverse-transfected with 10 nM siRNA using Lipofectamine™ RNAiMAX, and incubated at 37 °C for 72 h in CO_2_ incubator. Seventy-two hours post-transfection, cells were infected with purified IAV (X-31, H3N2) at a multiplicity of infection (MOI) of 0.01 for 10 h. Purified viruses were suspended in infection medium (50 mM HEPES, 0.2% BSA in DMEM). Bafilomycin A1 (BafA1) (Merck, B1793) was used as an inhibitor of virus infection. After 10 h post infection (h.p.i.), cells were washed with PBS and fixed with 4% formaldehyde at RT for 20 min, following which IIF was performed to detect viral nucleoprotein (NP). Cells were imaged using a high-content confocal quantitative image cytometer (CQ1, Yokogawa). NP intensity was quantified using ImageJ software and average intensity per cell was calculated. Hits were selected based on the criterion that 2 out of 3 siRNAs should show >50% decrease in NP intensity. Using this criterion 15 genes were selected which were further evaluated for viral ribonucleoprotein (vRNP) nuclear import.

For the screen evaluating vRNP nuclear import, A549 cells were transfected with siRNAs and after 72 h post-transfection, cells were infected with 0.25 µl of 1.95 × 10^9^ infectious units/ml of IAV (X-31) per well of 96-well plate for 4 h. Cycloheximide (CHX; Merck, C1988) was added to the medium at 1 mM concentration. Two hours post addition, the infection medium was replaced with fresh medium containing 1 mM CHX to ensure potent and sustained inhibition of the translational machinery. Importazole (Ipz; 40 nM; Cell Signalling Technology, 10451s) was used as a positive control and was maintained throughout the assay. Four hours post virus addition, cells were washed with PBS and fixed with 4% formaldehyde at RT for 20 min and then IIF was performed to stain viral NP. Cells were imaged using CQ1.

All experiments were carried out in three experimental triplicates and nine (3 × 3) z-stack images were acquired from a single well of a 96-well optical bottom plate (Greiner, 655090). Imaging was performed using a 20x or 40x objective lens in CQ1. Images were stacked and maximum intensity projections were taken for image analysis using CellProfiler, KNIME, and ImageJ software.

### IAV infection in different cell lines

Hela, HepG2, and Huh-7 cells were reverse-transfected with 10 nM siRNA using Lipofectamine RNAiMAX, and incubated at 37 °C in CO_2_ incubator. siATP6V1B2 was used as a positive control. Forty-eight hours post-transfection, HeLa cells and 72 h post-transfection HepG2 and Huh-7 cells were infected with purified IAV (X-31) at a multiplicity of infection (MOI) of 0.01 for 10 h. Purified viruses were suspended in infection medium (50 mM HEPES, 0.2% BSA in DMEM). After 10 h post infection (h.p.i.), cells were washed with PBS and fixed with 4% formaldehyde at RT for 20 min, following which IIF was performed to detect viral nucleoprotein (NP).

### Indirect immunofluorescence (IIF)

Fixed cells were permeabilized using permeabilization solution (PS) (5% FBS, 1% BSA, and 0.1% saponin in 1x PBS) at RT for 30 min. Cells were incubated with primary antibody diluted in PS at RT for 2 h and then washed three times with PBS for 10 min each. After washing, cells were further incubated with Alexa Fluor conjugated secondary antibody at RT for 1 h. Nucleus (DNA) was stained with Hoechst (Thermo Scientific, 62249). After washing the cells with PBS (10 min for each washing), imaging was performed using CQ1. Image analysis was performed using CellProfiler, KNIME, and ImageJ.

Primary antibodies used were mouse anti IAV-NP (HB65, 1:50), mouse anti IAV-M1 (HB64, 1:30) from ATCC; mouse anti IAV-HA (H3SKE, 1:100), mouse anti IAV-HA (A1, 1:1500), rabbit anti IAV-HA (Pinda, 1:5000). Rabbit anti-Lamp1 (9091s, 1:500), rabbit anti-His tag (12698s, 1:400), rabbit anti-EEA1 (3288s, 1:300) from Cell Signalling Technology; mouse anti-EEA1 (610457, 1:300), mouse anti-GM130 (610823, 1:300) from BD Biosciences; rabbit anti-FAM21 (PA5-35883, 1:500), rabbit anti-WASH1 (PA5-51731, 1:100) from Invitrogen; rabbit anti-CCDC53 (24445-1-AP, 1:500) from Proteintech; rabbit anti-ARPC5 (AB51243, 1:100) and rabbit anti-GLUT1 (AB115730, 1:500) from Abcam; mouse anti-VPS35 (374372, 1:250) from Santa Cruz Biotechnology; and rat anti-Myc tag (9E1, 1:400) from ChromoTek. Secondary antibodies used were goat anti-mouse IgG (H + L) Cross-Adsorbed Secondary Antibody, Alexa Fluor™ 488 (A-11001, 1:1000), Alexa Fluor™ 568 (A-11004, 1:500) and Alexa Fluor™ 647 (A-21235, 1:500), goat anti-rabbit IgG (H + L) Cross-Adsorbed Secondary Antibody, Alexa Fluor™ 488 (A-11008, 1:1000), Alexa Fluor™ 568 (A-11011, 1:500) and Alexa Fluor™ 647 (A-21244, 1:500), and goat anti-rat IgG (H + L) Cross-Adsorbed Secondary Antibody, Alexa Fluor™ 568 (A-11077, 1:500). F-actin was stained with Phalloidin Alexa Fluor™ 568 (Invitrogen, A12380).

### IAV entry assays

IAV entry assays were performed as previously described (Pathak et al, 2025; Banerjee et al, 2013). Briefly, for IAV binding assay, A549 cells were incubated with 0.25 µl of 1.95 × 10^9^ infectious units/ml of IAV (X-31) per well of 96-well plate at 4 °C for 45 min. After incubation, cells were washed with cold infection medium (50 mM HEPES, 0.2% BSA in DMEM) twice and fixed with 4% formaldehyde, and IIF was performed with anti-HA antibody (Pinda), Hoechst, and Phalloidin Alexa Fluor™ 568.

For IAV endocytosis assay, cells were incubated with 0.25 µl of 1.95 × 10^9^ infectious units/ml of IAV (X-31) per well of 96-well plate at 37 °C for 30 min. Chlorpromazine (Cpz; 25 µg/ml; Merck, C8138) was used as a positive control and cells were pretreated for 10 min prior to infection. For CK-666 and methyl-β-cyclodextrin (MβCD) experiments, cells were pretreated with the respective inhibitors for 1 h prior to infection. In all cases, inhibitors were maintained throughout the virus incubation period. After 30 min of virus internalization, cells were washed with PBS and fixed with 4% formaldehyde. Following fixation, cells were washed with PBS and blocking solution (BS, 5% FBS and 1% BSA in 1x PBS) was added for 1 h. Cells were incubated with rabbit HA (Pinda, 1:1000) in BS at 4 °C overnight to block non-internalized HA epitopes. After incubation, cells were washed three times with PBS for 10 min each, followed by incubation with a secondary anti-rabbit AF647 conjugate. Cells were washed three times with PBS and fixed again with 4% formaldehyde to stabilize the secondary antibody bound to the Pinda antibody. Cells were then permeabilized with PS for 30 min and incubated with mouse HA1 (1:100) at RT for 2 h to detect internalized HA epitopes. After incubation, cells were washed with PBS and incubated with secondary anti-mouse AF488 conjugate and Hoechst and Phalloidin Alexa Fluor™ 568 at RT for 1 h. Cells were washed again with PBS and imaging was performed at 40x in CQ1.

For HA acidification and M1 uncoating assays, cells were incubated with 0.25 µl of 1.95 × 10^9^ infectious units/ml of IAV (X-31) per well of 96-well plate at 37 °C for 60 min and 120 min, respectively. BafA1 (50 nM) was used as a positive control and maintained during virus incubation. After incubation for the respective durations, cells were washed with PBS and fixed with 4% formaldehyde. For vRNP nuclear import assay, cells were incubated with 0.25 µl virus of 1.95 × 10^9^ infectious units/ml of IAV (X-31) per well of 96-well plate for 4 h. Cells were washed with PBS and fixed with 4% formaldehyde. IIF was performed to detect the acid conformation of HA using an acid conformation-specific antibody (A1) for HA acidification assay, mouse anti-M1 (HB-64) for M1 uncoating assay, and mouse anti-NP (HB-65) for vRNP nuclear import assay.

For IAV fusion assay, purified IAV (X-31) particles were labeled with lipophilic dyes SP-DiOC18 (Invitrogen, D7778) and R18 (Invitrogen, O246). The dyes were dissolved in absolute ethanol at a working concentration of 33 µM and 67 µM, respectively. After the preparation of the dye mixture, 10 µl of IAV (X-31) (1.95 × 10^9^ infectious units/ml) was diluted in 140 µl of PBS, following which 1.8 µl of the dye mixture was added to the suspended viruses with constant low speed mixing. The virus was incubated with the dye at 4 °C for 60 min with continuous rotation at low speed. After the virus was labelled, it was diluted in infection medium and passed through a hydrophobic filter before use. A549 cells were then incubated with 1 µl labelled virus per well at 4 °C for 45 min. After virus binding, cells were washed with cold infection medium and shifted to 37 °C for 90 min. Cells were then washed with PBS and fixed with 4% formaldehyde.

### IAV plasma membrane (PM)-bypass uncoating and infection assay

IAV (X-31) PM-bypass uncoating was performed as previously described (Banerjee et al, 2014). Briefly, siRNA transfected cells were incubated with 1 µl of 1.95 × 10^9^ infectious units/ml of IAV (X-31) per well of 96-well plate at 4 °C for 45 min. After binding, the virus-containing medium was discarded and the cells were washed with cold infection medium. Stop medium (50 mM HEPES pH 7.4, 20 mM NH_4_Cl in DMEM) was added in control wells. To induce virus fusion at the PM, cells with bound viruses were incubated with the fusion medium (50 mM citrate buffer in DMEM, pH 5.0) at 37 °C for 2 min in CO_2_ incubator. Immediately after 2 min, the fusion medium was removed, stop medium was added and cells were incubated at 37 °C for another 5 min. Cells were fixed with 4% formaldehyde. IIF was performed against IAV M1 (HB64, 1:30) and imaging was performed at 40x in CQ1.

For PM-bypass infection, cells were processed in a similar manner for viral fusion at the PM as described above. Following viral fusion, the cells were incubated at 37 °C for 10 h to allow viral gene expression. Cells were fixed with 4% formaldehyde, and IIF was performed against IAV NP (HB65, 1:50) and imaging was performed at 20x in CQ1.

### Infection assay for Semliki Forest virus (SFV), Vaccinia virus (VV), and Vesicular stomatitis virus (VSV)

For SFV, VV, and VSV, we employed recombinant viruses tagged with GFP, that included SFV-ZsG-GFP (SFV), VV-GFP (VV), and VSV-GFP (VSV). Cells were infected with SFV-ZsG-GFP and VV-GFP for 24 h and VSV-GFP for 10 h. All the viruses were suspended in infection medium (50 mM HEPES and 0.2% BSA in DMEM). BafA1 was used as an inhibitor for SFV, VV and VSV infection, while 5-(N-ethyl-N-isopropyl)-amiloride (EIPA) (MedChemExpress, L593754), specific macropinocytosis inhibitor was used for VV infection. siCLTC and siRAC1 were used as positive controls for SFV and VV infection, respectively. For SFV and VV, cells were fixed after 24 h of infection with 4% formaldehyde at RT for 20 min. Cells were fixed after 10 h for VSV infection. Nuclei were stained with Hoechst. IIF was performed to detect GFP signal. Cells were imaged using CQ1.

### EGF, transferrin, and cholera toxin B uptake assays

For epidermal growth factor (EGF) uptake, transfected cells were serum-starved for 20 h in DMEM. Cpz was used as a positive control. Cells were pretreated with Cpz at 37 °C for 10 min. Alexa Fluor™ 488-EGF (30 ng/ml in serum-containing medium) (Invitrogen, E13345) was then added to the cells, and cells were incubated at 37 °C for 30 min in a CO₂ incubator. Cpz was maintained throughout the incubation period in positive-control wells. After incubation, plates were shifted to 4 °C, washed with ice-cold PBS, treated with ice-cold acid wash buffer (150 mM NaCl, 50 mM Glycine, pH-3.0) for 2 min to remove surface-bound EGF, washed again with PBS, and fixed with 4% formaldehyde at RT. Cells were counterstained with Hoechst and imaging was performed using CQ1.

For transferrin (Tfn) uptake, transfected cells were serum-starved for 4 h in DMEM. siCLTC was used as a positive control. Alexa Fluor™ 488-Tfn (30 µg/ml in serum-free medium) (Invitrogen, T13342) was added to the cells and allowed to bind at 4 °C for 45 min. Cells were then washed three times with ice-cold PBS, shifted to 37 °C in serum-free medium for 10 min to allow internalization, and immediately shifted to 4 °C to arrest its uptake. After washing with ice-cold PBS, cells were treated with acid wash buffer for 2 min to remove surface-bound Tfn, washed again, and fixed with 4% formaldehyde at RT, followed by Hoechst staining and imaging.

For cholera toxin B (CTxB) uptake, transfected cells were serum-starved for 2 h in DMEM. MβCD (10 mM) was used as a positive control and cells were pre-treated with MβCD at 37 °C for 1 h. Alexa Fluor™ 488-CTxB (750 ng/ml in serum-free medium) (Invitrogen, C34775) was then added to the cells and allowed to bind at 4 °C for 45 min. After binding, cells were washed three times with ice-cold PBS, then incubated in serum-free medium at 37 °C for 10 min or 30 min for internalization. MβCD was maintained during binding and uptake in positive-control wells. Plates were then shifted to 4 °C, washed with ice-cold PBS, treated with acid-wash buffer for 2 min to remove surface-bound CTxB, washed again with PBS, and fixed in 4% formaldehyde at RT. IIF was carried out to stain the nuclei with Hoechst and early endosomes with anti-EEA1 antibody (10 min uptake) or the Golgi complex with anti-GM130 antibody (30 min uptake).

### Dextran uptake assay

For dextran (Dex) uptake, control wells were pre-treated with 50 µM EIPA for 30 min at 37 °C. Plate was incubated at 4 °C for 10 min to arrest endocytic pathways. FITC-labelled Dex (Invitrogen, D1822) was diluted to 100 µg/ml in serum-free DMEM, sonicated and added to the cells, followed by incubation at 37 °C for 30 min in a CO₂ incubator. In positive-control wells, EIPA was maintained during uptake. After incubation, the plate was shifted to 4 °C to arrest its uptake, and cells were washed thrice with PBS. Cells were then fixed with 4% formaldehyde at RT, stained with Hoechst, and imaging was performed.

### Western blotting

For protein detection by western blotting, cells were lysed using cell lysis buffer (150 mM NaCl, 50 mM Tris pH 7.4, 1% Nonidet P-40, 0.1% SDS and 0.5% deoxycholate, protease inhibitor cocktail). The lysate was incubated at 4 °C for 30 min with vortexing every 10 min followed by centrifugation at 17,000 × *g* at 4 °C for 20 min. Supernatants were collected and mixed with 6x SDS loading dye. Samples were boiled at 95 °C for 10 min and resolved by 12.5% SDS-PAGE at 90 V for 2 h. Separated proteins were transferred to nitrocellulose membrane at 90 V for 1.5 h followed by membrane blocking with 5% skimmed milk or 5% BSA in Tris-buffered saline containing 0.1% Tween-20 (TBST) for 1.5 h. Membrane was incubated with primary antibody overnight at 4 °C. The membrane was washed with 0.1% TBST for 5 min, three times at RT, and further incubated with an HRP-tagged secondary antibody at RT for 1 h followed by three washes for 10 min each. Signal was detected using Clarity™ Western ECL Substrate (Bio-Rad, 170-5060).

Primary antibodies used were mouse anti IAV-NP (HB65, 1:50), mouse anti IAV-M1 (HB64, 1:30) from ATCC; rabbit anti-GAPDH (2118 s, 1:2000), mouse anti-PCNA (2586 s, 1:1000) from Cell Signalling Technology; rabbit anti-FAM21 (PA5-35883, 1:1000), rabbit anti-WASH1 (PA5-51731, 1:300) from Invitrogen; mouse anti-VPS35 (374372, 1:1000), mouse anti-His tag (sc-53073, 1:1000) from Santa Cruz Biotechnology; rat anti-Myc tag (9E1, 1:1000) from ChromoTek; rabbit anti-GFP purified antibody from Abgenex (1:2500); rabbit anti-CCDC53 (24445-1-AP, 1:500) from Proteintech; rabbit anti-SWIP (A304-919A-T, 1:2000) from Bethyl Laboratories; mouse anti-FLAG (F1804, 1:1000) from Merck. Secondary antibodies used were anti-mouse IgG, HRP-linked antibody (7076s, 1:2000), anti-rabbit IgG, HRP-linked antibody (7074s, 1:2000), and anti-rat IgG, HRP-linked antibody (7077s, 1:2000) from Cell Signalling Technology.

### Co-immunoprecipitation assay

HEK293T cells were transfected with the plasmids expressing the proteins of interest. After 12–14 h, cells were lysed with ice-cold Lysis buffer (10 mM Tris/Cl pH 7.5, 150 mM NaCl, 0.5 mM EDTA, 0.5% NP-40). The lysate was incubated at 4 °C for 30 min, with extensive pipetting at every 10 min. The lysate was then centrifuged at 17,000 × *g* at 4 °C for 10 min. The supernatant was collected and 5% of it was taken as input fraction. The remaining supernatant was incubated with Myc-Trap® Agarose (Proteintech, yta) or GFP-Trap® Agarose (Proteintech, gta) or anti-DYKDDDDK Tag (L5) Affinity Gel (FLAG-Trap, BioLegend, 651501) at 4 °C for 2 h with rotation at 13 rpm. After incubation, antigen-conjugated agarose beads were washed three times with ice-cold wash buffer (10 mM Tris/Cl pH 7.5, 150 mM NaCl, 0.05% NP40, 0.5 mM EDTA). The protein complexes were then eluted from the beads by boiling them at 95 °C for 10 min. Beads were again centrifuged at 500 × *g* for 1 min and the supernatant was loaded onto 12.5% SDS-PAGE gel. Western blotting was performed with specified antibodies.

### Native-immunoprecipitation assay

Native-immunoprecipitation was performed to detect endogenous WASH1 with VPS35-GFP as per the protocol described previously (Zavodszky et al, 2014). Briefly, HEK293T cells were transfected with plasmids expressing the proteins of interest. Twelve to fourteen hours post-transfection, cells were lysed with ice-cold Lysis buffer (20 mM HEPES-KOH, pH 7.2, 50 mM K-acetate, 200 mM sorbitol, 2 mM EDTA, 0.1% Triton X-100). The lysate was incubated at 4 °C for 20 min, with mild pipetting. The lysate was then centrifuged at 16,000 × *g* at 4 °C for 10 min. The supernatant was collected and 5% of it was taken as input fraction. The remaining supernatant was incubated with GFP-Trap® Agarose (Proteintech, gta) at 4 °C for 4 h with rotation at 9 rpm. After incubation, antigen-conjugated agarose beads were washed five times with ice-cold wash buffer (20 mM HEPES-KOH, pH 7.2, 50 mM K-acetate, 200 mM sorbitol, 2 mM EDTA, 0.1% Triton X-100). The protein complexes were then eluted from the beads by boiling them at 95 °C for 10 min. Beads were again centrifuged at 500 × *g* for 1 min and the supernatant was loaded onto 12.5% SDS-PAGE gel. Western blotting was performed with specified antibodies.

### Confocal and Airyscan high-resolution imaging

For confocal imaging, cells were seeded on coverslips (VWR®, 631-0148) and experiments were performed. Cells were then fixed with 4% formaldehyde for 20 min at RT and IIF was performed as previously described and then the coverslips were mounted on glass slides using Fluoromount-G® (Southern Biotech, 0100-01) and left overnight to dry at RT. Imaging was performed with a 63x oil immersion lens in Leica SP8 upright confocal microscope. Images were acquired with 0.3 µm interval along the z-axis and were further stacked during image processing using ImageJ.

For co-localization of WASH1 and CTxB at the cell surface and on endosomes, Alexa Fluor™ 488-CTxB (750 ng/ml in serum-free medium) was added to the A549 cells and allowed to bind at 4 °C for 45 min. After binding, cells were washed three times with ice-cold PBS and fixed with 4% formaldehyde at RT for 20 min. To visualize internalized CTxB, cells were washed with ice-cold PBS after binding and shifted to 37 °C for 10 min to allow internalization before fixation. IIF was performed using anti-WASH1 antibody.

To visualize the localization of CTxB with IAV, Alexa Fluor™ 488-CTxB and IAV were added simultaneously to the A549 cells and allowed to bind at 4 °C for 45 min to examine surface-bound cargoes. For intracellular localization, cells were shifted to 37 °C for 10 min to allow internalization of both CTxB and IAV before fixation. IIF was performed to detect IAV-HA.

For GLUT1 localization, siTRIM62-transfected cells were treated with brefeldin A (1 µM; Merck, B6542) or monensin (1×; Invitrogen, 00-4505-51) at 37 °C for 1 h. Cells were then fixed with 4% formaldehyde at RT for 20 min and stained with anti-GLUT1 and anti-GM130 antibodies.

Airyscan high-resolution imaging was performed with 63x oil immersion lens in ZEISS LSM 980 Elyra 7 super-resolution microscope equipped with a monochrome cooled high-resolution AxioCamMRm Rev. 3 FireWire(D) camera with Airyscan 2.0 processing. To detect co-localization of TRIM62-Myc with WASH1 or FAM21, A549 cells were seeded on coverslips and transfected with plasmid encoding TRIM62-Myc. Cells were then fixed with 4% formaldehyde. IIF was performed to detect TRIM62-Myc and endogenous WASH1 or FAM21 and imaging was performed.

### Live-cell imaging

For live-cell imaging, A549 cells were grown in 35 mm cell culture dishes (ibidi, 81156) and transfected with GFP-WASH1 (mouse) and RFP-Rab7 using Lipofectamine™ LTX Reagent with PLUS™ Reagent. After 24 h, time-lapse videography was performed with 63x oil immersion lens in Zeiss LSM 980 Airyscan 2.0 equipped with CO_2_ gas chamber maintained at 37 °C. Before the initiation of image acquisition, cells were treated with 1 µM apilimod and incubated at 37 °C for 40 min in CO_2_ incubator, then videos were recorded.

For detection of viruses in WASH1-positive compartments, A549 cells were transfected with GFP-WASH1 (mouse) using Lipofectamine™ LTX Reagent with PLUS™ Reagent. After the GFP expression, cells were incubated with R18-labelled IAV (X-31) at 4 °C for 45 min. After binding, the virus-containing medium was discarded and the cells were washed with cold infection medium and viruses were allowed to internalize at 37 °C for 15 min in CO_2_ incubator and then live cell imaging was performed.

### Super-resolution structured illumination microscopy (SIM)

Super-resolution structured illumination microscopy (SIM) was performed on fully motorized lattice SIM system Model ZEISS SIM 5 (Carl Zeiss Ltd.) system equipped with Plan-Apochromat 63×/1.4 oil immersion objective lens, High resolution and Back Thin sCMOS camera (Hamamatsu Fusion BT) and laser-based filter sets with precisely mounted ACR-coded filter modules. Samples were mounted on a XY Piezo Scanning Stage. Image acquisition was performed with Z Stacks with 13 phase images for a single fluorophore on a single plane to acquire the raw data. Final processed image was generated using the ZEN Blue software with the SIM² algorithm to generate super-resolution images with ~50–70 nm resolution.

### IAV plaque assay

A549 WT and TRIM62 KO cells were seeded in a 6-well plate. At 60% confluency, cells were infected with IAV (WSN, H1N1) at an MOI of 0.01 and incubated at 37 °C for 24 h in CO_2_ incubator. Twenty-four h.p.i., the cell supernatant containing egressed viruses was collected and centrifuged at 300 × *g* to remove cellular debris. Supernatant was collected and diluted to 10^1^, 10^2^, 10^4^, and 10^6^ times in infection medium and then added to the monolayer of MDCK cells seeded in a 12-well plate and cells were incubated at 37 °C for 2 h in CO_2_ incubator. Two h.p.i., cells were washed with infection medium and then overlaid with warm infection medium containing 0.6% low melting agarose (Genaxy, GEN-LE-100), 1x penicillin-streptomycin-glutamine and 1 µg/ml TPCK-Trypsin (Merck, T1426). Agarose was allowed to solidify at RT for 15 min and then plates were shifted to 37 °C for 48 h in CO_2_ incubator. Plaques were observed and cells were fixed with 8% formaldehyde at RT for 60 min. After fixation, the agarose plugs were carefully removed without disturbing the cells and were stained with crystal violet blue for 15 min with constant shaking at low speed. Plates were then washed with water to remove extra stain. Plaques were counted and plaque-forming units (PFU)/ml was calculated.

### Proximity ligation assay (PLA)

Cells were transfected with siControl and siTRIM62 in a 96-well plate and fixed with 4% formaldehyde 72 h post-transfection. PLA was performed on fixed cells using Duolink® In Situ Red Starter Kit (Merck, DUO92101) according to the manufacturer’s protocol. All incubations were done at 37 °C in humidified chamber. In summary, blocking solution was added to the wells and the plate was incubated for 1 h. After blocking, primary antibodies were diluted in antibody-diluent, added in the wells, and incubated for 2 h. After incubation, cells were washed twice with wash buffer A for 10 min each and anti-rabbit plus and anti-mouse minus probes were added for 1 h. Cells were washed with wash buffer A and incubated with ligase for 30 min, followed by washing. In the last step, polymerase was added and the plate was incubated for 100 min. Cells were washed with wash buffer B for 10 min twice and the last washing was done with 0.01x wash buffer B for 1 min. Hoechst was diluted in PBS and cells were stained and imaged in CQ1.

For analysis of TRIM62-Myc and TRIM62ΔP-S-Myc interactions, A549 cells stably expressing TRIM62-Myc and TRIM62∆P-S-Myc were seeded in 96-well plate and fixed with 4% formaldehyde and PLA was performed.

### Yeast 2-hybrid (Y2H) assay

To check direct protein-protein interaction by Y2H, TRIM62 was cloned in the pGAD-T7 vector and WASH subunits were cloned in the pGBK-T7 vector. *Saccharomyces cerevisiae* Y2H Gold cells were made competent using a modified LiOAc method. Competent yeast cells were co-transformed with 200 ng each of the pGAD-T7 and pGBK-T7 plasmids by adding six-fold volume of 40% filtered polyethylene glycol (PEG, Merck, P4338). This suspension was mixed well and incubated at 30 °C for 30 min. Following this incubation, cells were shifted at 42 °C for 25 min. Cells were then plated onto plates containing synthetic medium lacking the amino acid leucine and tryptophan and incubated at 30 °C for 72 h until growth was observed. Single colonies of transformed yeast cells were selected, diluted in 100 µl of filtered autoclaved water. Then, a six-fold serial dilution of transformed colonies was spotted in both control plate (-leucine -tryptophan) and the selection plate (-leucine -tryptophan -histidine). Plates were incubated at 30 °C for 72–96 h till growth was observed. Growth on plate containing -histidine selection medium indicated interaction between two proteins.

### Protein purification

*E. coli* Rosetta (DE3) strain was transformed with recombinant plasmid encoding TRIM62-His (full-length), TRIM62 P-S-His or FAM21(1-356)-GST. The protein expression was induced with Isopropyl β-D-1-thiogalactopyranoside (IPTG; Merck, I6758). Cells were lysed using sonication, and the lysate was fractionated into supernatant and pellet by centrifugation. The TRIM62-His FL/TRIM62 P-S-His or FAM21(1-356)-GST was purified from supernatant using metal affinity chromatography (Co2^+^-NTA) (Thermo Fisher Scientific, 89965) or glutathione beads (G-Biosciences, 786-311), respectively. TRIM62-His FL/TRIM62 P-S-His were eluted from Co2^+^-NTA using 300 mM imidazole. FAM21 (1–356 aa)-GST was eluted using 20 mM glutathione. The protein purity was confirmed using SDS-PAGE.

### Microscale thermophoresis (MST)

The interaction of FAM21(1-356) with TRIM62 (full-length) and TRIM62 P-S was examined using MST. FAM21(1-356) was labelled using monolith NT Protein labeling kit RED-NHS (NanoTemper Technologies, MO-L011) as per the manufacturer’s instructions. For binding studies, 100 nM of FAM21(1-356) was incubated with TRIM62 FL or TRIM62 P-S serially diluted from 100 μM to 3.05 nM. The reaction mixtures were then loaded into Monolith™ NT.115 standard capillaries (NanoTemper, MO-K022) and MST measurements were performed using Monolith NT.115 instrument (NanoTemper Technologies). The dose–response curve obtained by plotting MST signal (ΔFnorm) at varying concentration of TRIM62 (full-length) or TRIM62 P-S was analyzed using MO Affinity Analysis.

### Image processing and analysis

All images at 20x and 40x objectives were acquired using CQ1. Experiments were carried out in 96-well optical-bottom Greiner plates. The focal plane was set automatically using nuclei (stained with Hoechst) as a reference. Z-stacks were acquired within a 20 µm range of the fixed center. Nine fields were selected from one well, and the same fields were imaged across all sample wells. Maximum intensity projection (MIP) images were generated in TIFF format for each laser channel. For quantifying infection percentage, image analysis pipelines were built in CellProfiler (v2.2.0) and KNIME (v3.7.2) as previously described (Miyake et al, 2019).

For NP intensity, ImageJ (2.0.0-rc-69/1.52p) was used. Thresholding for nuclei was done to count cell numbers, and NP signals were thresholded for particle analysis. Thresholded area × mean intensity was normalized to the cell number to calculate NP intensity per cell. Data were plotted in GraphPad Prism 9, and statistical analysis was done using one-way ANOVA with multiple comparisons. Intensity data analysis for IAV entry assays were similarly performed.

For IAV binding assays, surface-bound HA protein, F-actin, and nuclei were stained. F-actin marked the cellular area, was Gaussian-blurred and thresholding was done. F-actin mask was applied to the HA channel to extract HA signal. HA intensity for endocytosis and acidification assays was calculated in the same way. For M1 uncoating, cells with dispersed M1 were counted as positive, and percentage uncoating was calculated relative to total nuclei. For dispersed M1 intensity, a 40 × 40 square ROI was drawn in the cytoplasm, and mean intensity was measured. For vRNP nuclear import, cells positive for nuclear NP were scored, and percentage was calculated. To quantify nuclear NP intensity, a nuclear mask was created from nuclei and applied to the NP channel.

For co-localization analysis, images were acquired with a 63x oil immersion on the Leica SP8 upright confocal microscope at 1024 × 1024 resolution with z-stacks. Mander’s correlation coefficients (MCC) were calculated using the JaCoP plugin in ImageJ.

Co-localization of TRIM62-Myc with WASH1 or FAM21 was examined using ZEISS LSM 980 Elyra 7 in super-resolution mode with Airyscan processing. Co-localization of WASH complex subunits with VPS35 was analyzed using lattice SIM system Model ZEISS SIM 5 with SIM^2^ processing. Images were converted to 8-bit before analysis. Intensity-distance profiles were generated by drawing a line across co-localized regions and plotting signal intensity changes using the profile tool in Zen 3.4 Blue software.

For image presentation, Hoechst staining intensity was adjusted independently to optimize nuclear visualization. For all other channels, identical brightness and contrast settings were applied to all samples within the same experiment.

### Statistical analysis

All data are presented as mean ± standard deviation (SD) from at least three independent experiments (*N* ≥ 3). Statistical analyses were performed using GraphPad Prism 9. Differences between groups were assessed using either one-way ANOVA with Dunnett’s multiple-comparisons test or an unpaired two-tailed *t*-test, as appropriate. For box and whiskers plots, the center line indicates the median, the box boundaries represent the 25th and 75th percentiles (interquartile range), and the whiskers extend to the minimum and maximum values. Violin plots depict the distribution of the data. *P* values < 0.05 were considered statistically significant.

## Supplementary information

Appendix

Peer Review File

Source data Fig. 1

Source data Fig. 2

Source data Fig. 3

Source data Fig. 4

Source data Fig. 5

Source data Fig. 6

Expanded View Figures

## Data Availability

This study includes no data deposited in external repositories. The source data of this paper are collected in the following database record: biostudies:S-SCDT-10_1038-S44319-026-00942-x.
