# Supplementary material for: TRIM62 facilitates influenza A virus entry by regulating WASH-dependent endosomal trafficking
Source: EMBO Rep. 2026 Sep 19;27(20):5953–90. doi: 10.1038/s44319-026-00942-x (PMC13694701; doi:10.1038/s44319-026-00942-x)
Supplement: Supplementary file 1 [file 44319_2026_942_MOESM1_ESM.pdf]

Appendix to

'TRIM62 facilitates influenza A virus entry by regulating WASH-dependent endosomal trafficking'

Table of contents

Appendix Figure S1 ---2
Appendix Figure S2 ---3
Appendix Figure S3 ---5
Appendix Figure S4 ---6
Appendix Table S1 ---7
Appendix Table S2 ---8
Appendix Table S3 ---8

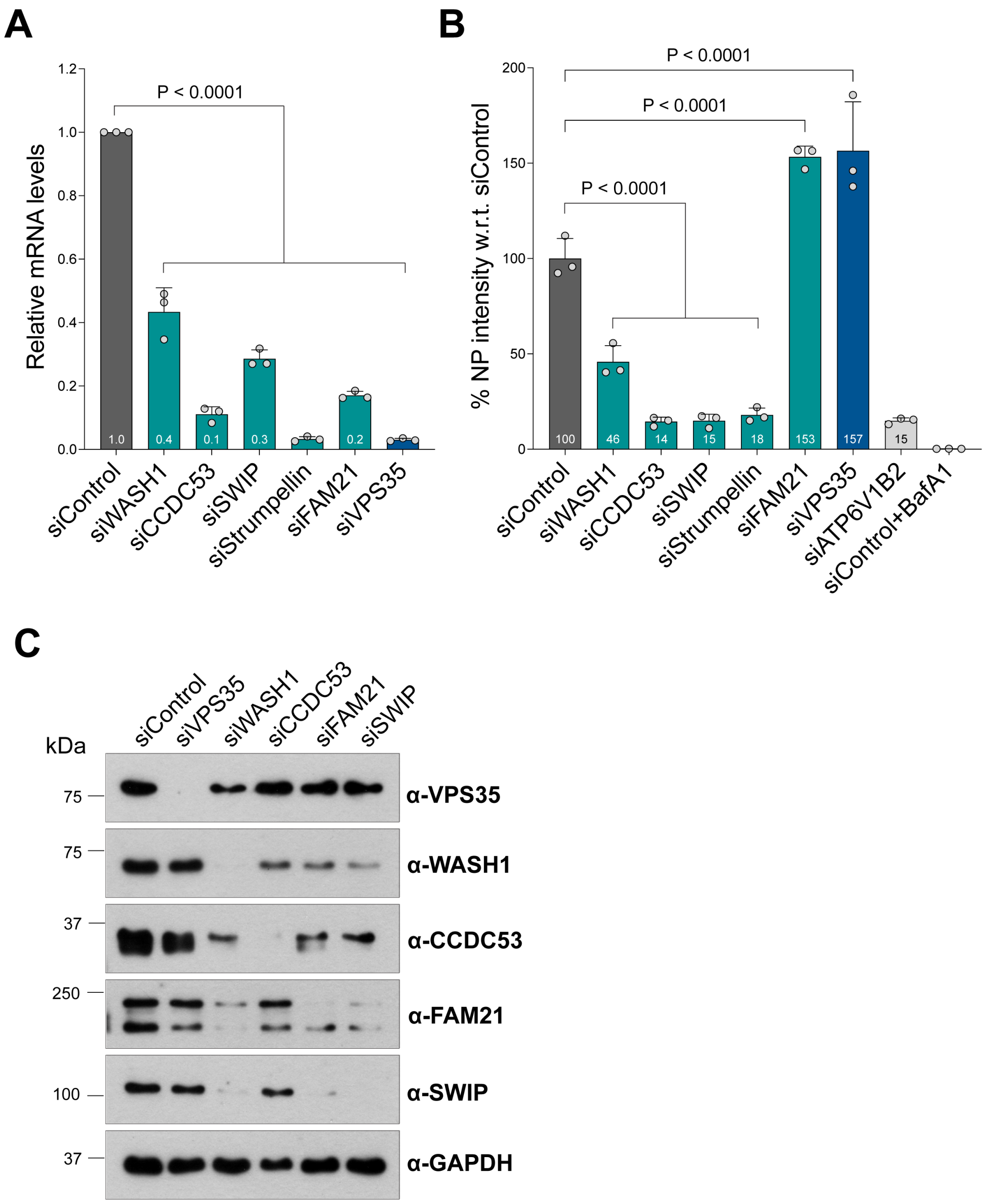

Appendix Figure S1. WASH1 and VPS35 oppositely regulate IAV infection, while VPS35 depletion does not affect the protein levels of WASH subunits.

(**A**) Validation of siRNA-mediated knockdown (KD) efficiency of WASH subunits and VPS35 by qRT-PCR (N = 3 independent experiments; mean ± SD; error bars represent SD; one-way ANOVA). P values for each comparison: $P < 0.0001$. **(B)** Quantification of IAV infection (% NP expression at 10 h.p.i., normalized) in A549 cells infected with IAV (X-31 strain; MOI = 0.01) in an RNAi screen targeting WASH subunits and VPS35. $n \geq 2000$ cells/siRNA treatment (N = 3 independent experiments; mean ± SD; error bars represent SD; one-way ANOVA). P values for each comparison: $P < 0.0001$. (**C**) Western blots showing the effects of KD of individual WASH subunits and VPS35 at the protein levels of others. The western blots are representative of three independent experiments.

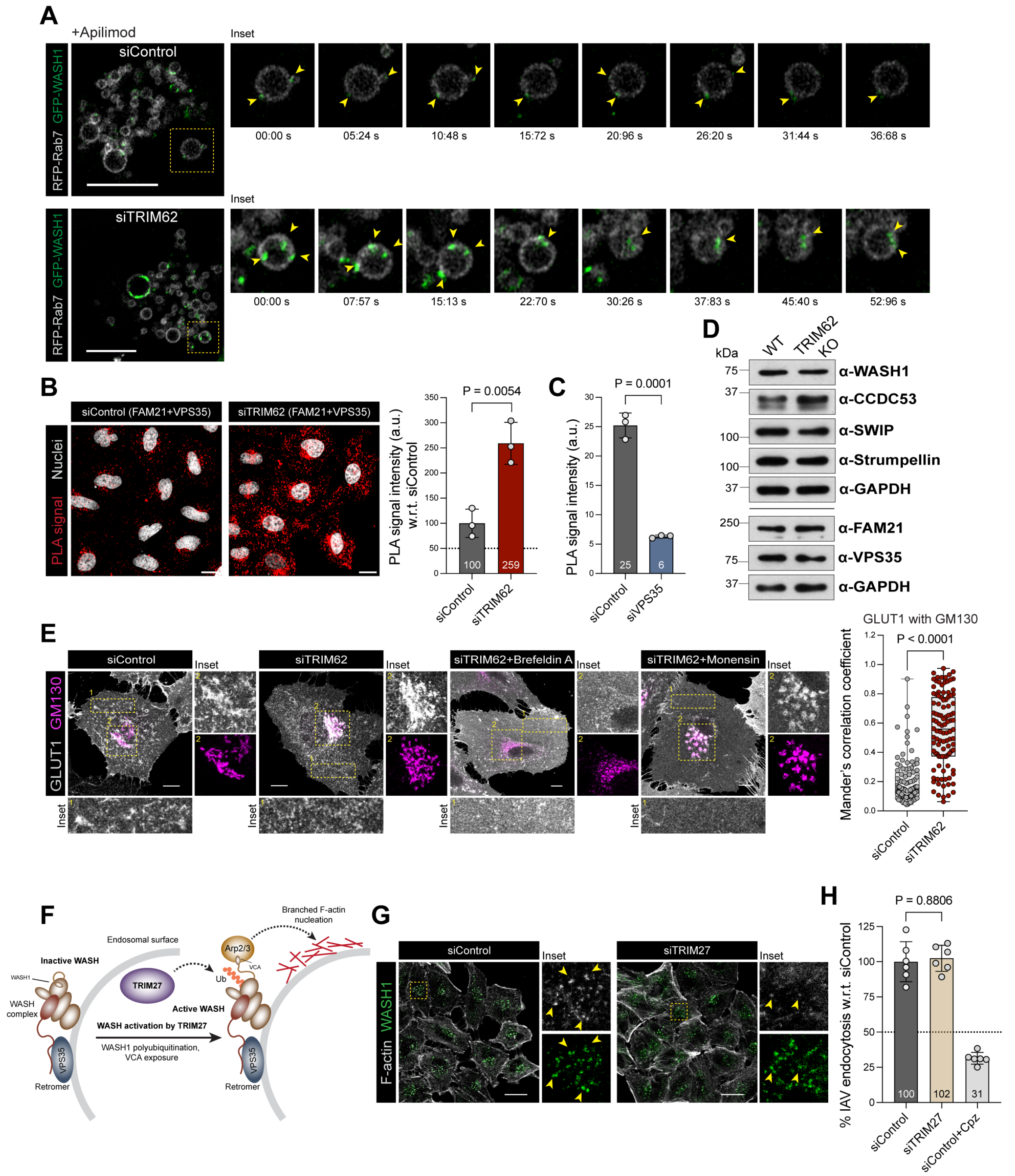

Appendix Figure S2. Reduced WASH1 NPF activity upon TRIM62 depletion leads to defects in cargo sorting, not IAV endocytosis.

(**A**) High-resolution, time-lapse images of a representative endosome (outlined by the box) in a cell expressing RFP-Rab7 (grey) and GFP-WASH1 (mouse; green) in control and TRIM62 KD A549 cells treated with apilimod (1 µM). Foci of GFP-WASH1 (mouse) on the endosome are indicated by yellow arrowheads. Scale bars, 10 µm. (**B**) High-content confocal images (left) and quantification (right) of PLA signals from FAM21-VPS35 interaction in HeLa cells. Scale bars, 20 µm. $n \geq 500$ cells/sample ($N = 3$ independent experiments; mean ± SD; error bars represent SD; unpaired two-tailed t-test; $P = 0.0054$). (**C**) Quantification of PLA signals from FAM21-VPS35 interaction in control and VPS35 KD HeLa cells. $n \geq 500$ cells/sample ($N = 3$

independent experiments; mean ± SD; error bars represent SD; unpaired two-tailed *t*-test; $P = 0.0001$). (**D**) Western blots showing the protein levels of WASH subunits and VPS35 in WT and TRIM62 KO cells. The western blots are representative of three independent experiments. (**E**) Confocal microscopy images (left) showing GLUT1 (grey) and GM130 (magenta) in HeLa cells with or without the treatment of brefeldin A (1 µM) or monensin (1x) for 1 h. Insets show GLUT1's Golgi-localization and surface distribution. Scale bars, 10 µm. Quantification (right) of GLUT1-GM130 colocalization (without brefeldin A or monensin treatment), based on Mander's correlation coefficient. $n = 104$ cells/sample (N = 3 independent experiments; unpaired two-tailed t-test; $P = 8.78 \times 10^{-27}$). Data are represented as box and whisker plots where the centre line indicates the median, the box boundaries represent the 25th and 75th percentiles (interquartile range), the whiskers extend to the minimum and maximum values, and all individual data points are overlaid. (**F**) Schematic showing WASH1 activation by TRIM27. TRIM27 ubiquitinates WASH1 exposing the VCA domain which is crucial for the recruitment and activation of the Arp2/3 complex for branched actin nucleation at endosomes. (**G**) Confocal microscopy images showing F-actin (grey) and WASH1 (green) in control and TRIM27 KD A549 cells. Yellow arrowheads indicate the distribution of F-actin at WASH1-positive endosomes. Scale bars, 20 µm. (**H**) Quantification of IAV endocytosis at 30 min post-infection, in control and TRIM27 KD A549 cells. $n \geq 4000$ cells/sample ($N = 3$ independent experiments; mean ± SD; error bars represent SD; one-way ANOVA). *P* value for siControl vs siTRIM27: $P = 0.8806$.

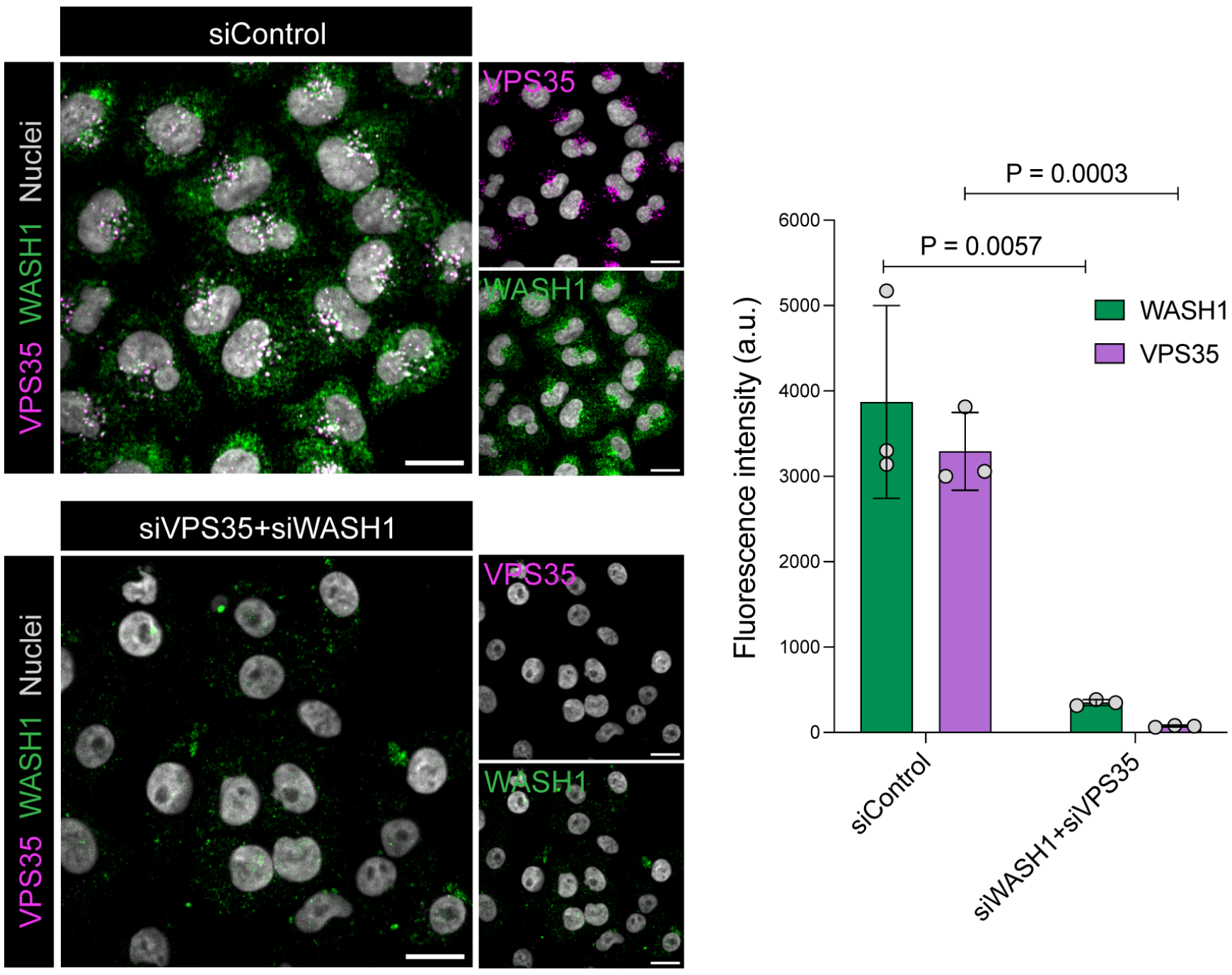

Appendix Figure S3. KD validation of siWASH1+siVPS35-treated cells.
High-content confocal images (left) showing VPS35 (magenta) and WASH1 (green) in control and WASH1+VPS35 double KD A549 cells. Scale bars, 20 µm. Quantification (right) of VPS35 and WASH1 intensity. $n \geqslant 1000$ cells/sample ($N = 3$ independent experiments; mean ± SD; error bars represent SD; unpaired two-tailed t-test). P values for siControl vs siWASH1+siVPS35: $P = 0.0057$ (WASH1); $P = 0.0003$ (VPS35).

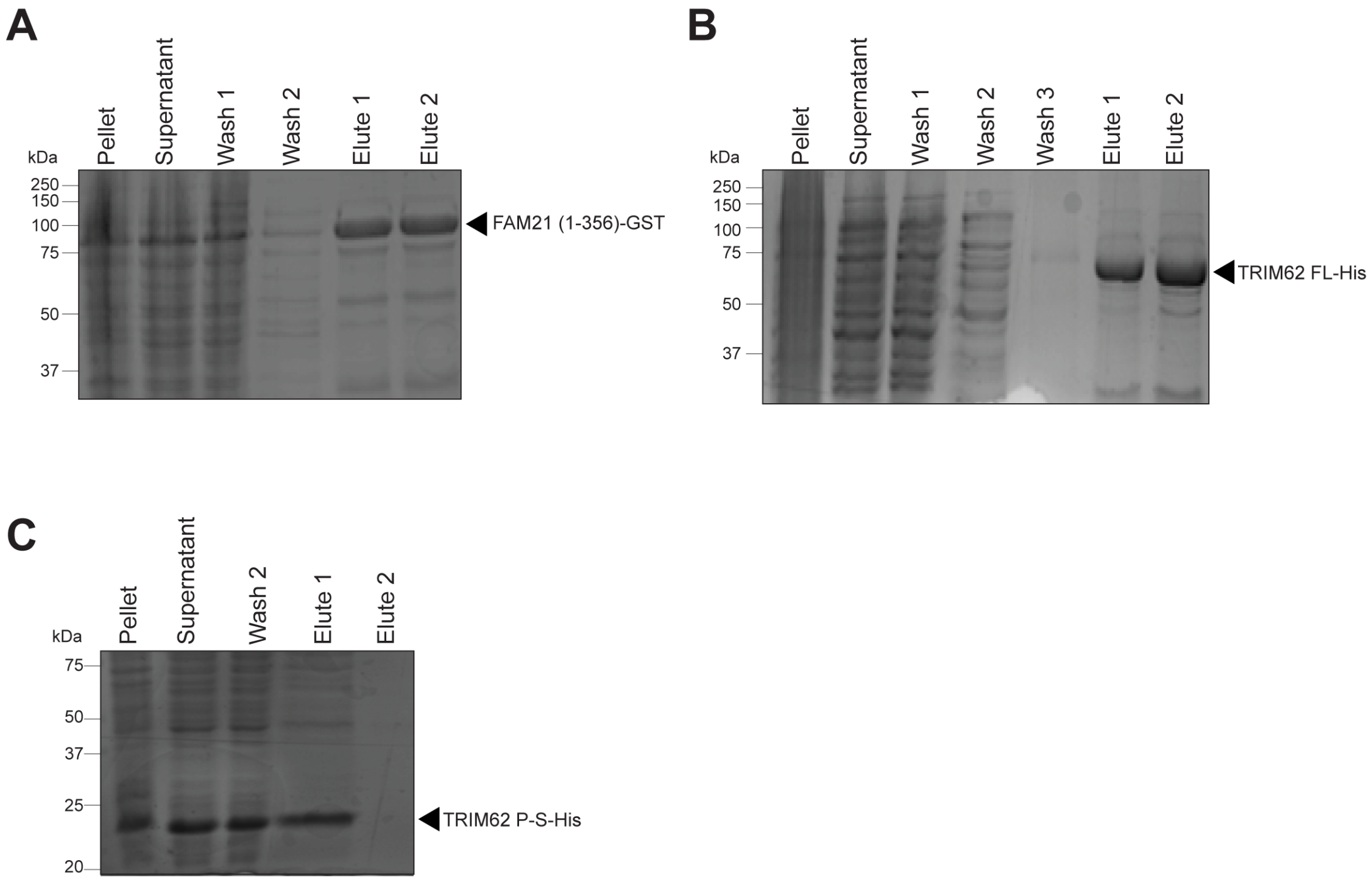

Appendix Figure S4. Purification of recombinant FAM21 globular domain (1-356 aa), TRIM62-FL and TRIM62 P-S using affinity chromatography.

(**A-C**) Coomassie-stained SDS-PAGE gels showing purification profiles of FAM21 globular domain (1-356 aa) (**A**), TRIM62-FL (**B**) and TRIM62 P-S (**C**).

Appendix Table S1. List of plasmids used in this study

Plasmid	Source
TRIM62-His	This study
TRIM62-His mutants	This study
TRIM62-Myc	This study
TRIM62-Myc mutants	This study
MBP-His (pHLmMBP-6)	Addgene #72346
MBP-Myc	This study
GST-Myc	This study
CCDC53-GFP	This study
Strumpellin-GFP	This study
FAM21-GFP	Kind gift from Prof. Lenka Libusova, Charles University
GFP-SWIP	Kind gift from Prof. Lenka Libusova, Charles University
GFP-WASH1	Kind gift from Prof. Alexis Gautreau, CNRS
GFP-mWASH1	Kind gift from Prof. Mahak Sharma, IISER Mohali
VPS35-GFP	Kind gift from Prof. Peter J. Cullen, University of Bristol
VPS26-GFP	This study
GFP-VPS29	Kind gift from Prof. Peter J. Cullen, University of Bristol
TRIM62-pGAD	This study
WASH1-pGBK	This study
CCDC53-pGBK	This study
FAM21-pGBK	This study
SWIP-pGBK	This study
Strumpellin-pGBK	This study
3xFLAG	Kind gift from Dr. Santosh Chauhan, CCMB
FAM21-FLAG	This study
FAM21-FLAG mutants	This study
gTRIM62-PX459	This study
FAM21 (1-356)-GST	This study
TRIM62-His	This study
TRIM62 P-S-His	This study
GFP-Rab5Q67L	Kind gift from Prof. Mahak Sharma, IISER Mohali
CMVR8.74	Banerjee lab
MD2.G	Banerjee lab
RFP-Rab7	Banerjee lab

Appendix Table S2. List of siRNAs used in this study

**Gene**	**siRNA sequence**
siTRIM62	CTGGTTTAATTAGACAAGGAT
siWASH1	CAGCTCCTTGCTGCTCTTCAA
siCCDC53	AAGATATGCCAGATATCTCAA
siSWIP	TCGGGCTTACGTAACTCACTA
siStrumpellin	CAGAGAGTGCCTATCAACGAA
siFAM21	ATGAGGAGGACCAGTGGAATA
siVPS35	CAGAATTGCCCTTAAGACTTT
siACTR3	CCGGCTGAAATTAAGTGAGGA
siWAVE	TGGTTTGGTAATGATCTGCAA
siN-WASP	CAGATACGACAGGGTATCCAA
siTRIM27	GAACCAGCTCGACCATTTATT
siRAC1	ATGCATTTCCTGGAGAATATA
siCLTC	TAATCCAATTCGAAGACCAAT
siATP6V1B2	CACGGTTAATGAAGTCTGCTA

Appendix Table S3. List of qRT-PCR primers used in this study

Gene		Primer Sequence
TRIM62	Forward	TACTGGGAGGTGGTGGTGGC
	Reverse	GTCATCAGCATTGTAGAAGATG
WASH1	Forward	AGAAGCAGCAGCAGAAGGAG
	Reverse	TACGATTCCCAGTCGTCCTC
CCDC53	Forward	GAAGCCACTTCAGAGCAACC
	Reverse	AGCATCTGGCCTCTCAAGAA
SWIP	Forward	TGCATGCAAGGCATTTAGAG
	Reverse	TTGAAAGGGTTTCGGTCATC
Strumpellin	Forward	CGAGGTTGCCAAGCTTACTC
	Reverse	GTCGCTCCCAACTCTTTCAG
FAM21	Forward	GGATCGGGAAGATACAAGCA
	Reverse	TCATCTTGACACGGCTCTTG
VPS35	Forward	CGTGAAGATGGACCTGGAAT
	Reverse	TCCACACGATCAGGGTAACA
