# Supplementary material for: TRIM62 facilitates influenza A virus entry by regulating WASH-dependent endosomal trafficking
Source: EMBO Rep. 2026 Sep 19;27(20):5953–90. doi: 10.1038/s44319-026-00942-x (PMC13694701; doi:10.1038/s44319-026-00942-x)
Supplement: Supplementary file 2 [file 44319_2026_942_MOESM2_ESM.pdf]

EMBO reports

TRIM62 facilitates influenza A virus entry by regulating WASH-dependent endosomal trafficking

Kajal Gupta, Sampurna Pal, Arpit Tyagi, Roohani Bajaj, Tejal Pathak, Gaganpreet Kaur, Deepak Sharma, and Indranil Banerjee

Corresponding author(s): Indranil Banerjee (indranil@iisermohali.ac.in)

Review Timeline:

Transfer Date:	9th Mar 26
Editorial Decision:	11th Mar 26
Revision Received:	11th Jun 26
Editorial Decision:	29th Jul 26
Revision Received:	2nd Aug 26
Accepted:	25th Aug 26

Editor: Martina Rembold

Transaction Report: This manuscript was transferred to EMBO Reports following peer review at The EMBO Journal.

(Note: With the exception of the correction of typographical or spelling errors that could be a source of ambiguity, letters and reports are not edited. Depending on transfer agreements, referee reports obtained elsewhere may or may not be included in this compilation. Referee reports are anonymous unless the Referee chooses to sign their reports.)

Referee #1:

The manuscript from Banerjee and colleagues investigates the role of WASH, TRIM62 and the antagonism of FAM21-VPS35 in regulation of influenza A virus entry. They find that that components of the WASH complex including WASH, CCDC53, SWIP, and Strumpellin regulate viral entry and infection, which is counteracted by FAM21 interacting with the retromer subunit VPS35 - thereby restricting the WASH complex to functioning at endosomal membranes. However, the authors show that a ubiquitin ligase, TRIM62, associates with FAM21, limiting its interaction with VPS35, thus releasing WASH from its endosomal tether to enable IAV endocytic entry and infection. Interestingly, the authors note that this function of WASH is independent of its Arp2/3-mediated F-actin nucleating activity. Altogether, this is an interesting study that further supports the role of WASH in activities apart from its well-described endosomal function. Moreover, it identifies a unique role for TRIM62 in the regulation of WASH complex activity. Despite this, there are several concerns that need to be addressed.

Major concerns:
1. The authors claim that WASH and several other components of the complex (CCDC53, SWIP, Strumpellin) are involved in AIV entry. Is this independent of each other? Can the authors demonstrate that complexes containing any of these components exist when other components are depleted? For example, can WASH form a complex with any of the other components when FAM21 is depleted?
2. The authors show that loss of TRIM62 impairs WASH actin-nucleating function on endosomes like that previously observed for TRIM27. Can the authors provide an explanation for this finding? Is this effect ubiquitination-dependent?
3. Although PLA can be used as a proxy for protein-protein interaction, the authors need to show biochemically that TRIM62 is affecting the interaction of FAM21 with VPS35.
4. Some IF results lack proper quantitation. For example - F-actin MFI for Figure S7.
5. PLA controls are not present for the PLA experiments. These need to be included.
6. In FigS5, which siRNAs are being used in the immunoblotting experiment? Does siWASH1 #1 even work, as it has no impact on NP intensity shown in Figure S5A? Similarly, which siRNAs are being evaluated in Figure S4F?

Minor concerns:
1. The Introduction section is overly long, and in some parts, for example, where TRIM62 is introduced (line 101 and beyond), the authors go on to describe the data in the present study. Much of this should be relegated to the Results or Discussion section of the manuscript. I would recommend cutting the Introduction in half.

2. In many figures and/or figure legends, it is not clear which cell line(s) are being evaluated. This information should be provided when describing the results and clearly indicated in the figure or figure legend.
3. The table provided in the beginning of the Materials and Methods, while important, should be relegated to supplemental material.

Referee #2:

In this study by Gupta et al., the authors investigate how TRIM62 promotes influenza A virus (IAV) infection and identify a novel role for the FAM21-VPS35 complex in regulating IAV endocytosis. Based on a prior genome-wide screen for IAV host factors, the authors compiled a list of 20 TRIM genes and evaluated their role in viral replication via siRNA knockdown. In this screen, KD of TRIM62 reduced IAV infection, along with TRIM9 and TRIM35. The authors subsequently focused on defining the role of TRIM62 in IAV infection and replication. Loss of TRIM62 resulted in reduced endocytosis and uncoating of IAV particles. By interrogating known endocytic pathways, the authors identify the WASH complex as being important for this process. Through immunofluorescence and immunoprecipitation studies, they report that TRIM62 interacts with FAM21 and prevents sequestration of WASH1 within the retromer complex via VPS35. Consistent with this model, KD of FAM21 or VPS35 increased viral endocytosis and uncoating. Overall, the findings are interesting and potentially significant; however, several aspects of the study require clarification and additional data to strengthen the conclusions
Major Concerns:
1. The data are poorly organized, making the manuscript difficult to interpret and follow. Several exploratory datasets (fig 3 &5) could be moved to the supplementary section to streamline the main figures and improve clarity.
2. There is no direct demonstration of co-localization of WASH1, FAM21, and VPS35 simultaneously in control and TRIM62 KO cells. Given the partial co-localization shown for WASH1/FAM21 or WASH1/VPS35, it is important to demonstrate co-localization of all three proteins together. In addition, there is no evidence showing IAV co-localization with WASH1- or TRIM62-containing compartments. The statement in LN425-425 that TRIM62 interacts with WASH1 only via FAM21 is not sufficiently supported. Can TRIM62 directly interact with WASH1? Does KD of FAM21 disrupt the TRIM62-WASH association?
3. The conclusions would be strengthened by performing concurrent deletion of TRIM62 with FAM21 or VPS35 and assessing restoration of the phenotype. Such epistasis experiments would help define pathway hierarchy.

4. All experiments were performed in A549 cells. Validation in primary lung epithelial cells would substantially strengthen the physiological relevance of the findings.
5. TRIM62 protein expression in WT, KO, and complemented cells should be shown clearly by western blot and immunofluorescence to confirm knockout and rescue efficiency.
6. In Fig 1G and 1I, NP expression appears reduced in IAV-infected TRIM62 KO cells, suggesting possible post-entry defects. Please include multicycle replication data using multiple IAV strains. In addition, include a control virus that is not affected by TRIM62 loss. Preferably, test a virus that enters exclusively via CME or macropinocytosis to distinguish entry-specific effects.
7. In Fig 3 and LN 334-338, if KD of individual WAVE/WASH components destabilizes the entire complex, including WASH1 (Fig S5B), it is unclear why CTB uptake is not affected similarly to siWASH1 KD. This discrepancy requires clarification.
8. In Fig 5A and 5C, endosomes appear enlarged or altered in siTRIM62 cells compared to controls, suggesting broader defects in endosomal compartments. Could these structural changes contribute to altered IAV uptake? It is important to demonstrate that there are no global defects in viral replication in KO or KD cells by testing an unrelated virus.
9. Previous studies suggest that CPZ does not significantly affect IAV infection. Therefore, the strong inhibition of IAV endocytosis by CPZ in this study is surprising. The CPZ concentrations appear higher than in prior reports. Please include cell viability data for all siRNA KD and inhibitor treatments to rule out nonspecific toxicity.

Minor Concerns:
1. What are the predicted off-target effects of the TRIM62 sgRNA?
2. Include average NP expression values in Fig 1A and 1C.
3. For LN 208-210, visualization of M1 uncoating in TRIM62 KO complemented cells would improve clarity.
4. LN 225-228: The DENV data in Fig S4A are not statistically significant; the text should reflect this.
5. Fig 3 should be streamlined to highlight the most relevant data, with remaining CTB experiments moved to the supplementary section.
6. Using consistent color coding for specific molecules within figures would improve readability.
7. The dual siRNA KD experiment is difficult to interpret. Are both targets knocked down in the same cells?
8. In Fig 6G-I, it is unclear how the authors conclude that VPS35 intensity is unchanged in TRIM62 KO cells. Quantification and representative images should better support this statement.
9. In Fig 5N and 5O, the legend states that 70 or 150 cells were analyzed; however, the

panels appear to show fewer samples. Please clarify.

Referee #3:

General comments:

In this manuscript Gupta et al. use siRNA knockdowns to identify TRIM62 as a positive regulator of IAV infection. They use imaging-based virus entry assays to demonstrate a defect in virus uptake, then use model cargo proteins to more broadly investigate the role of TRIM62 in endocytosis. Their subsequent examination of actin regulating complexes leads to the observation that WASH is also important for IAV and CTxB uptake, and they suggest that WASH may be exerting a function at the plasma membrane. They then dissect the function of each WASH subunit in IAV entry, identifying FAM21 as functionally distinct from the others. Finally, they investigate the relationship between WASH, VPS35 and TRIM62, concluding that TRIM62 interacts with FAM21 and negatively regulates the FAM21-VPS35 interaction.

A particular strength of this manuscript is the IAV infection experiments, which are performed to a high standard and presented well. However, data reporting to establish the mechanism of TRIM62's direct regulation of FAM21's association with retromer, and the functional role for FAM21/retromer at cell surface lipid rafts are insufficient to support the core conclusions of the manuscript. A significant amount of additional work will be required to validate these key conclusions.

Specific points:

As WASH and retromer are involved in endosomal protein sorting and recycling, disruption of either of these complexes has a downstream impact on the cell surface levels of hundreds of different proteins. The authors must include a discussion of how their WASH/FAM21/VPS35/TRIM62 knockdowns may affect the protein composition of the cell surface and whether such a global cellular reconfiguration could contribute to the observed endocytosis phenotypes. Retromer perturbation also causes altered RAB7 activity and induces cellular stress, autophagic and lysosomal, the latter likely affecting HA acidification and M1 uncoating independently of WASH. The authors should also discuss these confounding factor in the manuscript and how it impacts on their data interpretation.

The authors conclude that the WASH complex and retromer are enriched at the leading edge in VPS35 suppressed cells. In the imaging data (e.g. Figure 3), only the highlighted cell is showing any 'enrichment' in WASH signal (Fig. 3S). Typically, one would expect to observe enrichment of the cell surface signal at the cell:cell boundaries where two cell surface membranes are juxtaposed. How do the authors exclude an alternative explanation: enrichment of cytosolic WASH1 at 'leading edge protrusions' is a simple consequence of more cytoplasm being present within this highly folded cytosol:membrane region and not a consequence of a specific enrichment at the cell surface.

Extending this, (see line 289-291 and Fig. 3O and 3R), compared to the CTxB signal the VPS35 + FAM21 PLA signal is juxtaposed to the cell surface and does not show a relationship with CTxB signal intensity. High-resolution EM is required to make the conclusion that 'WASH and retromer associate within lipid raft-enriched domains at the plasma membrane'. Otherwise, these signals could simply reflect endosomes positioned close to the plasma membrane, which is a known feature of this network.

The direct association of TRIM62 with FAM21 requires further experimental validation. The Y2H analysis, in a background of some self-activation, is insufficient to conclude the direct nature of this interaction. This is a critical conclusion of the manuscript and hence needs robust validation The authors have provided evidence to map this potential interaction to the PRY-SPRY module of TRIM62 and the N-terminal region of FAM21. Does AlphaFold predict a high confidence score for this potential binding? The authors should certainly isolate these regions as recombinant proteins and perform biochemical assays to establish the direct nature of the binding.

If direct binding is observed, the authors need to consider how TRIM62 binding to the N-terminal region of FAM21 prevents retromer binding to the R19-R20 and R21 LFa repeats at the unstructured C-terminal tail of FAM21.

Minor points:
In all figure legends please indicate which statistical test(s) were used, and in all graphs please indicate where differences are not statistically significant.

Line 65: Include reference to Aitor Hierro's work - Romano-Moreno et al., Protein Sci 33:e4980 (2024).

Line 67-70: May want to include reference to Visweshwaran et al., EMBO J. 37:e97706

(2018) on the assembly of the WASH complex.

Line 123-124. 'When bound to retromer, WASH is spatially constrained and unable to execute productive endosomal trafficking'. The term 'endosomal trafficking' should be removed and replaced with 'endocytic viral entry' to highlight this specific point in the broader endosomal trafficking network.

Lines 173-178: A large number of experiments are discussed very briefly. Please describe the experiments more fully to improve clarity for the reader.

Lines 227-228: The result in Fig. S4A is not statistically significant, please address this fact in the text.

Fig. 2N, Fig. 3B-E, Fig. 4G-H: Please show representative images used for the quantification in the main figure or supplement.

Line 259-261. This is a bold statement given that the data are derived from KD experiments.

Line 266-267. The WASH complex is also recruited to endosomal membrane independently of retromer (Simonetti et al., 2017; Kvainickas et al., 2017) through SWIP association with the endosomal phosphoinositide PI(3,5)P2 (Dostal et al., 2023). An additional, mechanism have been proposed for association with HRS (MacDonald et al., 2018). These points should also be added in the Introduction (line 62).

Line 377: 'renders WASH inactive' is a very strong statement. Where is the evidence to support this.

Fig. 3R: This PLA assay requires appropriate negative controls, such as the EEA1/VPS35 control used in Fig. 6G.

Fig. 5, 6: Please indicate in the figure legend how many times western blots were repeated, and whether results were consistent.

Fig. 6L: In the final lane of this experiment there is low expression of the TRIM62 construct, and IP of the FAM21 357-1341 construct is weak. There is also no indication that this experiment has been repeated. Therefore, the conclusion that FAM21 head deletion disrupts the TRIM62-FAM21 interaction is not robustly supported by the data.

Fig. S5A: Please address the result that siWASH1 #1 has no effect on IAV infection in the text.

Fig. S8G: It is unclear whether the re-expressed TRIM62 constructs all express to comparable levels, which is important for interpreting these data. Please include western blots of the rescue cell lysates to address this.

In siTRIM62 treated cells the early endosomal compartment appears to partially collapse into the perinuclear region. How do the authors disentangle this change in steady state distribution with the increased Mander's correlation coefficient?

Dear Indranil,

Thank you for the transfer of your manuscript to EMBO reports. As discussed, I would like consider your manuscript for publication at EMBO reports if the main reviewer comments can be addressed satisfactorily.

- It will be important to strengthen the evidence that TRIM62 and FAM21 interact directly, to overall provide additional evidence for complex formation, co-localization of all three proteins (WASH1, FAM21, and VP35), and biochemical evidence that TRIM62 affects the interaction of FAM21 with VPS35.
- The data on lipid-raft mediated IAV uptake could be moved to the Supplement, as suggested by referee #2 and the conclusions toned down, unless the specific role of TRIM62 in this uptake-mechanism can be strengthened (along the lines suggested by referee #3).
- The more general effect of WASH and retromer on endosomal protein sorting and recycling and potential effects on RAB7, autophagy and lysosomal function should be discussed.
- Ref#2 suggests testing and unrelated virus to assess whether there is a global effect on viral replication, which might be a good suggestion to test global effects.
- It is not required to elucidate the mechanism of TRIM62 mediated interference with TRIM21-retromer binding (Ref #3, last major point).

Please address all referee concerns in a complete point-by-point response. Acceptance of the manuscript will depend on a positive outcome of a second round of review. It is EMBO reports policy to allow a single round of major revision only and acceptance or rejection of the manuscript will therefore depend on the completeness of your responses included in the next, final version of the manuscript.

If you would like to discuss the revisions further, I am happy to do so at any point either via e-mail or an online call.

We realize that it is difficult to revise to a specific deadline. In the interest of protecting the conceptual advance provided by the work, we recommend a revision within 3 months (11th Jun 2026). Please discuss the revision progress ahead of this time with the editor if you require more time to complete the revisions.

You can either publish the study as a short report or as a full article. For short reports, the revised manuscript should not exceed 27,000 characters (including spaces but excluding materials & methods and references) and 5 main plus 5 expanded view figures. The results and discussion sections must further be combined, which will help to shorten the manuscript text by eliminating some redundancy that is inevitable when discussing the same experiments twice. For a normal article there are no length limitations, but it should have more than 5 main figures and the results and discussion sections must be separate. In both cases, the entire materials and methods must be included in the main manuscript file.

IMPORTANT NOTE: we perform an initial quality control of all revised manuscripts before re-review. Your manuscript will FAIL this control and the handling will be DELAYED if the following APPLIES:
1) A data availability section providing access to data deposited in public databases is missing. If you have not deposited any data, please add a sentence to the data availability section that explains that.
2) Your manuscript contains statistics and error bars based on n=2. Please use scatter blots in these cases. No statistics should be calculated if n=2.

When submitting your revised manuscript, please review the submission guidelines (https://link.springer.com/journal/44319/submission-guidelines#cms-Revised-submissions) and carefully review the instructions that follow below. Failure to include requested items will delay the evaluation of your revision.

1) a .docx formatted version of the manuscript text (including legends for main figures, EV figures and tables). Please make sure that the changes are highlighted to be clearly visible.

2) individual production quality figure files as .eps, .tif, .jpg (one file per figure). See hhttps://media.springernature.com/original/springer-cms/rest/v1/content/27825798/data/v1 for more info on how to prepare your figures.

3) We replaced Supplementary Information with Expanded View (EV) Figures and Tables that are collapsible/expandable online. A maximum of 5 EV Figures can be typeset. EV Figures should be cited as 'Figure EV1, Figure EV2" etc... in the text and their respective legends should be included in the main text after the legends of regular figures.

- For the figures that you do NOT wish to display as Expanded View figures, they should be bundled together with their legends in a single PDF file called *Appendix*, which should start with a short Table of Content. Appendix figures should be referred to in the main text as: "Appendix Figure S1, Appendix Figure S2" etc. See 'Expanded View' section of the submission guidelines.

- Additional Tables/Datasets should be labeled and referred to as Table EV1, Dataset EV1, etc. Legends have to be provided in a separate tab in case of .xls files. Alternatively, the legend can be supplied as a separate text file (README) and zipped together with the Table/Dataset file.

4) a .docx formatted letter INCLUDING the reviewers' reports and your detailed point-by-point responses to their comments. As part of the EMBO Press transparent editorial process, the point-by-point response is part of the Review Process File (RPF), which will be published alongside your paper.

5) a complete author checklist, which you can download from our author guidelines. Please insert information in the checklist that is also reflected in the manuscript. The completed author checklist will also be part of the RPF.

6) Please note that all corresponding authors are required to supply an ORCID ID for their name upon submission of a revised manuscript (<https://orcid.org/>), which can be linked to your profile in the manuscript submission system.

7) Before submitting your revision, primary datasets produced in this study need to be deposited in an appropriate public database. Please remember to provide a reviewer password if the datasets are not yet public. The accession numbers and database should be listed in a formal "Data Availability" section placed after Methods. Please note that the Data Availability Section is restricted to new primary data that are part of this study. * Note - All links should resolve to a page where the data can be accessed. *

If your study has not produced novel datasets, please mention this fact in the Data Availability Section.

8) At EMBO Press we ask authors to provide source data for the main manuscript figures. You will receive a separate email with instructions for providing source data with your revised manuscript, including how to upload and organize the files.

9) Our journal also encourages inclusion of *data citations in the reference list* to directly cite datasets that were re-used and obtained from public databases. Data citations in the article text are distinct from normal bibliographical citations and should directly link to the database records from which the data can be accessed. In the main text, data citations are formatted as follows: "Data ref: Smith et al, 2001" or "Data ref: NCBI Sequence Read Archive PRJNA342805, 2017". In the Reference list, data citations must be labeled with "[DATASET]". A data reference must provide the database name, accession number/identifiers and a resolvable link to the landing page from which the data can be accessed at the end of the reference.

10) Regarding data quantification, the following points must be specified in each figure legend:

- the name of the statistical test used to generate error bars and P values,

- the number (n) of independent experiments (please specify technical or biological replicates) underlying each data point,

- the nature of the bars and error bars (s.d., s.e.m.),

- If the data are obtained from n Program fragment delivered error ``Can't locate object method "less" via package "than" (perhaps you forgot to load "than"?) at //ejpvfs23/sites23b/embor_www/letters/embor_decision_revise_and_review.txt line 54.' 2, use scatter blots showing the individual data points.

Discussion of statistical methodology can be reported in the materials and methods section, but figure legends should contain a basic description of n, P and the test applied.

- Please also include scale bars in all microscopy images.

11) The journal requires a statement specifying whether or not authors have competing interests (defined as all potential or actual interests that could be perceived to influence the presentation or interpretation of an article). In case of competing interests, this must be specified in your disclosure statement.

12) All Materials and Methods need to be described in the main text using our 'Structured Methods' format, which is required for all research articles. According to this format, the Methods section includes a Reagents and Tools Table (listing key reagents, experimental models, software and relevant equipment and including their sources and relevant identifiers) followed by a Methods and Protocols section describing the methods using a step-by-step protocol format. The aim is to facilitate adoption of the methodologies across labs. More information on how to adhere to this format as well as a downloadable template (.docx) for the Reagents and Tools Table can be found in our author guidelines.

We would also welcome the submission of cover suggestions, or motifs to be used by our Graphics Illustrator in designing a cover.

As part of the EMBO publication's Transparent Editorial Process, EMBO reports publishes online a Review Process File (RPF)

to accompany accepted manuscripts. This File will be published in conjunction with your paper and will include the referee reports, your point-by-point response and all pertinent correspondence relating to the manuscript.

You are able to opt out of this by letting the editorial office know (emboreports@embo.org). If you do opt out, the Review Process File link will point to the following statement: "No Review Process File is available with this article, as the authors have chosen not to make the review process public in this case."

I look forward to seeing a revised form of your manuscript when it is ready.

Best regards,

Martina

Martina Rembold, PhD
Senior Editor
EMBO reports

Response to Editor's and Reviewers' comments

Comments from the Editor

- It will be important to strengthen the evidence that TRIM62 and FAM21 interact directly, to overall provide additional evidence for complex formation, co-localization of all three proteins (WASH1, FAM21, and VP35), and biochemical evidence that TRIM62 affects the interaction of FAM21 with VPS35.
- The data on lipid-raft mediated IAV uptake could be moved to the Supplement, as suggested by referee #2 and the conclusions toned down, unless the specific role of TRIM62 in this uptake-mechanism can be strengthened (along the lines suggested by referee #3).
- The more general effect of WASH and retromer on endosomal protein sorting and recycling and potential effects on RAB7, autophagy and lysosomal function should be discussed.
- Ref#2 suggests testing and unrelated virus to assess whether there is a global effect on viral replication, which might be a good suggestion to test global effects.
- It is not required to elucidate the mechanism of TRIM62 mediated interference with TRIM21-retromer binding (Ref #3, last major point).

We thank the editor for providing us the opportunity to revise our work. We carefully addressed all the editorial and reviewers' comments, and addressed them point by point.

- It will be important to strengthen the evidence that TRIM62 and FAM21 interact directly

We thank the editor and reviewers for this important suggestion. In the initial submission, we provided evidence for TRIM62-FAM21 direct interaction using yeast two-hybrid (Y2H) assay together with co-immunoprecipitation (co-IP) analyses. However, as pointed out by the reviewer #3, the Y2H experiments exhibited a degree of self-activation for FAM21 and therefore did not conclusively establish a direct biochemical interaction between the two proteins. To address this concern and strengthen the evidence for a direct interaction between TRIM62 and FAM21, we performed Microscale Thermophoresis (MST) assays using purified recombinant proteins.

In our earlier co-immunoprecipitation (co-IP) experiments with full-length and domain-truncation mutants, we found that the PRY-SPRY domain of TRIM62 interacts with the N-terminal globular domain (1–356 aa) of FAM21. Based on these findings, we sought to test whether this interaction is direct in a purified system. Although we attempted purification of full-length FAM21, we encountered technical difficulties, likely due to the presence of its long intrinsically disordered tail region. We were, however, able to successfully purify the N-terminal globular domain of FAM21. Therefore, MST experiments were performed using the purified N-terminal globular domain of FAM21 together with either full-length TRIM62 or the isolated PRY-SPRY domain of TRIM62.

Our MST analyses demonstrated that both full-length TRIM62 and its PRY-SPRY domain biochemically directly bind the N-terminal globular domain of FAM21, with dissociation constants (K_D) in the nanomolar and micromolar range, respectively (**Fig. 6E,F**). These results provide biochemical evidence for a direct, high to moderate-affinity interaction between TRIM62 and FAM21 and further support our Y2H and co-IP-based mapping data.

- to overall provide additional evidence for complex formation, co-localization of all three proteins (WASH1, FAM21, and VP35)

In the original manuscript, we showed enhanced co-localization of WASH1 and FAM21 with EEA1-positive endosomes upon TRIM62 KD, whereas VPS35 localization remained unperturbed. We calculated co-localization coefficients of WASH1, FAM21, or VPS35 with EEA1 individually and calculated the overall intensity of these markers on EEA1-positive endosomes. (Fig. 5A-D, Fig. 6I,J and Fig. S6A,B in the original manuscript; corresponding to **Fig. 5A-C** and **Fig. EV4A-D** in the revised manuscript).

As suggested by the reviewers, we examined WASH1, FAM21, and VPS35 together using super-resolution structured illumination microscopy (SIM) with SIM^2 processing with an effective resolution of ~50–70 nm. To visualize all the three proteins simultaneously, we over-expressed FAM21-GFP in both control TRIM62 KD cells and indirect-immunofluorescence (IIF) was performed to detect endogenous WASH1 and VPS35. SIM imaging revealed that FAM21-GFP and WASH1 exhibited increased colocalization with VPS35-positive spots in TRIM62 KD cells compared to controls, supporting our previous observations that the association of the WASH complex with VPS35-containing spots is enhanced upon TRIM62 depletion. This data has now been incorporated into **Fig. 5D** of the revised manuscript and discussed in the Results section.

- biochemical evidence that TRIM62 affects the interaction of FAM21 with VPS35.

We thank the editor and reviewers for suggesting the need for biochemical analysis. In the original manuscript, we performed proximity ligation assay (PLA) and observed that TRIM62-depletion enhances both FAM21-VPS35 and WASH1-VPS35 interactions, suggesting increased association of the WASH complex with VPS35-positive endosomal compartments. Consistent with this, expression of the FAM21-interaction-deficient mutant of TRIM62; TRIM62 ΔP-S, also resulted in enhanced FAM21-VPS35 association.

To biochemically examine whether TRIM62 influences the interaction between FAM21 and VPS35, we performed co-IP experiments under native buffer conditions (HEPES-based) by overexpressing VPS35-GFP together with TRIM62-Myc or MBP-Myc (control), followed by pulldown of VPS35 using GFP-Trap beads and probing for endogenous WASH subunits. However, under our experimental conditions, endogenous FAM21 could not be reliably detected in the VPS35 pulldown samples. Given our microscopy data as well as PLA data, which indicated that TRIM62 suppression alters recruitment of the WASH complex to VPS35-positive subdomains, we next assessed the association of another core WASH complex component, WASH1, with VPS35. Notably, VPS35 pull-down samples from TRIM62-Myc–expressing cells contained 46% less endogenous WASH1 than those from MBP-Myc control cells, suggesting that TRIM62 negatively regulates the association of the WASH complex with VPS35 (**Fig. EV4F**), validating our imaging-based and PLA-based findings.

To further validate these results, we examined VPS35-WASH complex association in cells expressing either full-length TRIM62-Myc or its truncated mutant lacking the C-terminal PRY-SPRY domain, which we found to regulate FAM21-mediated recruitment of WASH to VPS35-positive subdomains. We found that, in cells expressing the PRY-SPRY deletion mutant (TRIM62 ΔP-S), ~48% more WASH1 was co-immunoprecipitated with VPS35 compared to cells expressing full-length TRIM62. These findings suggest that loss of the PRY-SPRY domain enhances VPS35-WASH complex association, highlighting its regulatory function (**Fig. EV5C**). Together, these data biochemically confirm that TRIM62 negatively regulates the association of the WASH complex with VPS35-containing compartments.

- The data on lipid-raft mediated IAV uptake could be moved to the Supplement, as suggested by referee #2 and the conclusions toned down, unless the specific role of TRIM62 in this uptake-mechanism can be strengthened (along the lines suggested by referee #3).

We thank the editor and reviewers for this suggestion. In response to this suggestion, we have now moved the data related to lipid raft-mediated IAV uptake (previously in **Fig. 3**) to the **Fig. EV3**. Correspondingly, we have revised and moderated the associated interpretations in the main text. We have also rearranged the figures to improve the overall presentation, logical flow, and coherence of the study. Some of the exploratory datasets from Fig. 3 (Fig. 3H, 3R-S) and supplementary Fig. S4A-C have been removed to improve the focus of the study. We believe that these changes have now streamlined the presentation and have improved the clarity and readability of the manuscript.

- The more general effect of WASH and retromer on endosomal protein sorting and recycling and potential effects on RAB7, autophagy and lysosomal function should be discussed.

We agree with the editor and reviewers that discussing our findings in the broader context of endosomal trafficking pathways would strengthen the manuscript. In the revised manuscript, we have now expanded the Discussion section to include the broader roles of the retromer and WASH complexes in endosomal protein sorting and recycling, as well as their potential connections to RAB7 dynamics, autophagy, and lysosomal function. We further discuss how the effects of TRIM62 on WASH-retromer association may influence these interconnected endosomal trafficking pathways.

In addition, we provide evidence for the proposed role of TRIM62 in WASH/retromer-mediated trafficking pathways, we have included new data demonstrating altered localization of GLUT1, a known WASH/retromer cargo, in TRIM62 KD HeLa cells. Specifically, upon TRIM62 depletion, GLUT1 becomes redistributed from the plasma membrane to the Golgi region (**Appendix Fig. S2E**). These findings further support a role for TRIM62 in cargo sorting and trafficking.

- Ref#2 suggests testing and unrelated virus to assess whether there is a global effect on viral replication, which might be a good suggestion to test global effects.

We agree with the reviewer that assessing unrelated viruses is important to determine whether TRIM62 exerts broader effects on viral infection beyond IAV. Accordingly, we examined the effect of TRIM62 depletion on infection by three additional viruses: Semliki Forest virus (SFV), vaccinia virus (VV) and vesicular stomatitis virus (VSV). We found that TRIM62 depletion significantly reduced SFV and VV infection by approximately 30% and 39%, respectively, whereas VSV infection is enhanced by ~42% that did not reach statistical significance (**Fig. EV2G-I**). These findings suggest that TRIM62 may play a broader proviral role across multiple viruses.

- It is not required to elucidate the mechanism of TRIM62 mediated interference with TRIM21-retromer binding (Ref #3, last major point).

We thank the editor for this clarification. In the revised study, we have demonstrated a direct interaction between TRIM62 and FAM21, specifically between the C-terminal PRY-SPRY domain of TRIM62 and the N-terminal globular domain of FAM21. We further show

that the PRY-SPRY domain is important for regulating WASH complex association with VPS35 and that this regulation contributes to efficient IAV entry.

In light of the editor's guidance, we have not pursued additional mechanistic dissection of how TRIM62 modulates the FAM21-retromer interaction in the current study. Instead, we focused on strengthening the main conclusions of the manuscript by incorporating additional biochemical, imaging, and functional evidence supporting a role for TRIM62 in regulating WASH-retromer association and endosomal trafficking.

Referee #1

The manuscript from Banerjee and colleagues investigates the role of WASH, TRIM62 and the antagonism of FAM21-VPS35 in regulation of influenza A virus entry. They find that that components of the WASH complex including WASH, CCDC53, SWIP, and Strumpellin regulate viral entry and infection, which is counteracted by FAM21 interacting with the retromer subunit VPS35 - thereby restricting the WASH complex to functioning at endosomal membranes. However, the authors show that a ubiquitin ligase, TRIM62, associates with FAM21, limiting its interaction with VPS35, thus releasing WASH from its endosomal tether to enable IAV endocytic entry and infection. Interestingly, the authors note that this function of WASH is independent of its Arp2/3-mediated F-actin nucleating activity. Altogether, this is an interesting study that further supports the role of WASH in activities apart from its well-described endosomal function. Moreover, it identifies a unique role for TRIM62 in the regulation of WASH complex activity. Despite this, there are several concerns that need to be addressed.

We thank the reviewer for thorough evaluation of our manuscript and for the valuable suggestions. It is encouraging that the Reviewer found this work interesting. We addressed all the major and minor concerns point by point.

Major concerns:
1. The authors claim that WASH and several other components of the complex (CCDC53, SWIP, Strumpellin) are involved in AIV entry. Is this independent of each other? Can the authors demonstrate that complexes containing any of these components exist when other components are depleted? For example, can WASH form a complex with any of the other components when FAM21 is depleted?

We thank the reviewer for this thoughtful suggestion. In our study, depletion of multiple WASH subunits independently impaired IAV entry, supporting the functional importance of the WASH complex in this process. We further observed that depletion of TRIM62, VPS35, or CCDC53 did not significantly alter the protein levels of individual WASH complex components in A549 cells. In contrast, depletion of WASH1, FAM21, or SWIP affected the levels of other WASH complex proteins (**Appendix Fig. S1C**), suggesting interdependence in complex stability. Moreover, it is important to note that depletion of both FAM21 and SWIP reduced the total protein levels of other subunits but the effects of their depletion on IAV entry were opposite, indicating that although WASH exists as a complex, individual subunits can exert independent effects.

However, it was important to determine whether residual WASH complex subunits remain associated with each other and with VPS35 upon depletion of individual subunits. To address this, we performed super-resolution SIM in WASH1- or FAM21-depleted A549 cells

and examined FAM21-CCDC53-VPS35 or WASH1-CCDC53-VPS35 colocalization. Interestingly in WASH1 KD cells, we observed that while CCDC53 showed reduced co-localization with FAM21 and VPS35, FAM21 showed persistent co-localization with VPS35 indicating a functional FAM21-VPS35 axis (**Fig. 4I**). In FAM21 KD cells (**Fig. 4J**), both WASH complex subunits WASH1 and CCDC53 co-localized with VPS35, indicating that despite reduced protein levels, remaining WASH complex subunits continue to associate with VPS35-positive spots. Interestingly, we found that, in addition to its endosomal localization, WASH1 exhibited increased surface localization upon FAM21 depletion, consistent with a reduced association of the WASH complex with VPS35. These observations have been discussed in the revised manuscript.

Together, these data suggest that depletion of individual subunits does not completely abolish recruitment of remaining WASH complex components to VPS35-positive compartments.

2. The authors show that loss of TRIM62 impairs WASH actin-nucleating function on endosomes like that previously observed for TRIM27. Can the authors provide an explanation for this finding? Is this effect ubiquitination-dependent?

Our findings indicate that TRIM62 depletion impairs WASH-associated actin nucleation on endosomes, reminiscent of previous observations reported for TRIM27. At present, we cannot exclude the possibility that this regulation involves ubiquitination-dependent mechanisms. However, while we appreciate the importance of this question, detailed mechanistic dissection of how TRIM62 regulates WASH NPF activity is beyond the scope of the current study. The primary focus of this work was to define the role of TRIM62 in regulating WASH-retromer association and the impact of this regulation on IAV entry. Moreover, we did not find any role of E3 ubiquitin-ligase activity of TRIM62 in IAV entry. Whether TRIM62 controls WASH-mediated actin nucleation through ubiquitination-dependent mechanisms will require dedicated future investigation.

3. Although PLA can be used as a proxy for protein-protein interaction, the authors need to show biochemically that TRIM62 is affecting the interaction of FAM21 with VPS35.

We thank the reviewer for this important suggestion, which is also addressed in response to the Editor in the beginning. To biochemically examine whether TRIM62 influences the interaction between FAM21 and VPS35, we performed co-IP experiments under native buffer conditions (HEPES-based) by overexpressing VPS35-GFP together with TRIM62-Myc or MBP-Myc (control), followed by pulldown of VPS35 using GFP-Trap beads and probing for endogenous WASH subunits.

However, under our experimental conditions, endogenous FAM21 could not be reliably detected in the VPS35 pulldown samples. Given our microscopy data as well as proximity ligation assay (PLA) data indicated that TRIM62 suppression alters recruitment of the FAM21 as well as WASH1 to VPS35-positive subdomains, we next assessed the association of another core WASH complex component, WASH1, with VPS35. Notably, 46% less endogenous WASH1 was detected in VPS35 pulldown samples from TRIM62-Myc-expressing cells compared to MBP-Myc controls, suggesting that TRIM62 negatively regulates WASH complex association with VPS35 (**Fig. EV4F**), further strengthening our imaging-based analysis and PLA-based detection.

To further validate these findings, we examined VPS35-WASH complex association in cells expressing either full-length TRIM62-Myc or the truncated mutant lacking the C-terminal PRY-SPRY domain, which we found to regulate FAM21-mediated recruitment of the WASH to VPS35-positive subdomains. We found that, in cells expressing the PRY-SPRY deletion mutant (TRIM62 ΔP-S), ~48% more WASH1 was co-immunoprecipitated with VPS35 compared to cells expressing full-length TRIM62. These findings suggest that loss of the PRY-SPRY domain enhances VPS35-WASH complex association, highlighting its regulatory function (**Fig. EV5C**).

Together, these data biochemically confirm that TRIM62 negatively regulates the association of the WASH complex with VPS35-containing compartments.

4. Some IF results lack proper quantitation. For example - F-actin MFI for Figure S7.

We thank reviewer for careful evaluation of this manuscript and pointing out the IF results that lacked quantifications. We have now included quantified data for the IF images that lacked quantification including Figure S7 (now **Fig. EV4I** in the revised manuscript).

5. PLA controls are not present for the PLA experiments. These need to be included.

We agree with the reviewer that adding controls to the PLA data is important. In **Fig. 6H** (**Fig. 5E** in the revised manuscript), we included VPS35-EEA1 PLA as control. The rationale for using this as control is that although VPS35 is present on EEA1-positive early endosome, yet there is no reported interaction between VPS35 and EEA1. Therefore, to check whether the PLA signal between VPS35-FAM21 reflects nanoscale-level proximity, we used this control. We further performed PLA upon FAM21 and VPS35 upon depletion of VPS35 to check the specificity of our experiments. These controls are now separately included in **Appendix Fig. S2C**.

6. In FigS5, which siRNAs are being used in the immunoblotting experiment? Does siWASH1 #1 even work, as it has no impact on NP intensity shown in Figure S5A? Similarly, which siRNAs are being evaluated in Figure S4F?

We used two siRNAs per gene for IAV infection assay (Previously **Fig. S5A,** now **Appendix Fig. S1B**) and observed similar phenotypes for all genes except WASH1 for which only one siRNA showed the phenotype. When we checked transcript levels, we found that the WASH1 siRNA which had no effect on IAV infection also did not efficiently supressed WASH1 transcript level. However, to avoid confusion, we have now included data from validated siRNAs only in the revised manuscript. The sequence for validated siRNAs used in the study are provided in Appendix Table. S3.

Minor concerns:
1. The Introduction section is overly long, and in some parts, for example, where TRIM62 is introduced (line 101 and beyond), the authors go on to describe the data in the present study. Much of this should be relegated to the Results or Discussion section of the manuscript. I would recommend cutting the Introduction in half.

We thank the reviewer for this suggestion. We have now revised the manuscript by shortening the Introduction and including the TRIM62-related information in the Results and Discussion sections.

2. In many figures and/or figure legends, it is not clear which cell line(s) are being evaluated. This information should be provided when describing the results and clearly indicated in the figure or figure legend.

We agree that cell line-related information should have been provided in the figure legends. Most of our experiments were carried out in A549 cells, as this lung-origin cell line closely recapitulates the natural target cells of the virus. Some of the supporting experiments have been done in other cell lines as well. As suggested by the reviewer, we have now provided this information in Results section as well as figure legends.

3. The table provided in the beginning of the Materials and Methods, while important, should be relegated to supplemental material.

We included the table in the Materials and Methods section in our initial submission. Now, we have moved the table to Appendix section as **Appendix Table. S1** in the revised submission.

Referee #2

In this study by Gupta et al., the authors investigate how TRIM62 promotes influenza A virus (IAV) infection and identify a novel role for the FAM21-VPS35 complex in regulating IAV endocytosis. Based on a prior genome-wide screen for IAV host factors, the authors compiled a list of 20 TRIM genes and evaluated their role in viral replication via siRNA knockdown. In this screen, KD of TRIM62 reduced IAV infection, along with TRIM9 and TRIM35. The authors subsequently focused on defining the role of TRIM62 in IAV infection and replication. Loss of TRIM62 resulted in reduced endocytosis and uncoating of IAV particles. By interrogating known endocytic pathways, the authors identify the WASH complex as being important for this process. Through immunofluorescence and immunoprecipitation studies, they report that TRIM62 interacts with FAM21 and prevents sequestration of WASH1 within the retromer complex via VPS35. Consistent with this model, KD of FAM21 or VPS35 increased viral endocytosis and uncoating. Overall, the findings are interesting and potentially significant; however, several aspects of the study require clarification and additional data to strengthen the conclusions

We thank the Reviewer for the careful evaluation of our manuscript and for recognizing the significance and potential impact of this work. The insightful comments and suggestions have helped improve the overall quality of the manuscript. We have addressed all major and minor concerns point by point and have revised the manuscript accordingly.

Major Concerns:

1. The data are poorly organized, making the manuscript difficult to interpret and follow. Several exploratory datasets (fig 3 &5) could be moved to the supplementary section to streamline the main figures and improve clarity.

We agree with the reviewer that the data should have been presented in a more coherent and comprehensive manner for overall clarity and improved flow of the results. We have now thoroughly reorganized the figures (**Figures 3, 5 and 6**), moved the exploratory datasets to EV/Appendix section, and retained only the key results in the main figures. These changes

should now improve the overall clarity of the manuscript. Some of the exploratory datasets from **Fig. 3** has been removed to improve the focus of the study. We believe that these changes have now streamlined the presentation and have improved the clarity and readability of the manuscript.

2. There is no direct demonstration of co-localization of WASH1, FAM21, and VPS35 simultaneously in control and TRIM62 KO cells. Given the partial co-localization shown for WASH1/FAM21 or WASH1/VPS35, it is important to demonstrate co-localization of all three proteins together. In addition, there is no evidence showing IAV co-localization with WASH1- or TRIM62-containing compartments. The statement in LN425-425 that TRIM62 interacts with WASH1 only via FAM21 is not sufficiently supported. Can TRIM62 directly interact with WASH1? Does KD of FAM21 disrupt the TRIM62-WASH association?

We thank the reviewer for suggesting these important points.

- In the initial submission, we demonstrated that TRIM62 depletion enhances the localization of WASH1 and FAM21 to EEA1-positive endosomes, whereas VPS35 localization remains largely unaffected. To quantify these effects, we measured the co-localization of WASH1, FAM21, and VPS35 with EEA1 and independently quantified the total intensity of each marker on EEA1-positive endosomes (Fig. 5A–D, Fig. 6I,J, and Fig. S6A,B in the original manuscript; corresponding to **Fig. 5A–C and Fig. EV4A–D** in the revised manuscript).

  To assess the association of WASH1, FAM21, and VPS35 within the same endosomal compartments, we employed super-resolution SIM with SIM^2 processing. To visualize all the three proteins simultaneously, we over-expressed FAM21-GFP in both control A549 and TRIM62 KD A549 cells and IIF was performed to detect endogenous WASH1 and VPS35. SIM imaging revealed that both FAM21-GFP and WASH1 exhibited increased colocalization with VPS35-positive puncta in TRIM62-depleted cells compared to control cells, supporting enhanced association of the WASH complex with VPS35-containing endosomal compartments upon TRIM62 loss. These data have now been incorporated into **Fig. 5D** of the revised manuscript and are discussed in the Results section.

  To check whether IAV colocalizes with WASH1/TRIM62, we first examined the expression of both GFP-tagged WASH1 and TRIM62. While TRIM62-GFP expression predominantly exhibited dispersed signals with some aggregates (cytoplasmic bodies), GFP-WASH1 showed both dispersed and punctate signals. Since dispersed signal of TRIM62 may not be ideal for checking colocalization with IAV with chances of false positive detection, we checked the colocalization between GFP-WASH1 and IAV. To this end, we labelled purified IAV particles with the lipophilic dye R18, and performed live cell imaging using Airyscan high-resolution microscope. We observed marked colocalization between fluorescently-labelled IAV and GFP-WASH1 during the early stage of viral entry. This data has now been incorporated into **Fig. 3G** and is discussed in the revised manuscript.

- Based on our observations we proposed that TRIM62 interacts with the WASH complex (not only WASH1) via FAM21. Through co-IP we found that the C-terminal PRY-SPRY domain of TRIM62 interacts with the N-terminal globular domain (1-356 aa) of FAM21, and Y2H assay indicated direct interaction between TRIM62 and

FAM21. In the revised manuscript we provide evidence of direct interactions between purified TRIM62 and FAM21 proteins with high to moderate affinity by MST. We also provide evidence that the PRY-SPRY of TRIM62 and FAM21 globular domain interact directly (**Fig. 6F**), supporting our microscopic and biochemical observations. Since in this study we specifically focused on regulation of VPS35-FAM21 interaction by TRIM62, we did not check if TRIM62 directly interacts with WASH1. Our SIM and biochemical data also suggest that TRIM62 negatively regulates VPS35-WASH complex association. Since FAM21 depletion affects WASH1 protein levels, we could not reliably determine if TRIM62-WASH1 interaction is impacted upon FAM21 silencing. Since neither TRIM62 nor VPS35 silencing had any effect on the levels of any of the WASH subunits, but altered their subcellular localization, we specifically investigated the VPS35-WASH complex interactions and localization in TRIM62 KD cells, and correlated the findings with IAV entry phenotypes.

3. The conclusions would be strengthened by performing concurrent deletion of TRIM62 with FAM21 or VPS35 and assessing restoration of the phenotype. Such epistasis experiments would help define pathway hierarchy.

We thank the reviewer for this insightful suggestion. To address this comment, we concurrently silenced FAM21 and VPS35 and observed marked increase in IAV entry as both these factors were found to exert antiviral defects. Interestingly, co-depletion of FAM21-VPS35 along with TRIM62 restored viral entry to the basal levels (**Fig. 5G**).

These epistasis experiments indicate that the entry defect observed upon TRIM62 depletion is due to the enhanced FAM21-VPS35 association. These findings suggest that TRIM62 functions upstream of the FAM21-VPS35 regulatory axis and support our model that TRIM62 promotes IAV entry by limiting WASH complex association with VPS35.

4. All experiments were performed in A549 cells. Validation in primary lung epithelial cells would substantially strengthen the physiological relevance of the findings.

We performed most of the experiments in A549 cells as this cell line is most widely used to study IAV infection, due to its lung origin and close physiological relevance. However, we also tested IAV infection upon TRIM62 depletion in other cell lines including HeLa, HepG2, and Huh-7 (**Fig. EV1 H-K**). In all of these cell lines, TRIM62 depletion significantly reduced IAV infection underscoring its broader role across different cell types. In addition, we also tested VPS35-FAM21 interaction in TRIM62-depleted HeLa cells by PLA, which showed similar phenotypes as A549 cells (**Appendix Fig. S2B**). In the revised manuscript, we have biochemically shown that TRIM62 limits the interaction of WASH1 with VPS35 in HEK293T cells (**Fig. EV4F and EV5C**), supporting the findings obtained from A549 and HeLa cells.

Following the reviewer's suggestion, we procured human bronchial epithelial cells (HBECs) to validate our findings in a more physiologically relevant cell type. Unfortunately, the cells were found to be contaminated upon receipt, preventing us from carrying out the planned experiments. Despite subsequent efforts to obtain a replacement batch, we were unable to secure fresh HBECs and complete the experiments within the revision timeframe.

5. TRIM62 protein expression in WT, KO, and complemented cells should be shown clearly by western blot and immunofluorescence to confirm knockout and rescue efficiency.

We have shown rescue of TRIM62 expression in TRIM62 KO cells complimented with WT-TRIM62-His by western blotting for **Fig. 1I** and the immunofluorescence images has been shown for **Fig. 6H**.

Due to the lack of commercially available antibody that can reliably detect endogenous TRIM62, we assessed the KD or knockout (KO) efficiency by qRT-PCR (**Fig. EV1A and EV1C)**. KO efficiency was further confirmed by DNA sequencing (**Fig. EV1B**). Similarly, to perform rescue experiments, TRIM62 KO cell lines were first confirmed by the above methods, following which we established rescue cell lines stably expressing TRIM62-His in the KO background by lentiviral system (**Methods Section**). Expression of TRIM62 in rescue cell line was then validated by western blotting and immunofluorescence (**Fig. 1I, Fig. 6H, Fig. EV5E**), depending on the context of the experiment.

6. In Fig 1G and 1I, NP expression appears reduced in IAV-infected TRIM62 KO cells, suggesting possible post-entry defects. Please include multicycle replication data using multiple IAV strains. In addition, include a control virus that is not affected by TRIM62 loss. Preferably, test a virus that enters exclusively via CME or macropinocytosis to distinguish entry-specific effects.

We agree that the decrease in NP intensity upon TRIM62 loss can be due to the observed cell entry defect or replication or both. But since we observed a primary block in viral entry, particularly at the endocytosis step, we focussed our study on viral entry. Viral plaque assay also showed marked reduction in viral titer in TRIM62 KO cells. Since we found that TRIM62 depletion blocks viral entry, multicycle replication assay can potentially confound the effect to determine whether TRIM62 is involved in viral entry alone, as entry defects can also lead to replication block.

We, therefore, devised an alternate strategy which can address more directly as to whether TRIM62 is involved in viral entry or replication or both with a more direct approach i.e. acid-induced plasma membrane (PM)-bypass assay. In this assay, we fused the virus at the PM by lowering the medium pH to 5.0 (the fusion pH for IAV at endosome), and probed for viral uncoating (30 min post-fusion) and NP expression (10 h post-fusion). While PM-bypass assay only mildly affected viral uncoating (**Fig. 2N**), indicating that TRIM62 specifically facilitate viral endocytic trafficking, reduction in NP expression by >60% indicated TRIM62's additional involvement in the post-entry events in IAV life cycle (**Fig. EV2F**). However, in this study, we specifically focused on TRIM62's role in the early entry of IAV.

In addition to IAV, we tested viruses using different entry pathways including CME (Semliki Forest virus, SFV), macropinocytosis (vaccinia virus, VV), or CME and non-CME (vesicular stomatitis virus, VSV). TRIM62 depletion significantly reduced SFV and VV infections, while whereas VSV infection was not significantly affected (**Fig. EV2G-I**). Since TRIM62 KD did not decrease CME or macropinocytic uptake of general bona fide cargoes transferrin or dextran, respectively (**Fig. 2A-C**), we reason that the observed decrease in viral infections is due to TRIM62's involvement in viral transcription/replication. However, future investigations will be required to interrogate the role of TRIM62 in post-entry steps.

7. In Fig 3 and LN 334-338, if KD of individual WAVE/WASH components destabilizes the entire complex, including WASH1 (Fig S5B), it is unclear why CTB uptake is not affected similarly to siWASH1 KD. This discrepancy requires clarification.

We agree with the reviewer that depletion of individual WASH components partially destabilizes the complex giving rise to IAV entry defects and similar should be expected for CTxB uptake. We have shown that depletion of both WASH1 or TRIM62 reduces CTxB uptake, and VPS35 KD leads to increase in CTxB uptake, similar to the observations in IAV entry. FAM21 KD also increased CTxB uptake, but the results were not statistically significant. However, the other WASH complex subunits CCDC53, SWIP or Strumpellin deficiency did not significantly affect CTxB uptake. These results suggest that although TRIM62, WASH1, and VPS35 regulate CTxB uptake in a manner similar to IAV entry, but the other WASH subunits are not involved, underscoring similar but not identical requirements. This has been discussed thoroughly in the Discussion section.

8. In Fig 5A and 5C, endosomes appear enlarged or altered in siTRIM62 cells compared to controls, suggesting broader defects in endosomal compartments. Could these structural changes contribute to altered IAV uptake? It is important to demonstrate that there are no global defects in viral replication in KO or KD cells by testing an unrelated virus.

We agree that altered endosome morphology (a population of early endosomes showing enlargement) is observed in TRIM62 KD cells. However, similar changes in endosomal morphology were also observed in VPS35 KD cells (**Fig. 5H** of revised manuscript), which in contrast to TRIM62 depletion, boosted IAV entry. Moreover, in double depletion of TRIM62 and VPS35, while IAV endocytosis is rescued, early endosomal enlargement still persisted (**Fig. 5H** of revised manuscript). These contrasting phenotypes in IAV entry, but similar endosomal morphology observed in TRIM62 or VPS35 KD suggests that IAV entry cannot be solely attributed to changes in endosomal morphology.

Also, as it was rightly suggested that the effect of TRIM62 depletion should be tested with additional viruses, we have now tested Semliki Forest virus (SFV), vaccinia virus (VV), and vesicular stomatitis virus (VSV) infection in TRIM62 KD cells. We found that TRIM62 depletion significantly reduced SFV and VV infection by approximately 30% and 39%, respectively, whereas VSV infection was largely unaffected (**Fig. EV2G-I**).

While these findings indicate that TRIM62 can influence viral infections across multiple viruses, these defects could arise from impaired entry or replication or both. Therefore, to specifically address whether the observed IAV uptake phenotype was due to broader disruption of the endosomal network, we examined the uptake of multiple endocytic cargoes (**Fig. 3A-D**). Notably, TRIM62 depletion selectively impaired CTxB uptake, whereas the internalization of transferrin, dextran, and EGF remained largely unimpaired. Since these cargoes rely on distinct endocytic pathways, the selective defect in CTxB uptake argues against a global perturbation of endosomal function and instead supports a more specific role for TRIM62 in regulating the pathway exploited by both CTxB and IAV.

9. Previous studies suggest that CPZ does not significantly affect IAV infection. Therefore, the strong inhibition of IAV endocytosis by CPZ in this study is surprising. The CPZ concentrations appear higher than in prior reports. Please include cell viability data for all siRNA KD and inhibitor treatments to rule out nonspecific toxicity.

In our study, chlorpromazine (Cpz) was used as a control exclusively for the viral endocytosis assay and not for infection assay measuring NP expression. Importantly, in our experiments, Cpz treatment was limited to a total duration of only 40 min (10 min pretreatment followed by 30 min during viral endocytosis). This relatively short exposure period was sufficient to inhibit viral uptake while not causing any appreciable adverse effects on cell viability. Therefore, we believe that the robust inhibition of endocytosis observed in our study

reflects a specific effect of Cpz on the virus entry process rather than nonspecific cytotoxicity. Cpz (Merck, C8138) was used at a concentration of 25 µg/ml for the IAV (H3N2) endocytosis assay in A549 cells. At this concentration, we did not observe any measurable cytotoxicity, as assessed by cell number in Cpz-treated cultures. A 10-min pretreatment followed by continuous presence of Cpz during virus uptake resulted in approximately 75% inhibition of IAV endocytosis.

The reported effects of Cpz on influenza virus infection vary across studies. For example, Sieczkarski and Whittaker (PMID: 12239322) used 10 µg/ml Cpz in HeLa cells and observed a 59% reduction in H1N1 infection. Importantly, they noted that prolonged exposure, including an additional 1.5 h treatment during virus binding, resulted in cellular toxicity. Similarly, Rossman et al. (PMID: 22875971) reported greater than 60% inhibition of H1N1 infection in MDCK cells following a 1-h pretreatment and an additional 1-h treatment during virus binding. In contrast, Sun et al. (PMID: 15936793) did not observe a significant effect of Cpz on influenza A/WSN infection, although the exact treatment duration was not specified.

Tables for referee with unpublished data have been removed upon request by the authors.

Minor Concerns:

1. What are the predicted off-target effects of the TRIM62 sgRNA?

The predicted off-target scores for the TRIM62 gRNA were **96** (CFD specificity score; Doench et al., *Nature Biotechnology*, 2016) and **93** (MIT specificity score; Hsu et al., *Nature Biotechnology*, 2013), indicating **high specificity with a very low probability of off-target effects**. These scores were obtained using CRISPOR.

2. Include average NP expression values in Fig 1A and 1C.

Thanks for this suggestion. We have now included average NP expression/intensity values in **Fig. 1A** and **Fig. 1C** of the revised manuscript.

3. For LN 208-210, visualization of M1 uncoating in TRIM62 KO complemented cells would improve clarity.

We realize that the data discussed in LN 208-210 of the initial manuscript might have caused confusion. LN 208-210 of the initial manuscript indicated the M1 dispersal intensity in A549 cells stably expressing TRIM62-Myc and MBP-Myc. This data does not indicate complementation in TRIM62 KO cells.

Since this data seemed ambiguous, we have now removed this data from the revised manuscript. Moreover, same conclusion is supported by the data shown in **Fig. 4G**. In **Fig. 4G**, MBP-His and TRIM62-His were transiently expressed in A549 cells and M1 (dispersed) intensity in the cells positive for His-expression were calculated.

4. LN 225-228: The DENV data in Fig S4A are not statistically significant; the text should reflect this.

We thank the reviewer for pointing out this. We initially included DENV infection data, where HepG2 cells were used. However, since most of our experiments were carried out in A549 cells, we tested additional viruses including SFV, VV, and VSV in A549 cells (**Fig. EV2G-I**) and replaced the DENV data for uniformity.

5. Fig 3 should be streamlined to highlight the most relevant data, with remaining CTB experiments moved to the supplementary section.

We thank the reviewer for this valuable suggestion. In response, we have moved the majority of the CTxB-related data (previously presented in Fig. 3) to **Fig. EV3** and have revised the corresponding interpretations in the main text to ensure they remain appropriately balanced and focused. In addition, we have removed some exploratory experiments (Fig. 3H, 3R-S and supplementary Fig. S4A-C) to streamline the presentation and sharpen the overall focus of the study.

6. Using consistent color coding for specific molecules within figures would improve readability.

Thanks for pointing out this discrepancy in the figures. We have now used consistent color coding for specific proteins/molecules in all the figures.

7. The dual siRNA KD experiment is difficult to interpret. Are both targets knocked down in the same cells?

For the KD experiments, both targets were depleted simultaneously by co-transfecting the corresponding siRNAs into the same cell population. To verify the efficiency of double KD, we assessed the depletion of each target independently and have now included these validation data in **Appendix Fig. S3**. In WASH1+VPS35 double KD cells, WASH1 and VPS35 signal intensities were reduced by 91% and 98%, respectively. Because simultaneous

visualization of both target proteins together with viral markers during IAV endocytosis and M1 uncoating is technically challenging, the knockdown validation experiments were performed separately from the viral trafficking assays. Similarly, in TRIM62+VPS35 double-KD cells, VPS35 staining was reduced by 95%, while qRT-PCR analysis revealed an approximately 80% reduction in TRIM62 transcript levels. Collectively, these data confirm efficient depletion of both targets under the double KD conditions. Accordingly, viral endocytosis and uncoating were quantified across the corresponding double KD cell populations.

8. In Fig 6G-I, it is unclear how the authors conclude that VPS35 intensity is unchanged in TRIM62 KO cells. Quantification and representative images should better support this statement.

We checked total protein levels of VPS35 by western blotting in TRIM62 KO/KD cells (**Appendix Fig. S2D and Fig. 5F**) and observed no significant changes upon TRIM62 depletion. Moreover, we have performed immunofluorescence with anti-VPS35 antibody in TRIM62 KD cells and found VPS35 intensity comparable in control and TRIM62-depleted cells. As the reviewer suggested to support these findings with immunofluorescence images, we have now shown the images in **Fig. EV4D** which were used to quantify the data shown in **Fig. 5B,C** of the revised manuscript. Moreover, together, immunofluorescence and western blotting both support our findings that VPS35 levels were not changed in TRIM62 deficient cells.

9. In Fig 5N and 5O, the legend states that 70 or 150 cells were analyzed; however, the panels appear to show fewer samples. Please clarify.

We thank the reviewer for carefully examining the manuscript and pointing out this apparent discrepancy. For Fig. 5N of the initial manuscript, we included data from 3 different experiments, but quantification was done from individual image fields containing 8-10 cells. Each image field was represented as a dot, and therefore the total number of cells quantified was ≥ 70 cells per sample (including all 3 experiments). To avoid this confusion, we have now clubbed all the image fields from each experiment and individual experimental data (clubbed) is shown as dots in each bar.

For Fig. 5O of initial manuscript, we have mentioned 1500 cells per sample in the figure legend. IAV endocytosis was calculated from 1500 cells per siRNA treatment as the data for this experiment is acquired from high-content confocal system.

Referee #3

General comments:
In this manuscript Gupta et al. use siRNA knockdowns to identify TRIM62 as a positive regulator of IAV infection. They use imaging-based virus entry assays to demonstrate a defect in virus uptake, then use model cargo proteins to more broadly investigate the role of TRIM62 in endocytosis. Their subsequent examination of actin regulating complexes leads to the observation that WASH is also important for IAV and CTxB uptake, and they suggest that WASH may be exerting a function at the plasma membrane. They then dissect the function of each WASH subunit in IAV entry, identifying FAM21 as functionally distinct from the others. Finally, they investigate the relationship between WASH, VPS35 and TRIM62, concluding that TRIM62 interacts with FAM21 and negatively regulates the FAM21-VPS35 interaction.

A particular strength of this manuscript is the IAV infection experiments, which are performed to a high standard and presented well. However, data reporting to establish the mechanism of TRIM62's direct regulation of FAM21's association with retromer, and the functional role for FAM21/retromer at cell surface lipid rafts are insufficient to support the core conclusions of the manuscript. A significant amount of additional work will be required to validate these key conclusions.

We thank the reviewer for the thorough evaluation of our manuscript. The reviewer has highlighted the strengths of the study and raised constructive concerns regarding certain findings. We are grateful for these valuable suggestions, which have been instrumental in improving the mechanistic rigor of the work. We have addressed all major and minor concerns point by point.

Specific points:
As WASH and retromer are involved in endosomal protein sorting and recycling, disruption of either of these complexes has a downstream impact on the cell surface levels of hundreds of different proteins. The authors must include a discussion of how their WASH/FAM21/VPS35/TRIM62 knockdowns may affect the protein composition of the cell surface and whether such a global cellular reconfiguration could contribute to the observed endocytosis phenotypes. Retromer perturbation also causes altered RAB7 activity and induces cellular stress, autophagic and lysosomal, the latter likely affecting HA acidification and M1 uncoating independently of WASH. The authors should also discuss these confounding factor in the manuscript and how it impacts on their data interpretation.

We agree that perturbation of the WASH and retromer complexes can have broad effects on endosomal protein sorting, cargo recycling, and cellular homeostasis, which could potentially influence viral entry through indirect mechanisms. To address this concern, we examined the uptake of several endocytic cargos in TRIM62 KD cells. We found no detectable uptake defects for transferrin, EGF, or dextran (**Fig. 3A-C**), indicating that clathrin-mediated endocytosis, receptor-mediated uptake, and fluid-phase endocytosis remain largely unimpaired under these conditions. In contrast, we observed a significant reduction in CTxB uptake, suggesting that TRIM62 depletion selectively affects specific endocytic trafficking pathways rather than causing a global impairment of endocytosis.

In addition, we investigated the localization of GLUT1, a well-established cargo whose trafficking is regulated by the WASH machinery. This data is provided in **Appendix Fig. S2E**. We found that, whereas GLUT1 predominantly localizes to the plasma membrane in control cells, it becomes redistributed to the Golgi region in TRIM62 KD cells. This altered localization is consistent with defects in endosomal cargo sorting and recycling and provides independent evidence that TRIM62 depletion affects trafficking functions of WASH-dependent cargoes.

We agree that perturbation of WASH-retromer function may also have broader consequences for RAB7 regulation, autophagic and lysosomal pathways, and the composition of the cell surface proteome. While a comprehensive analysis of these processes is beyond the scope of the present study, we have now expanded the Discussion section to address these possibilities and their potential implications for interpretation of the viral entry phenotypes. Importantly, the selective trafficking defects observed in TRIM62 KD cells, together with the biochemical and imaging evidence presented in this study, support a model in which TRIM62

regulates WASH-retromer-dependent endosomal trafficking rather than causing a generalized disruption of endocytic function.

The authors conclude that the WASH complex and retromer are enriched at the leading edge in VPS35 suppressed cells. In the imaging data (e.g. Figure 3), only the highlighted cell is showing any 'enrichment' in WASH signal (Fig. 3S). Typically, one would expect to observe enrichment of the cell surface signal at the cell:cell boundaries where two cell surface membranes are juxtaposed. How do the authors exclude an alternative explanation: enrichment of cytosolic WASH1 at 'leading edge protrusions' is a simple consequence of more cytoplasm being present within this highly folded cytosol:membrane region and not a consequence of a specific enrichment at the cell surface.

We agree with the reviewer that increased signal intensity at cellular protrusions could, in principle, arise from local increases in cytoplasmic volume rather than specific protein enrichment. We would like to clarify that we did not aim to conclude that WASH or retromer are specifically enriched at cell-cell junctions or uniformly at the plasma membrane. Rather, our observations indicate that a subpopulation of WASH and retromer localizes to peripheral VPS35-positive structures, some of which are situated close to the plasma membrane. Importantly, our central conclusion does not rely solely on the apparent redistribution of WASH signal at the cell periphery.

The main observations of this study through super-resolution SIM indicated colocalization of WASH subunits with VPS35, and TRIM62 depletion resulted in increased accumulation of WASH proteins at VPS35-positive spots (**Fig. 5D**). These structures were most prominently observed on endosomal compartments rather than at cell-cell boundaries. Therefore, to avoid overinterpretation, we have revised the text to place less emphasis on peripheral enrichment and instead focus on the increased association of WASH subunits with VPS35-positive spots observed upon TRIM62 depletion, which is supported by both microscopy and biochemical data. Interestingly, in a new experiment using SIM imaging, we observed that a pool of WASH1 becomes enriched at the plasma membrane upon FAM21 depletion (**Fig. 4J**). Whether this plasma membrane–enriched WASH1 plays a specific role in viral entry remains to be investigated.

Extending this, (see line 289-291 and Fig. 3O and 3R), compared to the CTxB signal the VPS35 + FAM21 PLA signal is juxtaposed to the cell surface and does not show a relationship with CTxB signal intensity. High-resolution EM is required to make the conclusion that 'WASH and retromer associate within lipid raft-enriched domains at the plasma membrane'. Otherwise, these signals could simply reflect endosomes positioned close to the plasma membrane, which is a known feature of this network.

We thank the reviewer for this important point. We agree that the PLA signals observed for VPS35–FAM21 association do not definitively demonstrate localization within lipid raft–enriched domains at the plasma membrane. Given that only a small fraction of VPS35–FAM21–positive puncta were detected at the plasma membrane, these signals may instead originate from peripheral endosomal structures closely apposed to the plasma membrane, which is fully consistent with the established distribution of the WASH–retromer machinery. Accordingly, in the revised manuscript we have toned down our interpretation regarding VPS35–FAM21 association at CTxB-positive plasma membrane domains in both the Results and Discussion sections and replaced the plasma membrane PLA data with more important results. In the revised manuscript, we included data from super-resolution microscopy showing

increased colocalization of FAM21 and WASH1 with VPS35-positive puncta upon TRIM62 depletion.

Together with our biochemical data demonstrating a direct interaction between TRIM62 and FAM21 and a reduction in WASH-VPS35 association upon TRIM62 overexpression, these findings support a role for TRIM62 in regulating WASH-retromer coupling. We believe these conclusions are well supported by the available data and are not dependent on whether the relevant VPS35-positive structures are located immediately beneath the plasma membrane or on peripheral endosomes.

The direct association of TRIM62 with FAM21 requires further experimental validation. The Y2H analysis, in a background of some self-activation, is insufficient to conclude the direct nature of this interaction. This is a critical conclusion of the manuscript and hence needs robust validation The authors have provided evidence to map this potential interaction to the PRY-SPRY module of TRIM62 and the N-terminal region of FAM21. Does AlphaFold predict a high confidence score for this potential binding? The authors should certainly isolate these regions as recombinant proteins and perform biochemical assays to establish the direct nature of the binding.

We thank the reviewer for highlighting the importance of validating the direct interaction between TRIM62 and FAM21. We agree that the Y2H data alone are insufficient to conclusively establish direct binding, particularly given the potential for self-activation artifacts. Therefore, in the revised manuscript, we have complemented our Y2H and co-immunoprecipitation analyses with biochemical studies using purified recombinant proteins.

Specifically, based on our domain-mapping experiments, which identified the C-terminal PRY-SPRY domain of TRIM62 and the N-terminal globular domain of FAM21 as the interacting regions, we purified these domains and examined their interaction using Microscale Thermophoresis (MST) (**Fig. 6F**). These experiments demonstrated direct binding between the purified proteins, providing biochemical evidence that the interaction does not require additional cellular factors. Furthermore, the interaction detected by MST is fully consistent with our co-immunoprecipitation data obtained using both full-length and domain-truncated constructs, which independently mapped the interaction to the same regions of TRIM62 and FAM21.

We appreciate the reviewer's suggestion regarding AlphaFold-based structural predictions. We initially performed in silico modeling using the AlphaFold server; however, we observed only moderate interaction confidence scores, which could be attributed to the extensive intrinsically disordered regions within FAM21 that can limit prediction accuracy. Despite this limitation, the AlphaFold model did predict a potential interaction between the C-terminal PRY-SPRY domain of TRIM62 and the N-terminal globular region of FAM21, which is fully consistent with our experimental findings. In the revised manuscript, we have further strengthened this conclusion by including MST experiments using purified recombinant proteins, which directly demonstrated binding between TRIM62 and FAM21. While high-resolution structural modeling could provide additional mechanistic insight, we believe that the combined evidence from domain mapping, co-IP, Y2H analysis, and direct biophysical binding measurements provides robust and comprehensive validation of a direct TRIM62-FAM21 interaction, supporting our conclusions in the present study.

If direct binding is observed, the authors need to consider how TRIM62 binding to the N-terminal region of FAM21 prevents retromer binding to the R19-R20 and R21 LFa repeats at the unstructured C-terminal tail of FAM21.

Our data demonstrate that TRIM62 directly interacts with the N-terminal globular domain of FAM21 and that modulation of TRIM62 levels alters WASH-retromer association. However, the molecular mechanism by which this interaction affects FAM21-retromer binding remains unresolved. Addressing whether TRIM62 induces conformational changes in FAM21, affects accessibility of the LFa repeats, or regulates the interaction through an indirect mechanism would require substantial additional structural and mechanistic studies.

In light of the editor's guidance and the scope of the current study, we have not pursued further investigation of this mechanism. Instead, we focused on establishing the direct TRIM62-FAM21 interaction and its functional consequences for WASH-retromer association and IAV entry. We agree that elucidating the molecular basis of this regulation represents an important direction for future studies.

Minor points:

In all figure legends please indicate which statistical test(s) were used, and in all graphs please indicate where differences are not statistically significant.

We thank the reviewer for this suggestion. In the revised manuscript, we have updated all figure legends to explicitly indicate the statistical tests used for each analysis. In addition, we have revised the graphs to denote comparisons that did not reach statistical significance by labeling them as "ns" (not significant), where appropriate.

Line 65: Include reference to Aitor Hierro's work - Romano-Moreno et al., Protein Sci 33:e4980 (2024).

We have now cited the work by Romano-Moreno *et al.* (Protein Science, 2024) in the Discussion section describing WASH-retromer complex organization and regulation.

Line 67-70: May want to include reference to Visweshwaran et al., EMBO J. 37:e97706 (2018) on the assembly of the WASH complex.

We have now cited the work by Visweshwaran *et al.* (EMBO J, 2018) in the revised manuscript.

Lines 173-178: A large number of experiments are discussed very briefly. Please describe the experiments more fully to improve clarity for the reader.

In the initial submission, we kept the descriptions of these experiments relatively concise to accommodate manuscript word limits. However, we agree that additional detail improves clarity for the reader. Accordingly, we have expanded the corresponding sections in the revised manuscript to provide a more complete description of the experimental design and key findings.

Lines 227-228: The result in Fig. S4A is not statistically significant, please address this fact in the text.

We thank the reviewer for pointing this out. In the initial submission, we had included DENV infection data generated in HepG2 cells. However, since the central mechanistic framework of this study, covering TRIM62, WASH, retromer, and IAV entry, was evaluated predominantly in A549 cells, we sought to maintain cellular consistency across the viral infection assays.

Accordingly, we examined the effect of TRIM62 depletion on additional viruses in the same A549 cellular background and examined SFV, VV, and VSV infection in this cell line (**Fig. EV2G-I**). These experiments allowed us to directly compare viral infection phenotypes within the same mechanistic context and cell type used for the core analyses. Therefore, we have replaced the previously included DENV (HepG2-based) data with these A549-based infection assays to ensure experimental consistency across the study.

Fig. 2N, Fig. 3B-E, Fig. 4G-H: Please show representative images used for the quantification in the main figure or supplement.

We have now included representative images corresponding to the quantifications shown in Fig. 3B-E, and Fig. 4G-H in the revised manuscript (**Fig. 3A-D** and **Fig. 4G-H**). For Fig. 2N, this data has been removed to avoid redundancy as similar conclusions were drawn from **Fig. 4G** of the revised manuscript.

Line 259-261. This is a bold statement given that the data are derived from KD experiments.

We thank the reviewer for this important comment. We would like to clarify that our analysis included both genetic and pharmacological perturbation of the Arp2/3 complex. We also employed CK-666, an inhibitor of Arp2/3 complex and found the similar results as we found with ACTR3 KD. However, we agree with the reviewer that these findings alone are not sufficient to definitively exclude a role for branched-actin in IAV entry. Therefore, we have moderated our conclusion in the revised manuscript.

Line 266-267. The WASH complex is also recruited to endosomal membrane independently of retromer (Simonetti et al., 2017; Kvainickas et al., 2017) through SWIP association with the endosomal phosphoinositide PI(3,5)P2 (Dostal et al., 2023). An additional, mechanism have been proposed for association with HRS (MacDonald et al., 2018). These points should also be added in the Introduction (line 62).

Following this suggestion, we have revised the Introduction to include the discussion of retromer-independent WASH recruitment pathways, including SWIP-mediated association with PI(3,5)P2-containing endosomal membranes (Dostal et al., 2023), as well as previously reported roles for HRS in WASH complex recruitment (MacDonald *et al.*, 2018). We have also incorporated the studies by Simonetti et al. (2017) and Kvainickas *et al.* (2017) to provide a more comprehensive overview of current models for WASH complex membrane association.

Line 377: 'renders WASH inactive' is a very strong statement. Where is the evidence to support this.

We agree that the phrase "renders WASH inactive" was too strong and not directly supported by the data presented in this study. While we observed increased accumulation of WASH on endosomal structures in TRIM62 KD cells, this was accompanied by reduced branched actin nucleation, suggesting that the endosome-associated WASH complex is

functionally impaired under these conditions. In addition, we now provide new data showing mislocalization of GLUT1 (**Appendix Fig. S2E**), a known WASH/retromer cargo, from the plasma membrane to the Golgi region in TRIM62 KD cells, further supporting defects in cargo trafficking. Nevertheless, because we did not directly measure WASH nucleation activity, we have revised the text to use more cautious language and refer to impaired WASH function rather than stating that WASH is rendered inactive.

Fig. 3R: This PLA assay requires appropriate negative controls, such as the EEA1/VPS35 control used in Fig. 6G.

We agree with the reviewer that appropriate controls are important for validating the PLA data. In Fig. 6H (**Fig. 5E** in the revised manuscript), we included a VPS35-EEA1 PLA control. The rationale for using this control is that although VPS35 localizes to EEA1-positive early endosomes, there is no reported interaction between VPS35 and EEA1. Therefore, this control allowed us to assess whether the VPS35-FAM21 PLA signals reflected specific nanoscale proximity rather than merely the co-residence of both proteins within the same endosomal compartment. Consistent with this rationale, VPS35-EEA1 PLA signals were near background levels. To further evaluate assay specificity, we additionally performed VPS35-FAM21 PLA in VPS35-depleted cells and observed a marked reduction in PLA signals compared with control cells, confirming that the detected PLA signals are specific and accurately reflect the close proximity of VPS35 and FAM21 in cells (**Appendix Fig. 2C**).

Fig. 5, 6: Please indicate in the figure legend how many times western blots were repeated, and whether results were consistent.

All the experiments reported have been done at least three times. This has now been indicated in each figure legend. All the interactions reported in this manuscript have been tested multiple times and are consistently obtained by co-IP assays.

Fig. 6L: In the final lane of this experiment there is low expression of the TRIM62 construct, and IP of the FAM21 357-1341 construct is weak. There is also no indication that this experiment has been repeated. Therefore, the conclusion that FAM21 head deletion disrupts the TRIM62-FAM21 interaction is not robustly supported by the data.

We agree that the reduced expression of TRIM62 and the weaker recovery of the FAM21(357-1341) construct in the final lane make the interpretation of this co-IP experiment less straightforward. We would like to clarify that this experiment was repeated three independent times and, in all cases, we consistently observed reduced TRIM62 expression levels in the presence of the FAM21(357-1341) construct. The similar observations were previously found with FAM21 tail (357-1341) in the original study characterizing its interactions with WASH1 (Gomez *et al*., Dev. Cell, 2018).

To further address this concern, we performed an independent biophysical binding assay using purified recombinant FAM21(1–356) and TRIM62. MST analysis demonstrated direct binding between TRIM62 and the FAM21 head domain, with a dissociation constant (K_D) in the nanomolar range, indicating a high-affinity interaction (**Fig. 6F**). These data provide independent support for our conclusion that the FAM21 head region mediates interaction with TRIM62.

Fig. S5A: Please address the result that siWASH1 #1 has no effect on IAV infection in the text.

We used two siRNAs per gene for IAV infection assay (Previously Fig. S5A, now **Appendix Fig. S1B**) and observed similar phenotypes for all genes except WASH1 for which only one siRNA showed the phenotype. When we checked transcript levels, we found that the WASH1 siRNA which had no effect also did not efficiently supressed WASH1 transcript level. However, to avoid confusion, we have now included data only from validated siRNAs in the revised manuscript.

Fig. S8G: It is unclear whether the re-expressed TRIM62 constructs all express to comparable levels, which is important for interpreting these data. Please include western blots of the rescue cell lysates to address this.

We agree that the expression levels of the different TRIM62 constructs should be considered when interpreting the rescue experiments. We would like to clarify that all rescue experiments were performed in TRIM62 KO cells stably expressing full-length TRIM62 or its mutants, thereby minimizing artefacts associated with transient overexpression. In the independently generated stable cell lines, the expression levels of the different constructs were not identical and also displayed cell-to-cell variability within each population, possibly due to the intrinsic property of TRIM62 and its domains. To ensure that the rescue phenotype was assessed specifically in cells expressing the respective TRIM62 construct, we performed immunofluorescence co-staining for the His tag and IAV M1, and quantified M1 dispersal only in His-positive cells only. We believe that this single-cell approach provides a more direct assessment of the relationship between TRIM62 mutant expression and IAV entry than population-averaged measurements alone, due to differences in protein expression levels.

To make this point clearer, we have now included representative immunofluorescence images showing expression of all TRIM62 mutants together with the corresponding M1 staining in **Fig. 6H** and **Fig. EV5E**, and have clarified this in the revised manuscript.

In siTRIM62 treated cells the early endosomal compartment appears to partially collapse into the perinuclear region. How do the authors disentangle this change in steady state distribution with the increased Mander's correlation coefficient?

We appreciate the reviewer's detailed observation and agree that the endosomal network in TRIM62-depleted cells appears more condensed in the perinuclear region, which could potentially contribute to an increased Mander's correlation coefficient (MCC) between FAM21/WASH1 and EEA1. However, if the elevated MCC values were solely a consequence of endosomal aggregation, a similar increase would be expected for VPS35, given its shared localization on endosomes. Notably, we did not observe any appreciable difference in the MCC between VPS35 and EEA1 in control and TRIM62-depleted cells. In contrast, increased MCC values were observed specifically for the WASH subunits FAM21 and WASH1. These findings suggest that the observed increase in WASH complex association with endosomes cannot be fully explained by altered endosomal morphology alone and instead support a specific role for TRIM62 in regulating the recruitment or retention of WASH complex components on endosomes.

1st Revision - Editorial Decision 29th Jul 2026

Dear Dr. Banerjee

Thank you for the submission of your revised manuscript to EMBO reports. We have now received the full set of referee reports that is copied below.

As you can see, all three referees find that the study has been significantly improved during revision. Referee #3 however points out remaining concerns regarding the reported interactions between FAM21, VPS35, WASH, and TRIM26. After having seen all reports, referee #2 agreed with these issues. I copy the further feedback from referee #2 alongside the reports.

Therefore, please tone down the conclusions in the title, abstract and manuscript accordingly and discuss limitations along the lines suggested by the referees. Please also provide a point-by-point response to the remaining concerns from referee #3 and to the feedback from referee #2.

From the editorial side, there are also a few things that we need before we can proceed with the official acceptance of your study.

- The Data availability statement should exclusively refer to data that have been deposited in public repositories. In case this doesn't apply, please use this text (only): "This study includes no data deposited in external repositories".

- The 'Disclosure and competing interests statement' needs to be placed after the Acknowledgments.

- Regarding the Author Contributions, we use CRediT to specify the contributions of each author in the journal submission system. CRediT replaces the author contribution section, which therefore needs to be removed from the manuscript text. You can use the free text box in our system if you wish to provide more detailed descriptions. See also guide to authors https://www.embopress.org/page/journal/14693178/authorguide#authorshipguidelines.

- Work with viruses: please provide a biosafety statement and state whether the work was performed in a BSL-2 laboratory and approved.

- Please provide a complete author checklist, which you can download from our author guidelines. Please insert information in the checklist that is also reflected in the manuscript. The completed author checklist will also be part of the RPF.

- The information on funding in the manuscript and the online submission system must be congruent. Please enter the following funders in the system: the Department of Science and Technology, Govt. of India (FIST grant no. SR/FST/LS-II/2017/97 to the Department of Biological Sciences, IISER Mohali), and IISER Mohali. Please double-check and make sure that all funders listed in the manuscript are also entered separately in our system.

- Appendix: please remove the author information from the title page. The title page should only have "Appendix to [manuscript title]" and the table of content.

- Appendix Figures: pleasae define the exact p-values in all legends that describe quantification and statistical analysis.
- Appendix Figure S1A/B, S3: please define the number of repeats (technical/independent).

- Please remove the Reagents and Tools table (Appendix Table S1) from the Appendix and provide it as .docx file. The template can be downloaded from our Guide to Authors. The table is then uploaded as separate file, file type Reagents table. The nomenclature for callouts is "Reagents and Tools table".

- The scale bars in Figure EV1D, H, L are hardly visible if one looks at the figure without zooming in.

- Figure EV3A, B: please define the size of the scale bar only in the legend, not in the figure panel. EV3G, H need scale bars.

- Please add scale bars for Figure EV4H.

- Please address the following comments in the figure legends and use either track changes or highlights:
* Please provide the exact p values in the legends of figures 1E, J; 2C, D, H, I, K; 3B, F; 4F, H; 6A, E, G; 5G, 6A, C, I, J; EV1 B-F, G, H; EV2 H; S1 A, B; S2 E.
* Please indicate the statistical test used for data analysis in the legends of figures EV1 B, D
* Please define the box plots in terms of minima, maxima, centre, bounds of box and whiskers, and percentile in the legends of figures EV2A, B, C, D, E, F; EV4 A, B, C, G, H; S2 E
* Please define the error bars in the legends of figures S1 A, B; S3.
* Please define the yellow arrow heads in the legend of figure EV4 H.

- We perform a spot check of all microscopy images provided and I noticed the following. These considerations apply of course to all imaging data:

* Comparing the source data for Figure 1H with the figure panel, it appears as if the contrast and/or exposure was modified quite strongly, as the cytoplasmic background seen in the source data (NP) is hardly visible in the panel. Please reduce the contrast modification and display the original images.

* Source data for Figure 4H: the FAM21-GFP_GFP image appears black. The signal can be seen if opened with Fiji but not with Preview or Photoshop, in contrast to the other images in this folder. As not everyone would try with different programs, please check this image.
(* BTW: the nucleus in Figure 4J, siFAM21 looks like a heart. Have you noticed?)

- Please provide the synopsis image in PNG or JPG format at a size of 550x300 pixels (width x height). Please upload the image with this size and make sure that all text is legible.

- Please remove the synopsis text from the manuscript and upload it as separate .docx file.

I look forward to seeing a new revised version of your manuscript as soon as possible.

Kind regards,

Martina Rembold, PhD
Senior Editor
EMBO reports

==================

Referee #1:

In this revised manuscript, the authors have addressed all of my prior concerns well. I do not have any additional comments.

Referee #2:

I have read the revised manuscript from Sharma and colleagues along with their detailed point-by-point response. This is an immense amount of work that identifies several unique aspects as it pertains to the regulation of WASH complex activity and connectivity to Retromer, along with IAV endocytosis. The have sufficiently responded to my critique adding in additional experiments where needed. They have also significantly reorganized the manuscript and added in the detailed information that was requested by the reviewers. This is a much improved manuscript.

Referee #3:

The authors have addressed most of those comments made during our previous review of this manuscript. Two key outstanding data remain unclear and these form the focus of what the authors highlight as the major point of their study: "The primary focus of this work was to define the role of TRIM62 in regulating WASH-retromer association and the impact of this regulation on IAV entry".

The authors sort to understand how TRIM62 binding to FAM21 affects retromer association through GFP-nanotrap IPs of expressed VPS35-GFP. They state that under their conditions they were unable to detect VPS35 binding to endogenous FAM21, an interaction that numerous other labs have validated and defined by IP approaches, but they were able to detect endogenous WASH1 binding (i.e. FAM21 independent binding of WASH1 to VPS35). This raises some concern as to what they are examining in these assays and the inference of the effect of TRIM62 on the FAM21-VPS35 axis is therefore indirect.

The authors have also tried to provide additional data of the direct nature of the TRIM62 binding to FAM21. They show a very low affinity association between the recombinant PRY-SPRY domain of TRIM62 and the amino-terminal region of FAM21. As I still have reservations as to the validity of this interaction, I think two important points should be made clear in their manuscript. First, AlphaFold modelling fails to predict with any confidence the molecular details of this potential association. Secondly, the authors must make it clear that this region of FAM21 is normally buried within the global head region of the functional pentameric WASH complex. Without evidence that TRIM62 can associate with FAM21 when it is bound to strumpellin, CCDC53, WASH and SWIP, the conclusion that TRIM62 can directly bind to the entirely WASH complex remains to be established.

Collectively, the authors conclusion of the FAM21-VPS35 axis as a 'molecular checkpoint' is, in my opinion, remains an over-interpretation of their data. The title and abstract should be edited to better reflect these mechanistic limitations.

================

Feedback from Referee #2 on the report from Referee #3:

It seems that many of the remaining issues can be addressed through changing the title, abstract, softening their conclusions and adding appropriate text to the Discussion. The FAM21 co-IP with VPS35 does require specific lysis buffers that favor the association. It's likely they did not use any of these. I agree that TRIM62 SPRY domain binding to the FAM21 globular head could be an issue. However, since the structure of this domain along with the other 4 components of the WASH complex is unknown, there could be a surface-exposed FAM21 element that mediates the interaction. Regardless, this needs to be discussed in the context of the manuscript as pointed out by reviewer 3.

Response to the Editor's and Reviewers' comments

Comments from the Editor

- The Data availability statement should exclusively refer to data that have been deposited in public repositories. In case this doesn't apply, please use this text (only): "This study includes no data deposited in external repositories".

We have revised the data availability statement in the manuscript. As no datasets generated in this study were deposited in external repositories, the statement now reads: "This study includes no data deposited in external repositories" in the revised manuscript.

- The 'Disclosure and competing interests statement' needs to be placed after the Acknowledgments.

We have now placed this statement after Acknowledgements in the revised manuscript.

- Regarding the Author Contributions, we use CRediT to specify the contributions of each author in the journal submission system. CRediT replaces the author contribution section, which therefore needs to be removed from the manuscript text. You can use the free text box in our system if you wish to provide more detailed descriptions. See also guide to authors https://www.embopress.org/page/journal/14693178/authorguide#authorshipguidelines.

We have removed the Author Contributions section from the manuscript text, in accordance with the journal's guidelines. The authors' contributions have been specified using CRediT in the journal submission system.

- Work with viruses: please provide a biosafety statement and state whether the work was performed in a BSL-2 laboratory and approved.

Yes. All experiments involving infectious viruses were conducted in a BSL-2 facility with the appropriate institutional biosafety approval. We have now mentioned this in the Methods section under the subheading "Viruses, bacteria, and yeast."

- Please provide a complete author checklist, which you can download from our author guidelines. Please insert information in the checklist that is also reflected in the manuscript. The completed author checklist will also be part of the RPF.

We have completed the Author Checklist and included all the information asked. The completed checklist has been uploaded with the revised submission.

- The information on funding in the manuscript and the online submission system must be congruent. Please enter the following funders in the system: the Department of Science and Technology, Govt. of India (FIST grant no. SR/FST/LS-II/2017/97 to the Department of Biological Sciences, IISER Mohali), and IISER Mohali. Please double-check and make sure that all funders listed in the manuscript are also entered separately in our system.

We have revised the funding information in the online submission system to ensure that it is fully consistent with the manuscript.

- Appendix: please remove the author information from the title page. The title page should only have "Appendix to [manuscript title]" and the table of content.

We have revised the Appendix accordingly. The author information has been removed from the title page, which now contains only "Appendix to TRIM62 facilitates influenza A virus entry by regulating WASH-dependent endosomal trafficking" and the Table of Contents, in accordance with the journal's guidelines.

- Appendix Figures: pleasae define the exact p-values in all legends that describe quantification and statistical analysis.

We have revised the legends of all Appendix Figures containing quantification and statistical analyses to report the exact *P*-values. However, we have not reported *P*-values in the Figure legends of 1A, 1C, 4A-D as these figures represent RNAi screens (heatmaps).

- Appendix Figure S1A/B, S3: please define the number of repeats (technical/independent).

We have revised the legends of Appendix Figures S1A/B and S3 to specify the number and type of experimental repeats. The legends now clearly indicate that the data represent independent biological experiments.

- Please remove the Reagents and Tools table (Appendix Table S1) from the Appendix and provide it as .docx file. The template can be downloaded from our Guide to Authors. The table is then uploaded as separate file, file type Reagents table. The nomenclature for callouts is "Reagents and Tools table".

We have removed Appendix Table S1 from the Appendix and prepared the Reagents and Tools table as a separate .docx file using the journal's template. The file has been uploaded separately under the file type "Reagents table".

- The scale bars in Figure EV1D, H, L are hardly visible if one looks at the figure without zooming in.

We have revised Figure EV1D, H, and L by replacing the original scale bars with more prominent scale bars that are readily visible.

- Figure EV3A, B: please define the size of the scale bar only in the legend, not in the figure panel. EV3G, H need scale bars.

We have revised Figure EV3 accordingly. The scale bar sizes have been removed from Figure EV3A and EV3B and are now specified only in the figure legend. In addition, scale bars have been added **to** Figure EV3G and EV3H.

- Please add scale bars for Figure EV4H.

We have added the scale bar to Figure EV4H and updated the figure accordingly.

- Please address the following comments in the figure legends and use either track changes or highlights:
** Please provide the exact p values in the legends of figures 1E, J; 2C, D, H, I, K; 3B, F; 4F, H; 6A, E, G; 5G, 6A, C, I, J; EV1 B-F, G, H; EV2 H; S1 A, B; S2 E.*

We have revised the figure legends for Figures 1E, J; 2C, D, H, I, K; 3B, F; 4F, H; 5G; 6A, C, E, G, I, J; EV1B–H; EV2H; S1A, B; and S2E to include the exact P values wherever available. For analyses performed using one-way ANOVA with Dunnett's multiple comparisons test, GraphPad Prism (version 9) reports extremely small adjusted P values only as "$P < 0.0001$" and does not provide the corresponding numerical values. Accordingly, these comparisons remain reported as "P < 0.0001", while all other exact P values have been included as suggested.

** Please indicate the statistical test used for data analysis in the legends of figures EV1 B, D*

EV1B figure shows TRIM62 KO validation by showing DNA sequence downstream of the gRNA-targeted genomic region from WT and TRIM62 KO A549 cells. DNA sequences from WT and TRIM62 KO are translated using Expasy Translate tool and pairwise aligned using EMBOSS Needle tool, indicating premature protein termination in the KO cells. EV1D represents high-content confocal images of IAV infection in control and TRIM62 KD A549 cells infected with WSN, NYMC, and Udorn for 10 h. None of the data report any quantified data and thus no statistical analysis have been employed.

** Please define the box plots in terms of minima, maxima, centre, bounds of box and whiskers, and percentile in the legends of figures EV2A, B, C, D, E, F; EV4 A, B, C, G, H; S2 E*

We have revised the legends of Figures 1J, EV2A-E, EV4A-C, G, H, and S2E to explicitly define the box and whiskers plots. The legends now state that the centre line represents the median, the box boundaries correspond to the 25th and 75th percentiles (interquartile range), the whiskers extend to the minimum and maximum values, and that all individual data points are overlaid.

** Please define the error bars in the legends of figures S1 A, B; S3.*

We have revised the legends of Figures S1A, S1B, and S3 to define the error bars. The legends now state that the data are presented as mean ± SD and that the error bars represent SD.

** Please define the yellow arrow heads in the legend of figure EV4 H.*

We have revised the legend of Figure EV4H to define the yellow arrowheads. The legend now states that the yellow arrowheads indicate F-actin and/or ARPC5 localization on enlarged, Rab5-QL-positive endosomes**.**

- We perform a spot check of all microscopy images provided and I noticed the following. These considerations apply of course to all imaging data:

** Comparing the source data for Figure 1H with the figure panel, it appears as if the contrast and/or exposure was modified quite strongly, as the cytoplasmic background seen in*

the source data (NP) is hardly visible in the panel. Please reduce the contrast modification and display the original images.

We thank the Editor for this observation. The contrast and brightness of the images in Figure 1H were adjusted to improve the visualization of the NP signal. However, we agree that the adjustments were more pronounced than necessary and may have reduced the visibility of the cytoplasmic background. We have therefore reprocessed the images using more conservative contrast and brightness settings that better preserve the original signal distribution while maintaining clear visualization of the NP staining. The revised figure now more closely reflects the original images.

** Source data for Figure 4H: the FAM21-GFP_GFP image appears black. The signal can be seen if opened with Fiji but not with Preview or Photoshop, in contrast to the other images in this folder. As not everyone would try with different programs, please check this image.*
(BTW: the nucleus in Figure 4J, siFAM21 looks like a heart. Have you noticed?)*

We agree that the original FAM21-GFP image appears very dark. This is because FAM21 overexpression is highly cytotoxic in A549 cells, requiring us to keep its expression at a relatively low level to preserve cell viability while still allowing detection of the GFP signal. We therefore revisited the original imaging dataset and identified another field in which the FAM21-GFP signal, although still modest, is more clearly visible. We have now replaced the original image with this one in the revised Figure 4H.
(Figure 4J, siFAM21 image: Thank you for the keen observation, we had not noticed it before. Perhaps it symbolises the heart we put into this work!)

- Please provide the synopsis image in PNG or JPG format at a size of 550x300 pixels (width x height). Please upload the image with this size and make sure that all text is legible.

We have reformatted the synopsis image according to the journal's specifications. The revised synopsis image has been provided in JPG format at the required dimensions of 550 × 300 pixels, and the text has been adjusted to ensure that it is clearly legible.

- Please remove the synopsis text from the manuscript and upload it as separate .docx file.

We have removed the synopsis text from the manuscript and uploaded it as a separate word file, as suggested.

Comments from the Reviewers

Referee #1:

In this revised manuscript, the authors have addressed all of my prior concerns well. I do not have any additional comments.

We sincerely thank the reviewer for the careful re-evaluation of our revised manuscript and are pleased that the revisions have satisfactorily addressed the previous concerns.

Referee #2:

I have read the revised manuscript from Sharma and colleagues along with their detailed

point-by-point response. This is an immense amount of work that identifies several unique aspects as it pertains to the regulation of WASH complex activity and connectivity to Retromer, along with IAV endocytosis. The have sufficiently responded to my critique adding in additional experiments where needed. They have also significantly reorganized the manuscript and added in the detailed information that was requested by the reviewers. This is a much improved manuscript.

We sincerely thank the reviewer for the careful re-evaluation of our revised manuscript and for the encouraging comments. We are pleased that the reviewer finds the revised manuscript to be substantially improved.

Referee #3:

The authors have addressed most of those comments made during our previous review of this manuscript. Two key outstanding data remain unclear and these form the focus of what the authors highlight as the major point of their study: "The primary focus of this work was to define the role of TRIM62 in regulating WASH-retromer association and the impact of this regulation on IAV entry".

The authors sort to understand how TRIM62 binding to FAM21 affects retromer association through GFP-nanotrap IPs of expressed VPS35-GFP. They state that under their conditions they were unable to detect VPS35 binding to endogenous FAM21, an interaction that numerous other labs have validated and defined by IP approaches, but they were able to detect endogenous WASH1 binding (i.e. FAM21 independent binding of WASH1 to VPS35). This raises some concern as to what they are examining in these assays and the inference of the effect of TRIM62 on the FAM21-VPS35 axis is therefore indirect.

The authors have also tried to provide additional data of the direct nature of the TRIM62 binding to FAM21. They show a very low affinity association between the recombinant PRY-SPRY domain of TRIM62 and the amino-terminal region of FAM21. As I still have reservations as to the validity of this interaction, I think two important points should be made clear in their manuscript. First, AlphaFold modelling fails to predict with any confidence the molecular details of this potential association. Secondly, the authors must make it clear that this region of FAM21 is normally buried within the global head region of the functional pentameric WASH complex. Without evidence that TRIM62 can associate with FAM21 when it is bound to strumpellin, CCDC53, WASH and SWIP, the conclusion that TRIM62 can directly bind to the entirely WASH complex remains to be established.

Collectively, the authors conclusion of the FAM21-VPS35 axis as a 'molecular checkpoint' is, in my opinion, remains an over-interpretation of their data. The title and abstract should be edited to better reflect these mechanistic limitations.

We sincerely thank the reviewer for the thoughtful and constructive comments, which have substantially improved the manuscript. We appreciate the concerns regarding the mechanistic interpretation of the TRIM62-FAM21 interaction and the regulation of WASH-retromer association.

In response, we have revised the Title, Abstract, and Discussion to better reflect the mechanistic scope of our study and to avoid overinterpretation. Specifically, we no longer describe the VPS35-FAM21 axis as a definitive "molecular checkpoint" and have moderated

our conclusions throughout the manuscript. We now present our findings as evidence that TRIM62 regulates WASH-retromer association and thereby influences WASH localization and IAV entry, while acknowledging that the precise molecular mechanism by which TRIM62 modulates this interaction remains to be fully established.

We fully agree that the interaction between VPS35 and FAM21 has been well established by multiple independent studies. However, in our GFP-nanotrap pulldown experiments, endogenous WASH1 was reproducibly detected in VPS35-GFP precipitates, whereas endogenous FAM21 was not. This observation likely reflects the biochemical properties of the WASH-retromer complex in the conditions that were used in our study rather than the absence of a VPS35-FAM21 interaction. Importantly, this observation is not without precedent. Chen *et al.* (PNAS, 2019) reported that endogenous VPS35 from mouse brain co-immunoprecipitated efficiently with VPS26 but not with either FAM21 or WASH1. Likewise, a recent comprehensive VPS35 interactome study by Williams et al. (https://www.biorxiv.org/content/10.64898/2026.04.09.717005v1) failed to recover WASH complex components by tandem affinity purification or endogenous brain co-immunoprecipitation, despite their established association with VPS35. The authors suggested that the interaction is likely labile and context dependent, requiring biochemical stabilization such as chemical crosslinking for efficient recovery. We have now clarified the limitations of our interaction studies.

As pointed out by the reviewer, we acknowledge that AlphaFold did not confidently predict the interaction interface between TRIM62 and FAM21. However, we would like to emphasize that FAM21 is a structurally complex protein with long, highly disordered region, and AlphaFold predictions involving FAM21 should be interpreted with caution. Indeed, the predicted interface between TRIM62 and FAM21 yielded an interface predicted TM (ipTM) score of ~0.2, indicating low confidence. Notably, the well-established interaction between FAM21 and WASH1 also yields only a moderate ipTM score (~0.4), despite extensive experimental validation in the literature. According to AlphaFold guidelines, ipTM values >0.8 generally indicate high-confidence interface predictions, whereas values <0.6 are considered unreliable. Thus, the inability of AlphaFold to confidently predict the TRIM62-FAM21 interface does not, in itself, argue against the existence of the interaction. Rather, it highlights the limitations of structure prediction for proteins containing substantial intrinsically disordered regions, such as FAM21. For this reason, we believe that the biochemical, Y2H, and MST analyses presented in our study provide more reliable evidence supporting the interaction than AlphaFold predictions alone. Despite the low confidence of the prediction, AlphaFold suggested a potential interaction between the C-terminal PRY-SPRY domain of TRIM62 and the N-terminal globular region of FAM21, consistent with our biochemical mapping studies as shown in the following figure.

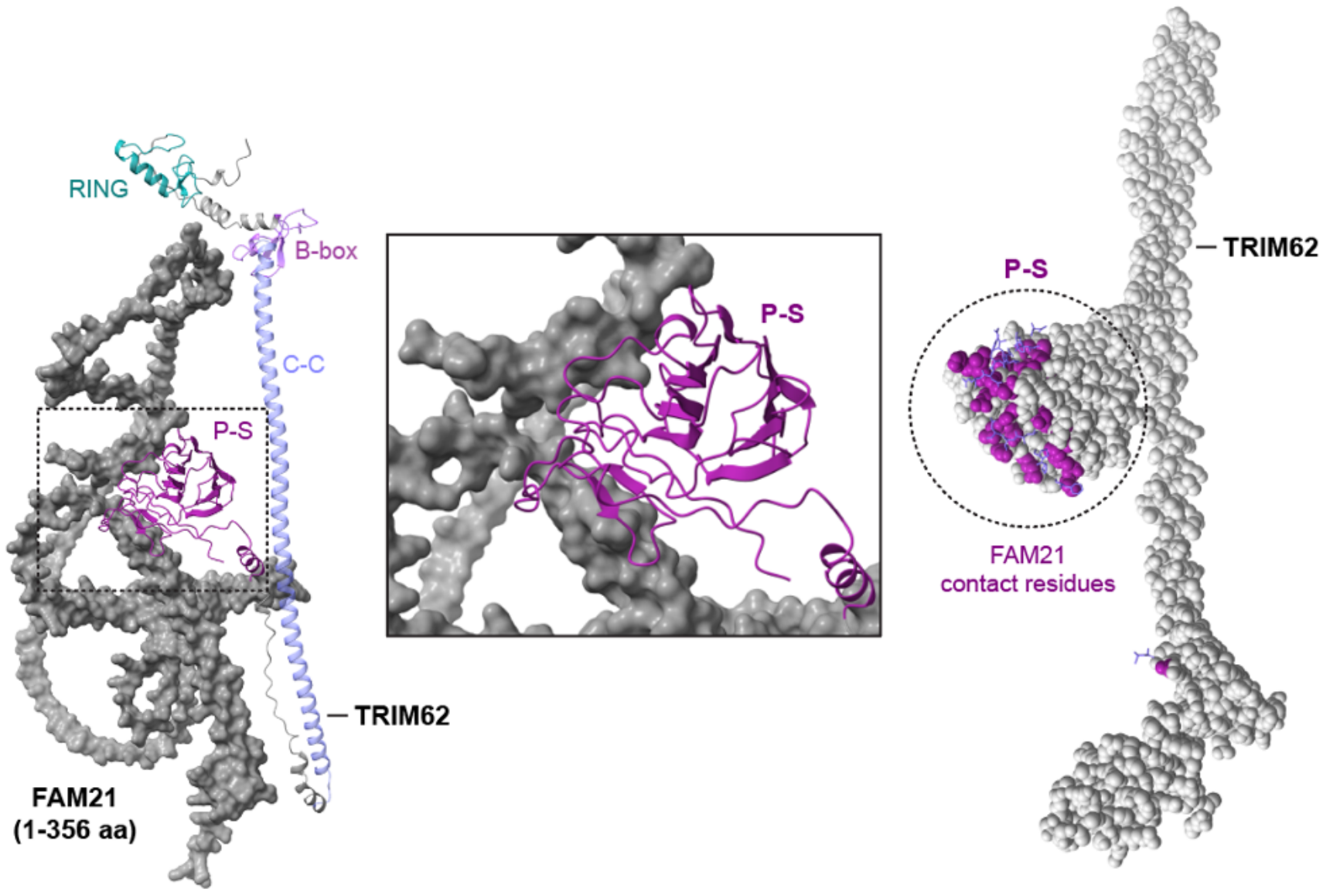

Our MST analysis showed that full-length TRIM62 binds the FAM21 globular domain with substantially higher affinity (nanomolar range) than the isolated PRY-SPRY domain (micromolar range). This difference suggests that regions outside the PRY-SPRY domain may contribute to the overall affinity of the interaction, either by stabilizing the interaction interface or by influencing the conformation of the PRY-SPRY domain. We did not investigate the structural basis for this difference, as it was beyond the scope of the present study. We have now acknowledged this limitation in the revised Discussion.

Despite these mechanistic limitations, we believe our collective genetic and functional evidence supports the conclusion that TRIM62 acts as an upstream regulator of WASH-dependent IAV entry. The enhanced WASH-retromer association observed upon TRIM62 depletion, the reciprocal effects of TRIM62 and VPS35 on WASH localization and viral entry, the genetic interaction analyses, and the requirement of the TRIM62 PRY-SPRY domain for both limiting WASH-retromer association and promoting viral entry together support a model in which TRIM62 antagonizes WASH-retromer coupling to promote efficient viral endocytosis. We have taken care to present this as a working mechanistic model that will require further structural and biochemical investigation.

Feedback from Referee #2 on the report from Referee #3:

It seems that many of the remaining issues can be addressed through changing the title, abstract, softening their conclusions and adding appropriate text to the Discussion. The FAM21 co-IP with VPS35 does require specific lysis buffers that favor the association. It's likely they did not use any of these. I agree that TRIM62 SPRY domain binding to the FAM21 globular head could be an issue. However, since the structure of this domain along with the other 4 components of the WASH complex is unknown, there could be a surface-exposed FAM21 element that mediates the interaction. Regardless, this needs to be discussed in the context of the manuscript as pointed out by reviewer 3.

We again thank the reviewer for these thoughtful and constructive suggestions. In response, we have substantially revised the Title, Abstract, and Discussion to better reflect the

mechanistic scope of our study and to avoid overstating our conclusions. In particular, we have softened the language throughout the manuscript to clearly distinguish our experimental findings from the mechanistic model they support. We discussed that we could not detect endogenous FAM21 in VPS35 pulldown assay under the conditions employed in our study and acknowledged that further optimization will be required for detection of this interaction. We also appreciate the reviewer's perspective regarding the interaction between the TRIM62 PRY-SPRY domain and the FAM21 globular region. We agree that, because the architecture of the intact pentameric WASH complex remains incompletely resolved, it remains possible that a surface-exposed region of FAM21 mediates this interaction. We have incorporated this point into the Discussion, and discussed how this interaction that occurs within the assembled WASH complex remains to be determined.

Indranil Banerjee
IISER Mohali
India

Dear Dr. Banerjee,

I am very pleased to accept your manuscript for publication in the next available issue of EMBO reports. Thank you for your contribution to our journal.

Your manuscript will be processed for publication by EMBO Press. It will be copy edited and you will receive page proofs prior to publication. Please note that you will be contacted by Springer Nature Author Services to complete licensing and payment information.

You may qualify for financial assistance for your publication charges - either via a Springer Nature fully open access agreement or an EMBO initiative. Check your eligibility: https://link.springer.com/journal/44319/how-to-publish-with-us

Should you be planning a Press Release on your article, please notify the production team via this form in order to coordinate publication and release dates: https://forms.cloud.microsoft/e/eH4A8JVP6g

If you have any questions, please do not hesitate to contact the Editorial Office. Thank you for your contribution to EMBO Reports.

Yours sincerely,

Martina Rembold, PhD
Senior Editor
EMBO reports

>>> Please note that it is EMBO Reports policy for the transcript of the editorial process (containing referee reports and your response letter) to be published as an online supplement to each paper. If you do NOT want this, you will need to inform the Editorial Office via email immediately. More information is available here: https://link.springer.com/partners/embo-press/editorial-policies#Peer%20review

EMBO Press Author Checklist

Corresponding Author Name: Dr. Indranil Banerjee	
Journal Submitted to: EMBO Reports	
Manuscript Number: EMBOR-64001V2	

USEFUL LINKS FOR COMPLETING THIS FORM
The EMBO Journal - Author Guidelines
EMBO Reports - Author Guidelines
Molecular Systems Biology - Author Guidelines
EMBO Molecular Medicine - Author Guidelines

Reporting Checklist for Life Science Articles (updated January
This checklist is adapted from Materials Design Analysis Reporting (MDAR) Checklist for Authors. MDAR establishes a minimum set of requirements in transparent reporting in the life sciences (see Statement of Task: 10.31222/osf.io/9sm4x). Please follow the journal's guidelines in preparing your
Please note that a copy of this checklist will be published alongside your article.

Abridged guidelines for figures
1. Data
The data shown in figures should satisfy the following conditions:
- ➞ the data were obtained and processed according to the field's best practice and are presented to reflect the results of the experiments in an accurate and unbiased manner.
- ➞ ideally, figure panels should include only measurements that are directly comparable to each other and obtained with the same assay.
- ➞ plots include clearly labeled error bars for independent experiments and sample sizes. Unless justified, error bars should not be shown for technical
- ➞ if n<5, the individual data points from each experiment should be plotted. Any statistical test employed should be justified.
- ➞ Source Data should be included to report the data underlying figures according to the guidelines set out in the authorship guidelines on Data

2. Captions
Each figure caption should contain the following information, for each panel where they are relevant:
- ➞ a specification of the experimental system investigated (eg cell line, species name).
- ➞ the assay(s) and method(s) used to carry out the reported observations and measurements.
- ➞ an explicit mention of the biological and chemical entity(ies) that are being measured.
- ➞ an explicit mention of the biological and chemical entity(ies) that are altered/varied/perturbed in a controlled manner.
- ➞ the exact sample size (n) for each experimental group/condition, given as a number, not a range;
- ➞ a description of the sample collection allowing the reader to understand whether the samples represent technical or biological replicates (including how many animals, litters, cultures, etc.).
- ➞ a statement of how many times the experiment shown was independently replicated in the laboratory.
- ➞ definitions of statistical methods and measures:
  - common tests, such as t-test (please specify whether paired vs. unpaired), simple χ2 tests, Wilcoxon and Mann-Whitney tests, can be unambiguously identified by name only, but more complex techniques should be described in the methods section;
  - are tests one-sided or two-sided?
  - are there adjustments for multiple comparisons?
  - exact statistical test results, e.g., P values = x but not P values < x;
  - definition of 'center values' as median or average;
  - definition of error bars as s.d. or s.e.m.

Please complete ALL of the questions below.
Select "Not Applicable" only when the requested information is not relevant for your study.

Materials

Newly Created Materials	Information included in the manuscript?	In which section is the information available? (Reagents and Tools Table, Materials and Methods, Figures, Data Availability Section)
New materials and reagents need to be available; do any restrictions apply?	Yes	Reagents and Tools Table, Materials and Methods. No restrictions apply

Antibodies	Information included in the manuscript?	In which section is the information available? (Reagents and Tools Table, Materials and Methods, Figures, Data Availability Section)
For **antibodies** provide the following information:<br>- Commercial antibodies: RRID (if possible) or supplier name, catalogue number and or/clone number<br>- Non-commercial: RRID or citation	Yes	Reagents and Tools Table, Materials and Methods

DNA and RNA sequences	Information included in the manuscript?	In which section is the information available? (Reagents and Tools Table, Materials and Methods, Figures, Data Availability Section)
**Short novel DNA or RNA including primers, probes**: provide the sequences.	Yes	Reagents and Tools Table

Cell materials	Information included in the manuscript?	In which section is the information available? (Reagents and Tools Table, Materials and Methods, Figures, Data Availability Section)
**Cell lines:** Provide species information, strain. Provide accession number in repository **OR** supplier name, catalog number, clone number, and/**OR** RRID.	Yes	Reagents and Tools Table, Materials and Methods, Figures
**Primary cultures:** Provide species, strain, sex of origin, genetic modification status.	Not Applicable	
Report if the cell lines were recently **authenticated** (e.g., by STR profiling) and tested for mycoplasma contamination.	Not Applicable	

Experimental animals	Information included in the manuscript?	In which section is the information available? (Reagents and Tools Table, Materials and Methods, Figures, Data Availability Section)
**Laboratory animals or Model organisms:** Provide species, strain, sex, age, genetic modification status. Provide accession number in repository **OR** supplier name, catalog number, clone number, **OR** RRID.	Not Applicable	
**Animal observed in or captured from the field:** Provide species, sex, and age where possible.	Not Applicable	
Please detail **housing and husbandry conditions**.	Not Applicable	

Plants and microbes	Information included in the manuscript?	In which section is the information available? (Reagents and Tools Table, Materials and Methods, Figures, Data Availability Section)
**Plants:** provide species and strain, ecotype and cultivar where relevant, unique accession number if available, and source (including location for collected wild specimens).	Not Applicable	
**Microbes:** provide species and strain, unique accession number if available, and source.	Yes	Reagents and Tools Table, Materials and Methods, Figures

Human research participants	Information included in the manuscript?	In which section is the information available? (Reagents and Tools Table, Materials and Methods, Figures, Data Availability Section)
If collected and within the bounds of privacy constraints report on age, sex and gender or ethnicity for all study participants.	Not Applicable	

Core facilities	Information included in the manuscript?	In which section is the information available? (Reagents and Tools Table, Materials and Methods, Figures, Data Availability Section)
If your work benefited from core facilities, was their service mentioned in the acknowledgments section?	Yes	Acknowledgements section

Design

Study protocol	Information included in the manuscript?	In which section is the information available? (Reagents and Tools Table, Materials and Methods, Figures, Data Availability Section)
If study protocol has been **pre-registered, provide DOI in the manuscript**. For clinical trials, provide the trial registration number **OR** cite DOI.	Not Applicable	
Report the **clinical trial registration number** (at ClinicalTrials.gov or equivalent), where applicable.	Not Applicable	

Laboratory protocol	Information included in the manuscript?	In which section is the information available? (Reagents and Tools Table, Materials and Methods, Figures, Data Availability Section)
Provide DOI OR other citation details if **external detailed step-by-step protocols** are available.	Not Applicable	

Experimental study design and statistics	Information included in the manuscript?	In which section is the information available? (Reagents and Tools Table, Materials and Methods, Figures, Data Availability Section)
Include a statement about **sample size** estimate even if no statistical methods were used.	Yes	Figures
Were any steps taken to minimize the effects of subjective bias when allocating animals/samples to treatment (e.g. **randomization procedure**)? If yes, have they been described?	Not Applicable	
Include a statement about **blinding** even if no blinding was done.	Not Applicable	
Describe **inclusion/exclusion criteria** if samples or animals were excluded from the analysis. Were the criteria pre-established?<br><br>If sample or data points were omitted from analysis, report if this was due to attrition or intentional exclusion and provide justification.	Not Applicable	
For every figure, are **statistical tests** justified as appropriate? Do the data meet the assumptions of the tests (e.g., normal distribution)? Describe any methods used to assess it. Is there an estimate of variation within each group of data? Is the variance similar between the groups that are being statistically compared?	Yes	Materials and Methods, Figures

Sample definition and in-laboratory replication	Information included in the manuscript?	In which section is the information available? (Reagents and Tools Table, Materials and Methods, Figures, Data Availability Section)
In the figure legends: state number of times the experiment was **replicated** in laboratory.	Yes	Materials and Methods, Figures
In the figure legends: define whether data describe **technical or biological replicates**.	Yes	Figures

Ethics

Ethics	Information included in the manuscript?	In which section is the information available? (Reagents and Tools Table, Materials and Methods, Figures, Data Availability Section)
Studies involving **human participants**: State details of **authority granting ethics approval** (IRB or equivalent committee(s), provide reference number for approval.	Not Applicable	
Studies involving **human participants**: Include a statement confirming that **informed consent** was obtained from all subjects and that the experiments conformed to the principles set out in the WMA Declaration of Helsinki and the Department of Health and Human Services Belmont Report.	Not Applicable	
Studies involving **human participants:** For publication of **patient photos**, include a statement confirming that consent to publish was obtained.	Not Applicable	
Studies involving experimental **animals**: State details of **authority granting ethics approval** (IRB or equivalent committee(s), provide reference number for approval. Include a statement of compliance with ethical regulations.	Not Applicable	
Studies involving **specimen and field samples:** State if relevant **permits** obtained, provide details of authority approving study; if none were required, explain why.	Not Applicable	

Dual Use Research of Concern (DURC)	Information included in the manuscript?	In which section is the information available? (Reagents and Tools Table, Materials and Methods, Figures, Data Availability Section)
Could your study fall under dual use research restrictions? Please check biosecurity documents and list of **select agents and toxins** (CDC): https://www.selectagents.gov/sat/list.htm	Not Applicable	
If you used a select agent, is the security level of the lab appropriate and reported in the manuscript?	Not Applicable	
If a study is subject to dual use research of concern regulations, is the name of the **authority granting approval and reference number** for the regulatory approval provided in the manuscript?	Not Applicable	

Reporting

The MDAR framework recommends adoption of discipline-specific guidelines, established and endorsed through community initiatives. Journals have their own policy about requiring specific guidelines and recommendations to complement MDAR.

Adherence to community standards	Information included in the manuscript?	In which section is the information available? (Reagents and Tools Table, Materials and Methods, Figures, Data Availability Section)
State if relevant guidelines or checklists (e.g., **ICMJE, MIBBI, ARRIVE, PRISMA**) have been followed or provided.	Not Applicable	
For **tumor marker prognostic studies,** we recommend that you follow the **REMARK** reporting guidelines (see link list at top right). See author guidelines, under 'Reporting Guidelines'. Please confirm you have followed these guidelines.	Not Applicable	
For **phase II and III randomized controlled trials**, please refer to the **CONSORT** flow diagram (see link list at top right) and submit the CONSORT checklist (see link list at top right) with your submission. See author guidelines, under 'Reporting Guidelines'. Please confirm you have submitted this list.	Not Applicable	

Data Availability

Data availability	Information included in the manuscript?	In which section is the information available? (Reagents and Tools Table, Materials and Methods, Figures, Data Availability Section)
Have **primary datasets** been deposited according to the journal's guidelines (see 'Data Deposition' section) and the respective accession numbers provided in the Data Availability Section?	Not Applicable	
Were **human clinical and genomic datasets** deposited in a public access-controlled repository in accordance to ethical obligations to the patients and to the applicable consent agreement?	Not Applicable	
Are **computational models** that are central and integral to a study available without restrictions in a machine-readable form? Were the relevant accession numbers or links provided?	Not Applicable	
If publicly available data were reused, provide the respective **data citations in the reference list**.	Not Applicable	
