# Supplementary material for: TRIM62 facilitates influenza A virus entry by regulating WASH-dependent endosomal trafficking
Source: EMBO Rep. 2026 Sep 19;27(20):5953–90. doi: 10.1038/s44319-026-00942-x (PMC13694701; doi:10.1038/s44319-026-00942-x)
Supplement: Supplementary file 3 [file 44319_2026_942_MOESM9_ESM.pdf]

Expanded View Figures

Figure EV1. TRIM62 depletion attenuates IAV infection across different viral strains and cell lines.

(**A**) Quantification of mRNA levels of *TRIM62* upon KD in A549 cells using two independent siRNAs targeting TRIM62 ($N = 3$ independent experiments; mean ± SD; error bars represent SD; one-way ANOVA with Dunnett's multiple-comparisons test). *P* values for siControl vs siTRIM62 #2: $P < 0.0001$; siControl vs siTRIM62 #3: $P = 0.0003$. (**B**) Chromatogram (top) showing DNA sequence downstream of the gRNA-targeted genomic region from WT and TRIM62 KO A549 cells. gRNA targeting region in exon 2 is highlighted. DNA sequences from WT and TRIM62 KO are translated using Expasy Translate tool and pairwise aligned using EMBOSS Needle tool (bottom), indicating premature protein termination in the KO cells. (**C**) Quantification of mRNA levels of *TRIM62* in TRIM62 KO cells compared to WT A549 cells ($N = 3$ independent experiments; mean ± SD; error bars represent SD; unpaired two-tailed *t*-test; $P = 2.91 \times 10^{-7}$). (**D–G**) High-content confocal images (**D**) and quantification of IAV infection in control and TRIM62 KD A549 cells infected with WSN (**E**), NYMC (**F**), and Udorn (**G**) for 10 h. NP (cyan), and nuclei (magenta). Scale bars, 20 µm. $n \geq 12{,}000$ cells/assay ($N = 3$ independent experiments; mean ± SD; error bars represent SD; one-way ANOVA with Dunnett's multiple-comparisons test). *P* values for siControl vs siTRIM62: $P = 0.0002$ (**E**); $P = 0.002$ (**F**); $P = 0.0003$ (**G**). (**H–K**) High-content confocal images (**H**) and quantification of IAV infection in HeLa (**I**), HepG2 (**J**), and Huh-7 (**K**) cells infected with IAV (X-31) for 10 h. Scale bars, 20 µm. $n \geq 4500$ cells/assay ($N = 3$ independent experiments; mean ± SD; error bars represent SD; one-way ANOVA with Dunnett's multiple-comparisons test). *P* values for siControl vs siTRIM62: $P < 0.0001$ (**I**, **J**); $P = 0.0025$ (**K**). (**L–O**) High-content confocal images (**L**) and quantification of IAV infection in TRIM62 KO and WT A549 cells infected with WSN (**M**), NYMC (**N**), and Udorn (**O**) for 10 h. Scale bars, 20 µm. $n \geq 10{,}000$ cells/assay ($N = 3$ independent experiments; mean ± SD; error bars represent SD; one-way ANOVA with Dunnett's multiple-comparisons test). *P* values for WT vs TRIM62 KO: $P = 0.01$ (**M**); $P = 0.007$ (**N**); $P = 0.01$ (**O**).

© The Author(s)

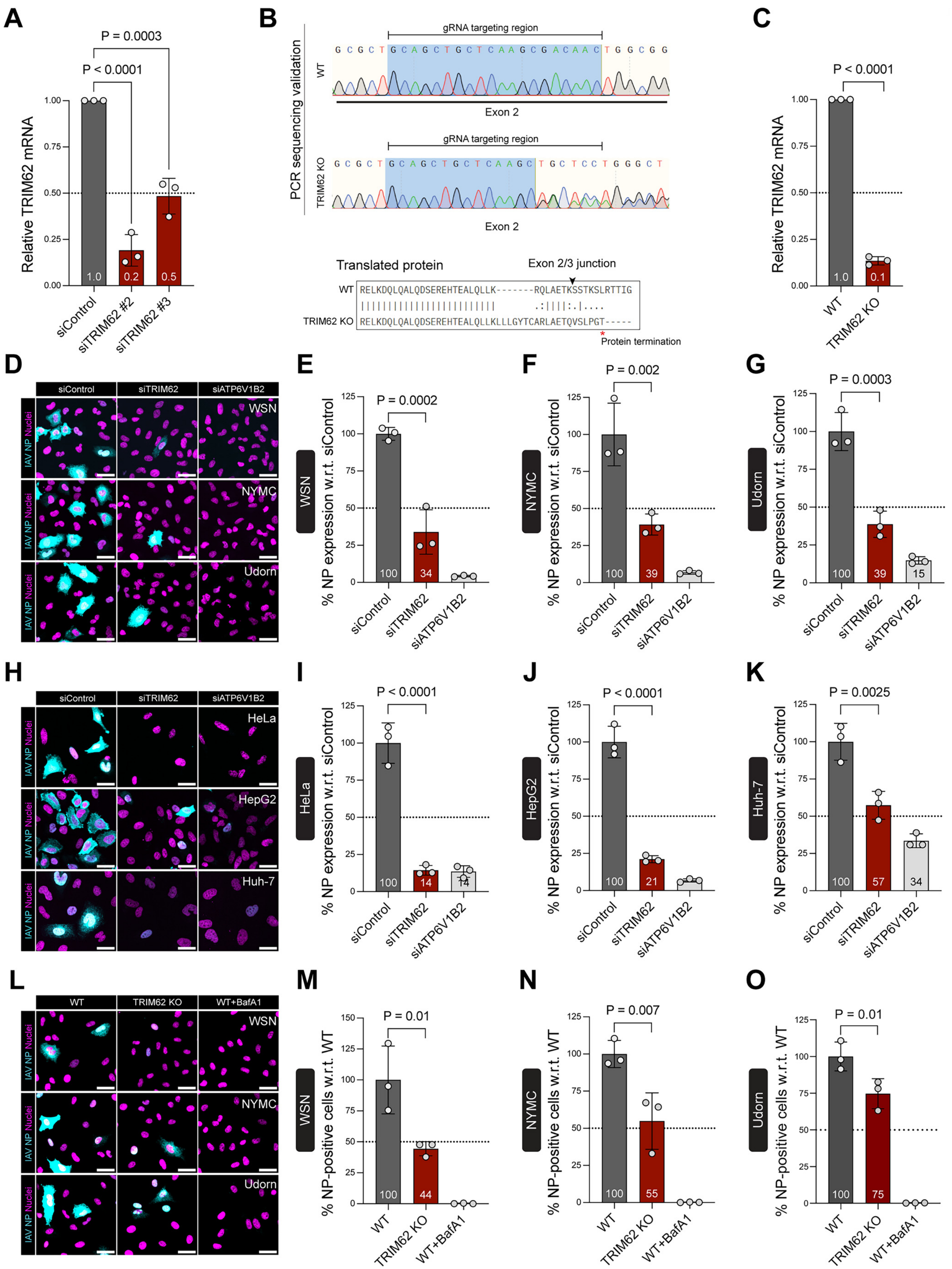
A
P = 0.0003
P < 0.0001
Relative TRIM62 mRNA
1.0
0.2
0.5
siControl
siTRIM62 #2
siTRIM62 #3
B
PCR sequencing validation
WT
TRIM62 KO
gRNA targeting region
Exon 2
Translated protein
Exon 2/3 junction
WT RELKDQLQALQDSEREHTEALQLLK-------RQLAETKSSTKSLRTTIG
TRIM62 KO RELKDQLQALQDSEREHTEALQLLKLLLGYTCARLAETQVSLPGT-----
Protein termination
C
P < 0.0001
Relative TRIM62 mRNA
1.0
0.1
WT
TRIM62 KO
D
siControl
siTRIM62
siATP6V1B2
IAV NP Nuclei
WSN
NYMC
Udorn
E
WSN
P = 0.0002
% NP expression w.r.t. siControl
100
34
siControl
siTRIM62
siATP6V1B2
F
NYMC
P = 0.002
% NP expression w.r.t. siControl
100
39
siControl
siTRIM62
siATP6V1B2
G
Udorn
P = 0.0003
% NP expression w.r.t. siControl
100
39
15
siControl
siTRIM62
siATP6V1B2
H
siControl
siTRIM62
siATP6V1B2
IAV NP Nuclei
HeLa
HepG2
Huh-7
I
HeLa
P < 0.0001
% NP expression w.r.t. siControl
100
14
14
siControl
siTRIM62
siATP6V1B2
J
HepG2
P < 0.0001
% NP expression w.r.t. siControl
100
21
siControl
siTRIM62
siATP6V1B2
K
Huh-7
P = 0.0025
% NP expression w.r.t. siControl
100
57
34
siControl
siTRIM62
siATP6V1B2
L
WT
TRIM62 KO
WT+BafA1
IAV NP Nuclei
WSN
NYMC
Udorn
M
WSN
P = 0.01
% NP-positive cells w.r.t. WT
100
44
WT
TRIM62 KO
WT+BafA1
N
NYMC
P = 0.007
% NP-positive cells w.r.t. WT
100
55
WT
TRIM62 KO
WT+BafA1
O
Udorn
P = 0.01
% NP-positive cells w.r.t. WT
100
75
WT
TRIM62 KO
WT+BafA1

© The Author(s)

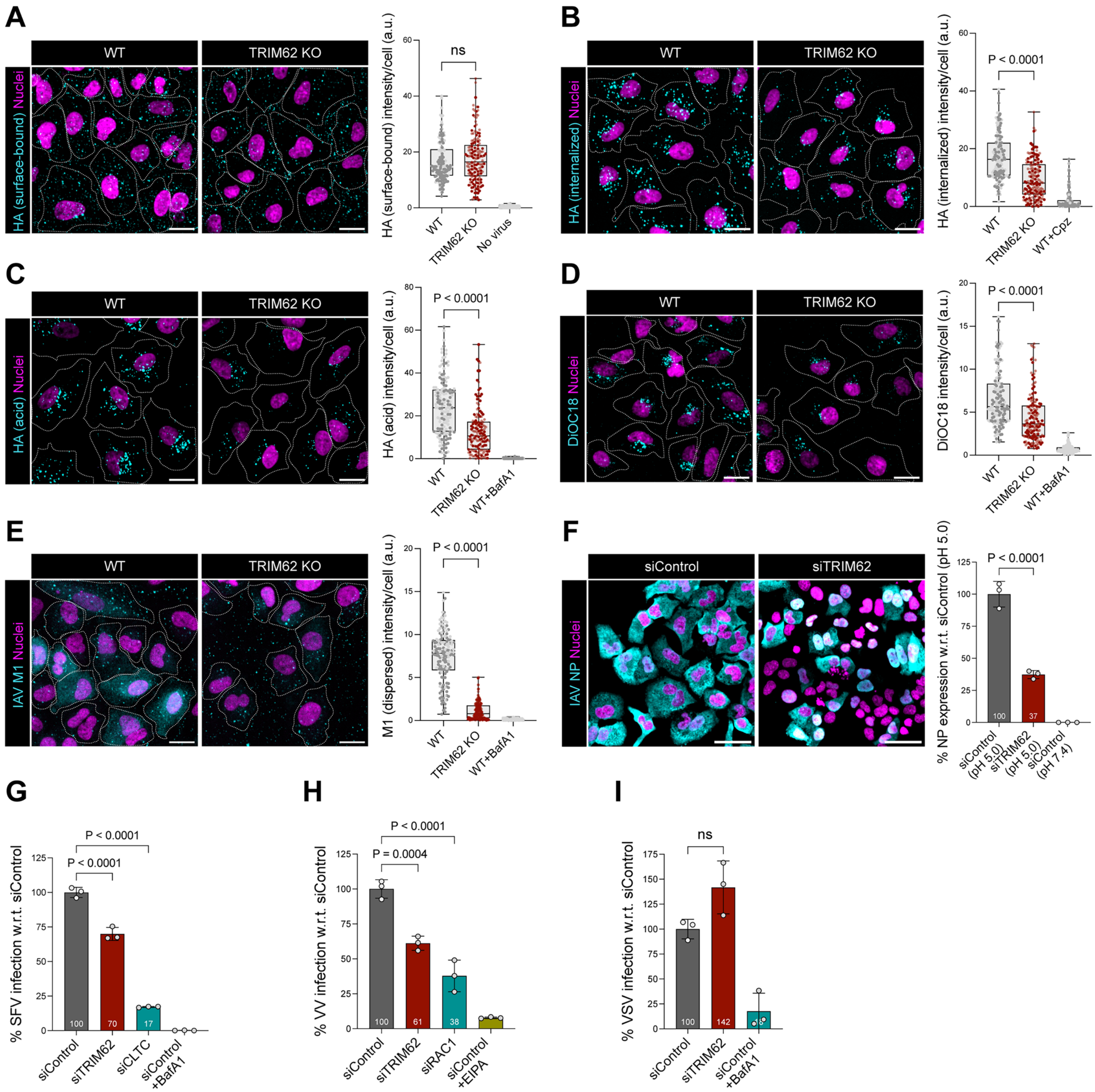
A
WT
TRIM62 KO
HA (surface-bound) Nuclei
HA (surface-bound) intensity/cell (a.u.)
ns
WT
TRIM62 KO
No virus
B
WT
TRIM62 KO
HA (internalized) Nuclei
HA (internalized) intensity/cell (a.u.)
P < 0.0001
WT
TRIM62 KO
WT+Cpz
C
WT
TRIM62 KO
HA (acid) Nuclei
HA (acid) intensity/cell (a.u.)
P < 0.0001
WT
TRIM62 KO
WT+BafA1
D
WT
TRIM62 KO
DiOC18 Nuclei
DiOC18 intensity/cell (a.u.)
P < 0.0001
WT
TRIM62 KO
WT+BafA1
E
WT
TRIM62 KO
IAV M1 Nuclei
M1 (dispersed) intensity/cell (a.u.)
P < 0.0001
WT
TRIM62 KO
WT+BafA1
F
siControl
siTRIM62
IAV NP Nuclei
% NP expression w.r.t. siControl (pH 5.0)
P < 0.0001
100
37
siControl (pH 5.0)
siTRIM62 (pH 5.0)
siControl (pH 7.4)
G
% SFV infection w.r.t. siControl
P < 0.0001
P < 0.0001
100
70
17
siControl
siTRIM62
siCLTC
siControl +BafA1
H
% VV infection w.r.t. siControl
P < 0.0001
P = 0.0004
100
61
38
siControl
siTRIM62
siRAC1
siControl +EIPA
I
% VSV infection w.r.t. siControl
ns
100
142
siControl
siTRIM62
siControl +BafA1

Figure EV2. TRIM62 deficiency blocks IAV endocytosis and inhibits multiple viruses.

(**A**) High-content confocal images (left) and intensity analysis (right) of surface-bound IAV particles detected by IIF against HA. HA (cyan) and nuclei (magenta). Cell boundaries are outlined. Scale bars, 20 µm. $n = 158$ cells/sample. (**B**) High-content confocal images (left) and intensity analysis (right) of internalized IAV particles (HA) at 30 min post-infection. HA (cyan) and nuclei (magenta). Scale bars, 20 µm. $n = 156$ cells/sample. (**C**) High-content confocal images (left) and intensity analysis (right) of acidified HA at 1 h.p.i. Scale bars, 20 µm. $n = 146$ cells/sample. (**D**) High-content confocal images (left) and intensity analysis (right) of DiOC18/R18-labeled IAV upon fusion at 1.5 h.p.i. DiOC18 (cyan) and nuclei (magenta). Scale bars, 20 µm. $n = 152$ cells/sample. (**E**) High-content confocal images (left) and intensity analysis (right) of cytosolic M1 (dispersed) upon M1 uncoating at 2 h.p.i. M1 (cyan) and nuclei (magenta). Scale bars, 20 µm. $n = 150$ cells/sample. (**F**) High-content confocal images (left) and quantification (right) of plasma membrane-bypass infection (NP expression) at pH 5 and pH 7.4 (negative control) in control and TRIM62 KD A549 cells. $n \geq 4500$ cells/assay ($N = 3$ independent experiments; mean ± SD; error bars represent SD; one-way ANOVA with Dunnett's multiple-comparisons test). P values for siControl vs siTRIM62: $P < 0.0001$ (pH 5.0). (**G**) Quantification of SFV infection in control and TRIM62 KD A549 cells at 24 h.p.i. $n \geq 7000$ cells/siRNA ($N = 3$ independent experiments; mean ± SD; error bars represent SD; one-way ANOVA with Dunnett's multiple-comparisons test). P values for siControl vs siTRIM62: $P < 0.0001$; siControl vs siCLTC: $P < 0.0001$. (**H**) Quantification of VV infection in control and TRIM62 KD A549 cells at 24 h.p.i. $n \geq 7000$ cells/siRNA ($N = 3$ independent experiments; mean ± SD; error bars represent SD; one-way ANOVA with Dunnett's multiple-comparisons test). P values for siControl vs siTRIM62: $P = 0.0004$; siControl vs siRAC1: $P < 0.0001$. (**I**) Quantification of VSV infection in control and TRIM62 KD A549 cells at 10 h.p.i. $n \geq 5000$ cells/siRNA ($N = 3$ independent experiments; mean ± SD; error bars represent SD; one-way ANOVA with Dunnett's multiple-comparisons test). P values for siControl vs siTRIM62: $P = 0.0664$. Data in (**A–E**) are represented as box and whiskers plots. The center line represents the median, the box boundaries correspond to the 25th and 75th percentiles (interquartile range), the whiskers extend to the minimum and maximum values, and all individual data points are overlaid ($N = 3$ independent experiments; one-way ANOVA with Dunnett's multiple-comparisons test). P values for WT vs TRIM62 KO: $P = 0.1420$ (**A**); $P < 0.0001$ (**B–E**). $P < 0.05$ (*), $P < 0.01$ (**), $P < 0.001$ (***), $P < 0.0001$ (****). P values < 0.05 were considered significant.

© The Author(s)

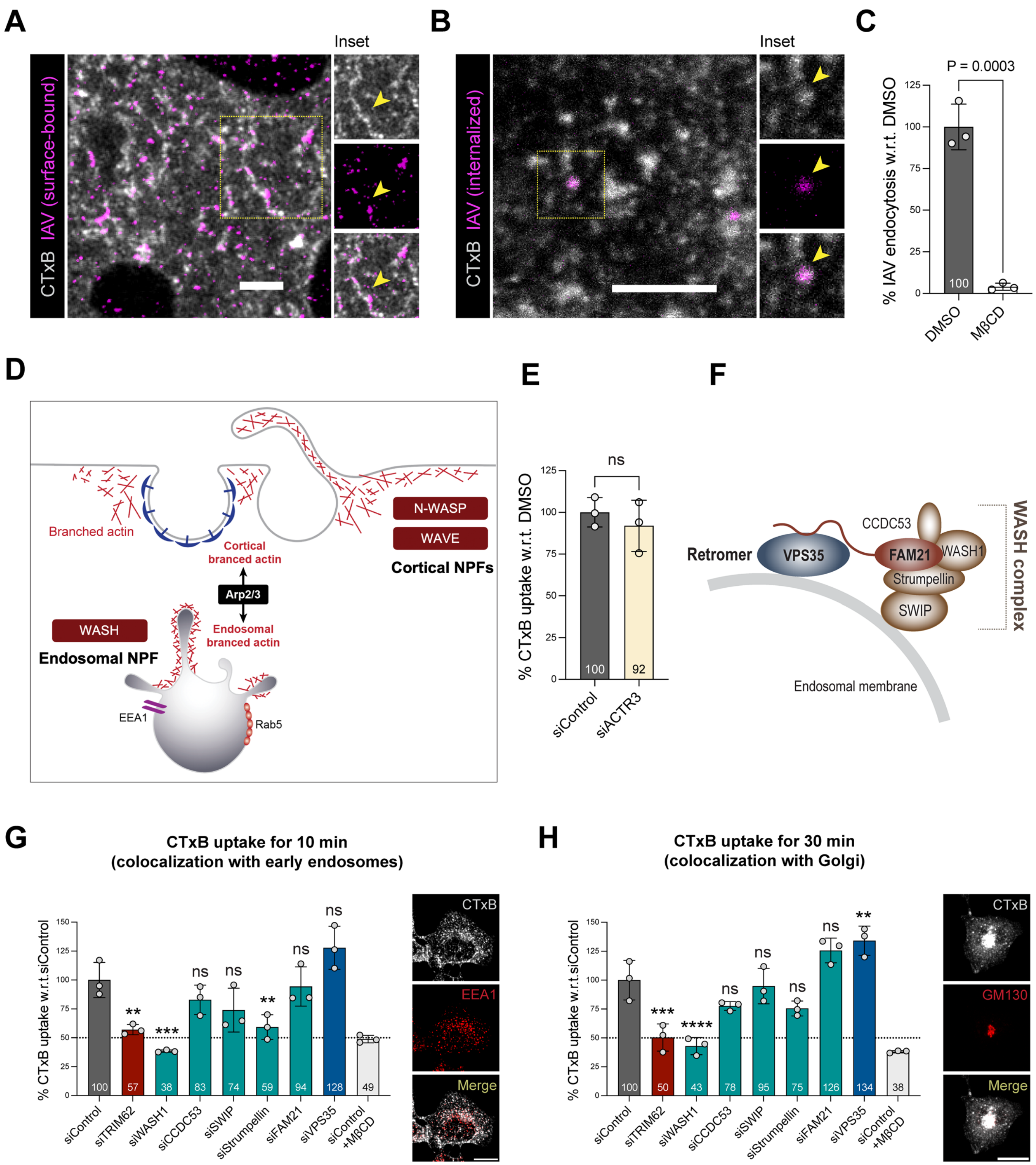
A
Inset
CTxB IAV (surface-bound)
B
Inset
CTxB IAV (internalized)
C
P = 0.0003
% IAV endocytosis w.r.t. DMSO
100
DMSO
MβCD
D
Branched actin
Cortical branched actin
N-WASP
WAVE
Cortical NPFs
Arp2/3
WASH
Endosomal NPF
Endosomal branched actin
EEA1
Rab5
E
ns
% CTxB uptake w.r.t. DMSO
100
92
siControl
siACTR3
F
Retromer
VPS35
CCDC53
FAM21
WASH1
Strumpellin
SWIP
WASH complex
Endosomal membrane
G
CTxB uptake for 10 min (colocalization with early endosomes)
% CTxB uptake w.r.t.siControl
siControl
siTRIM62
siWASH1
siCCDC53
siSWIP
siStrumpellin
siFAM21
siVPS35
siControl +MβCD
100 57 38 83 74 59 94 128 49
CTxB
EEA1
Merge
H
CTxB uptake for 30 min (colocalization with Golgi)
% CTxB uptake w.r.t.siControl
100 50 43 78 95 75 126 134 38
CTxB
GM130
Merge

Figure EV3. Association of IAV with CTxB-positive domains and concerted regulation of CTxB uptake by TRIM62 and WASH1.

(**A**) Confocal microscopy images showing surface-bound CTxB (gray) and IAV (magenta). Insets show the distribution of CTxB and IAV on lipid rafts and yellow arrowheads indicate colocalization. Scale bars, 5 µm. Representative images from three independent experiments are shown. (**B**) Confocal microscopy images showing CTxB (gray) and IAV (magenta), 10 min post-internalization. Insets show the distribution of internalized CTxB and IAV and yellow arrowheads indicate colocalization. Scale bars, 2 µm. Representative images from three independent experiments are shown. (**C**) Quantification of IAV endocytosis in DMSO-treated and MβCD-treated A549 cells. $n \geq 500$ cells/sample ($N = 3$ independent experiments; mean ± SD; error bars represent SD; unpaired two-tailed *t*-test; $P = 0.0003$). (**D**) Schematic representation of branched F-actin nucleation by Arp2/3 complex and cortical or endosomal NPFs. (**E**) Quantification of CTxB uptake in ACTR3 KD A549 cells at 30 min. $n \geq 2000$ cells/siRNA ($N = 3$ independent experiments; mean ± SD; error bars represent SD; unpaired two-tailed *t*-test; $P = 0.4737$). (**F**) Schematic showing the recruitment of pentameric WASH complex via retromer at endosomal surface, specifically mediated by the interaction of FAM21 with VPS35. (**G**, **H**) Quantification of CTxB uptake in control and KD A549 cells post 10 min (**G**) and 30 min (**H**) internalization. $n \geq 1000$ cells/sample. Images show CTxB uptake through colocalization of CTxB with EEA1-positive endosomes at 10 min (**G**) and with GM130-positive Golgi compartments at 30 min (**H**), respectively. Scale bars, 20 µm. Data in (**G**) were analyzed using one-way ANOVA with Dunnett's multiple-comparisons test. $N = 3$ independent experiments; mean ± SD; error bars represent SD. *P* values for siControl vs siTRIM62: $P = 0.0051$; siControl vs siWASH1: $P = 0.0001$; siControl vs siCCDC53: $P = 0.4889$; siControl vs siSWIP: $P = 0.1273$; siControl vs siStrumpellin: $P = 0.0079$; siControl vs siFAM21: $P = 0.9948$; siControl vs siVPS35: $P = 0.0924$. Data in (**H**) were analyzed using one-way ANOVA with Dunnett's multiple-comparisons test. $N = 3$ independent experiments; mean ± SD; error bars represent SD. *P* values for siControl vs siTRIM62: $P = 0.0002$; siControl vs siWASH1: $P < 0.0001$; siControl vs siCCDC53: $P = 0.1089$; siControl vs siSWIP: $P = 0.9920$; siControl vs siStrumpellin: $P = 0.0684$; siControl vs siFAM21: $P = 0.0537$; siControl vs siVPS35: $P = 0.0073$. $P < 0.05$ (*), $P < 0.01$ (**), $P < 0.001$ (***), $P < 0.0001$ (****). *P* values < 0.05 were considered significant.

 © The Author(s)

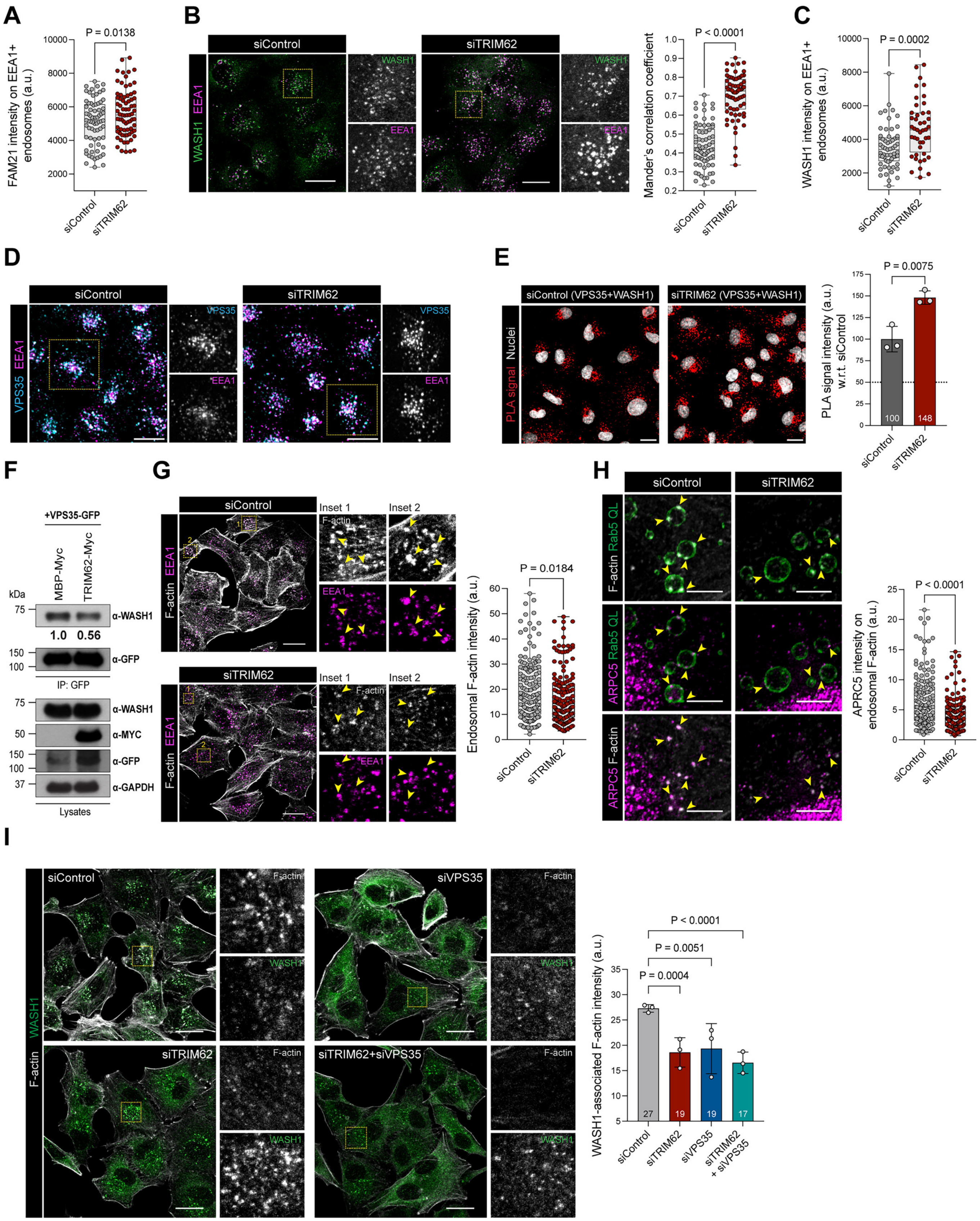
A
B
C
D
E
F
G
H
I
siControl
siTRIM62
siVPS35
siTRIM62+siVPS35
FAM21 intensity on EEA1+ endosomes (a.u.)
P = 0.0138
Mander's correlation coefficient
P < 0.0001
WASH1 intensity on EEA1+ endosomes (a.u.)
P = 0.0002
WASH1 EEA1
VPS35 EEA1
siControl (VPS35+WASH1)
siTRIM62 (VPS35+WASH1)
PLA signal Nuclei
PLA signal intensity (a.u.) w.r.t. siControl
P = 0.0075
100
148
+VPS35-GFP
MBP-Myc
TRIM62-Myc
kDa
α-WASH1
1.0 0.56
α-GFP
IP: GFP
α-MYC
α-GAPDH
Lysates
Inset 1
Inset 2
F-actin EEA1
Endosomal F-actin intensity (a.u.)
P = 0.0184
F-actin Rab5 QL
ARPC5 Rab5 QL
ARPC5 F-actin
APRC5 intensity on endosomal F-actin (a.u.)
P < 0.0001
F-actin WASH1
WASH1-associated F-actin intensity (a.u.)
P = 0.0004
P = 0.0051
27
19
19
17
siTRIM62 + siVPS35

Figure EV4. TRIM62 deficiency enhances endosomal recruitment of WASH but impairs its NPF activity.

(**A**) Quantification of FAM21 intensity on EEA1-positive endosomes. $n \geq 45$ cells/siRNA ($N = 3$ independent experiments; mean ± SD; error bars represent SD; unpaired two-tailed *t*-test; $P = 0.0138$). Representative images are shown in Fig. 5A. (**B**) Confocal microscopy images (left) showing WASH1 (green) and EEA1-positive endosomes (magenta) in control and TRIM62 KD A549 cells. Insets show the distribution of WASH1 and EEA1. Scale bars, 20 µm. Quantification (right) of WASH1-EEA1 colocalization based on Mander's correlation coefficient. $n = 70$ cells/siRNA ($N = 3$ independent experiments; mean ± SD; error bars represent SD; unpaired two-tailed *t*-test; $P = 2.46 \times 10^{-27}$). (**C**) Quantification of WASH1 intensity on EEA1-positive endosomes. $n \geq 45$ cells/siRNA ($N = 3$ independent experiments; mean ± SD; error bars represent SD; unpaired two-tailed *t*-test; $P = 0.0002$). (**D**) High-content confocal images showing VPS35 (cyan) and EEA1-positive endosomes (magenta) in control and TRIM62 KD A549 cells. Insets show the distribution of VPS35 and EEA1. Scale bars, 20 µm. For quantification, see Fig. 5B,C. (**E**) High-content confocal images (left) and quantification (right) of PLA signals from VPS35-WASH1 interaction. Scale bars, 20 µm. $n \geq 750$ cells/sample ($N = 3$ independent experiments; mean ± SD; error bars represent SD; unpaired two-tailed *t*-test; $P = 0.0075$). (**F**) Pulldown assay showing interactions between VPS35-GFP and WASH1. HEK293T cells were co-transfected with VPS35-GFP and MBP-Myc or TRIM62-Myc, and lysates were incubated with GFP-Trap beads. Western blotting was performed to detect endogenous WASH1 in the precipitates. The western blots are representative of three independent experiments. (**G**) Confocal microscopy images (left) showing F-actin (gray) and EEA1-positive endosomes (magenta) in control and TRIM62 KD cells. Yellow arrowheads within the insets show the distribution of F-actin at endosomes. Scale bar, 20 µm. Scatter plot (right) showing F-actin intensity on EEA1-positive endosomes. $n = 150$ F-actin patches/siRNA ($N = 3$ independent experiments; mean ± SD; error bars represent SD; unpaired two-tailed *t*-test; $P = 0.0184$). (**H**) Confocal microscopy images (left) showing ARPC5 on GFP-Rab5QL-positive endosomes in control and TRIM62-depleted cells. Yellow arrowheads indicate F-actin and/or ARPC5 localization on enlarged, Rab5-QL-positive endosomes. Scale bars, 5 µm. Scatter plot (right) showing ARPC5 intensity within F-actin-positive regions on GFP-Rab5QL-positive endosomes. $n = 150$ F-actin patches/siRNA ($N = 3$ independent experiments; mean ± SD; error bars represent SD; unpaired two-tailed *t*-test; $P = 1.68 \times 10^{-8}$). (**I**) Confocal microscopy images (left) showing WASH1 (green) and F-actin (gray) in control, TRIM62, VPS35 KD, and TRIM62 + VPS35 double KD A549 cells. Insets showing zoomed-in images of F-actin patches on WASH1 spots. Scale bars, 20 µm. Quantification (right) showing WASH1-associated F-actin intensity. $n \geq 90$ cells/siRNA ($N = 3$ independent experiments; mean ± SD; error bars represent SD; one-way ANOVA with Dunnett's multiple-comparisons test). *P* values for siControl vs siTRIM62: $P = 0.0004$; siControl vs siVPS35: $P = 0.0051$; siControl vs siTRIM62+siVPS35: $P < 0.0001$. Data in (**A–C**, **G**, **H**) are represented as box and whiskers plots. The center line represents the median, the box boundaries correspond to the 25th and 75th percentiles (interquartile range), the whiskers extend to the minimum and maximum values, and all individual data points are overlaid.

 © The Author(s)

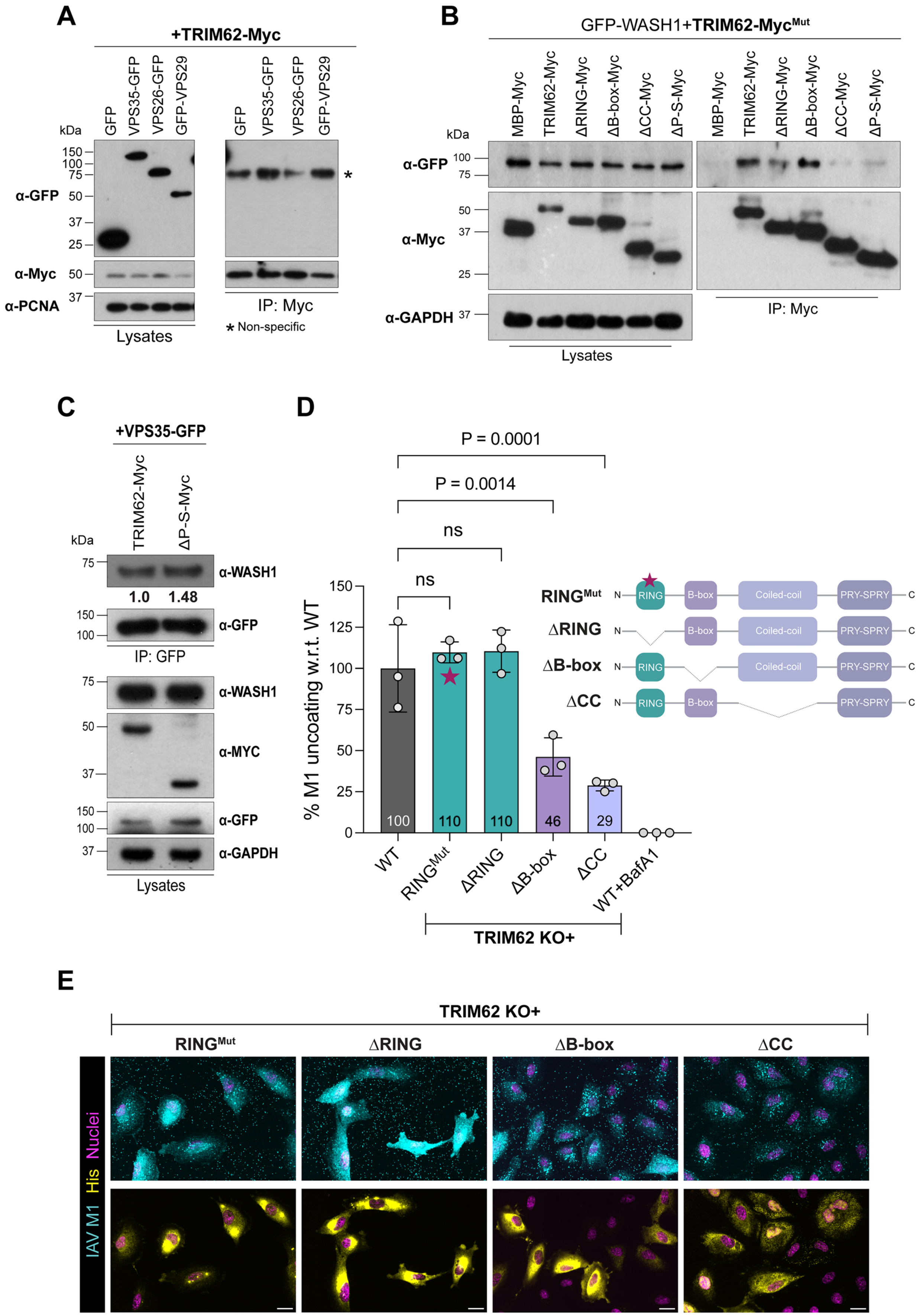

A
+TRIM62-Myc
GFP
VPS35-GFP
VPS26-GFP
GFP-VPS29
kDa
α-GFP
α-Myc
α-PCNA
Lysates
IP: Myc
* Non-specific
B
GFP-WASH1+TRIM62-Myc^Mut
MBP-Myc
TRIM62-Myc
ΔRING-Myc
ΔB-box-Myc
ΔCC-Myc
ΔP-S-Myc
α-GFP
α-Myc
α-GAPDH
Lysates
IP: Myc
C
+VPS35-GFP
TRIM62-Myc
ΔP-S-Myc
α-WASH1
1.0 1.48
α-GFP
IP: GFP
α-MYC
α-GAPDH
Lysates
D
P = 0.0001
P = 0.0014
ns
ns
% M1 uncoating w.r.t. WT
100
110
110
46
29
WT
RING^Mut
ΔRING
ΔB-box
ΔCC
WT+BafA1
TRIM62 KO+
RING^Mut
ΔRING
ΔB-box
ΔCC
RING
B-box
Coiled-coil
PRY-SPRY
E
TRIM62 KO+
RING^Mut
ΔRING
ΔB-box
ΔCC
IAV M1 His Nuclei

Figure EV5. TRIM62 interacts with WASH via its C-terminal region and promotes IAV entry independent of its E3 ubiquitin ligase activity.

(**A**) Pulldown assay examining interactions between TRIM62 and retromer subunits: VPS35, VPS26, and VPS29. HEK293T cells were transfected with TRIM62-Myc and GFP-tagged retromer subunits and lysates were incubated with Myc-Trap beads. Western blotting was performed to detect GFP-tagged retromer subunits in the precipitates. PCNA was used as a loading control, and asterisk (*) indicates non-specific bands. (**B**) Pulldown assay showing interactions between WASH1 and TRIM62 truncation mutants. HEK293T cells were co-transfected with GFP-WASH1 and TRIM62-Myc mutant constructs, and lysates were incubated with Myc-Trap beads. Western blotting was performed to detect GFP-WASH1 in the precipitates. (**C**) Pulldown assay showing interactions between VPS35-GFP and WASH1. HEK293T cells were co-transfected with VPS35-GFP and TRIM62-Myc or TRIM62 ΔP-S-Myc, and lysates were incubated with GFP-Trap beads. Western blotting was performed to detect endogenous WASH1 in the precipitates. (**D**) Quantification (left) of % M1 uncoating normalized to WT. $n \geq 450$ cells/sample ($N = 3$ independent experiments; mean ± SD; error bars represent SD; one-way ANOVA with Dunnett's multiple-comparisons test). P values for WT vs RING^{Mut}: $P = 0.8337$; WT vs ΔRING: $P = 0.7950$; WT vs ΔB-box: $P = 0.0014$; WT vs ΔCC: $P = 0.0001$. Schematic showing TRIM62 domain organization (right). (**E**) High-content confocal images for Fig. EV5D showing M1 uncoating in WT, TRIM62 KO, and KO cells stably expressing His-tagged TRIM62 mutants. M1 (cyan), His (yellow), and nuclei (magenta). Scale bars, 20 µm. All western blots are representative of three independent experiments.

 © The Author(s)
