# Supplementary material for: TRIM62 facilitates influenza A virus entry by regulating WASH-dependent endosomal trafficking
Source: EMBO Rep. 2026 Sep 19;27(20):5953–90. doi: 10.1038/s44319-026-00942-x (PMC13694701; doi:10.1038/s44319-026-00942-x)
Supplement: Supplementary file 4 [file 44319_2026_942_MOESM3_ESM.zip › Source data Fig. 1/1I/Figure 1I.pdf]

Figure 1I

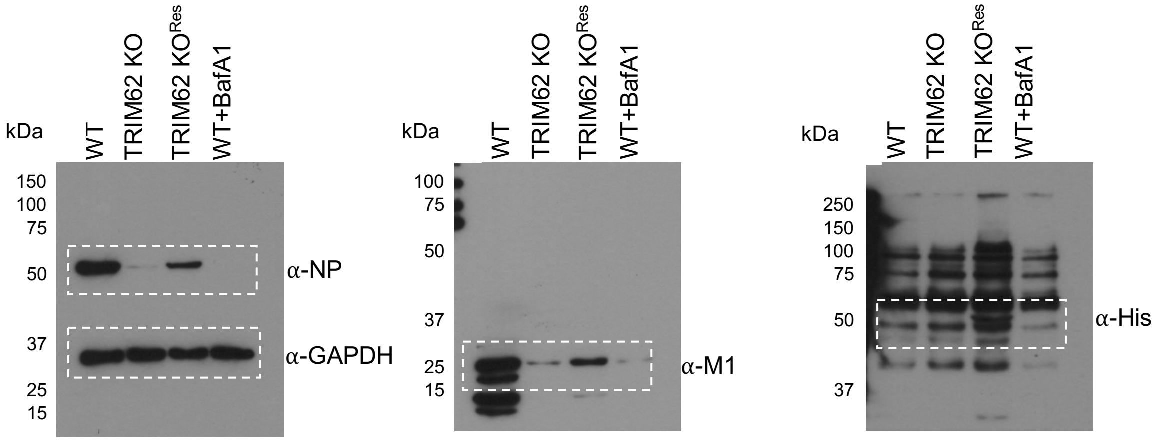
kDa
WT
TRIM62 KO
TRIM62 KO^{Res}
WT+BafA1
150
100
75
50
37
25
15
α-NP
α-GAPDH
kDa
WT
TRIM62 KO
TRIM62 KO^{Res}
WT+BafA1
100
75
50
37
25
15
α-M1
kDa
WT
TRIM62 KO
TRIM62 KO^{Res}
WT+BafA1
250
150
100
75
50
37
α-His
