# Supplementary material for: TRIM62 facilitates influenza A virus entry by regulating WASH-dependent endosomal trafficking
Source: EMBO Rep. 2026 Sep 19;27(20):5953–90. doi: 10.1038/s44319-026-00942-x (PMC13694701; doi:10.1038/s44319-026-00942-x)
Supplement: Supplementary file 8 [file 44319_2026_942_MOESM7_ESM.zip › Source data Fig. 5/5F/Figure 5F.pdf]

Fig 5F

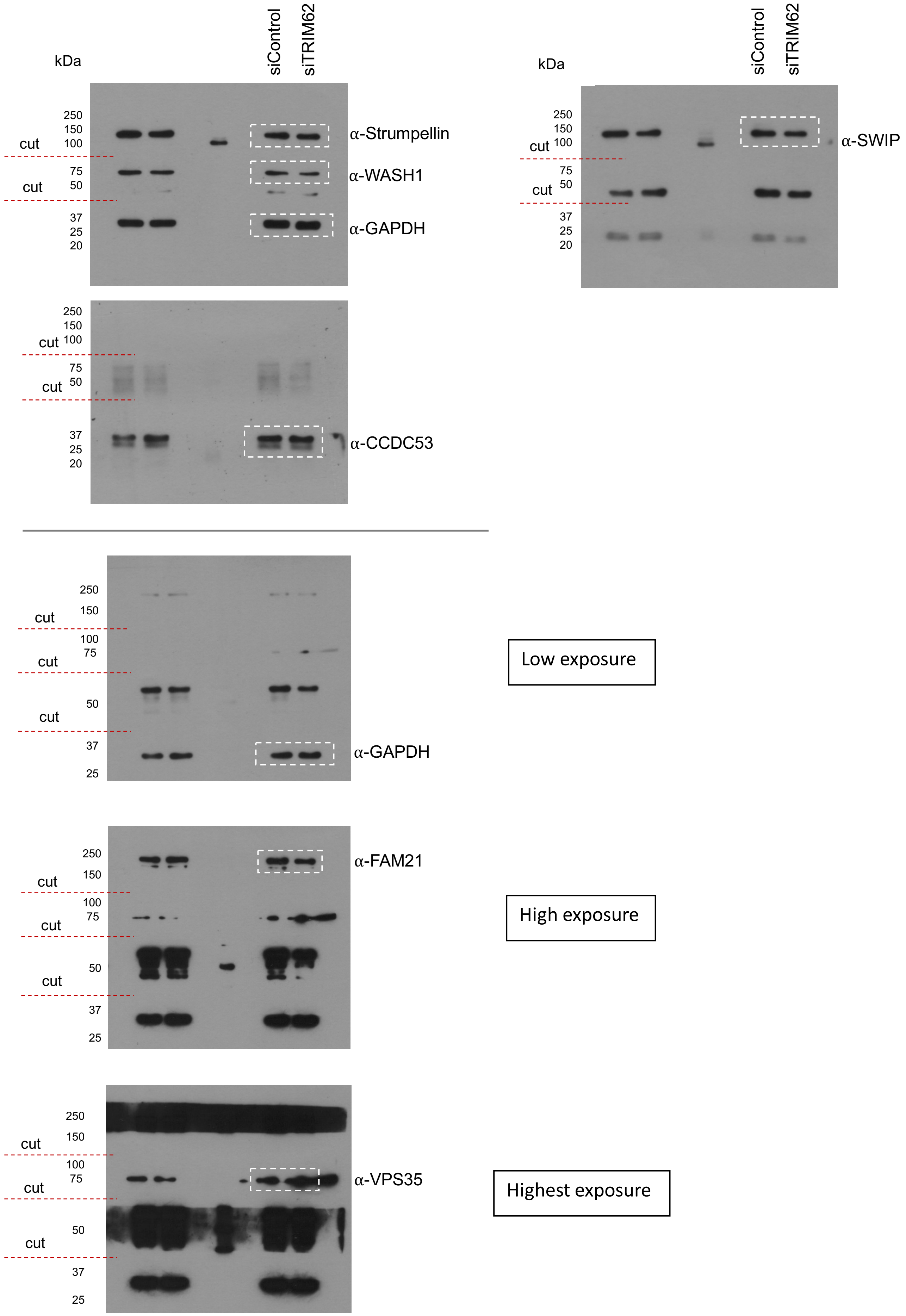

kDa
siControl
siTRIM62
250
150
100
75
50
37
25
20
cut
α-Strumpellin
α-WASH1
α-GAPDH
α-CCDC53
α-SWIP
Low exposure
High exposure
Highest exposure
α-FAM21
α-VPS35
