# Supplementary material for: TRIM62 facilitates influenza A virus entry by regulating WASH-dependent endosomal trafficking
Source: EMBO Rep. 2026 Sep 19;27(20):5953–90. doi: 10.1038/s44319-026-00942-x (PMC13694701; doi:10.1038/s44319-026-00942-x)
Supplement: Supplementary file 9 [file 44319_2026_942_MOESM8_ESM.zip › Source data Fig. 6/6A/Figure 6A.pdf]

Figure 6A

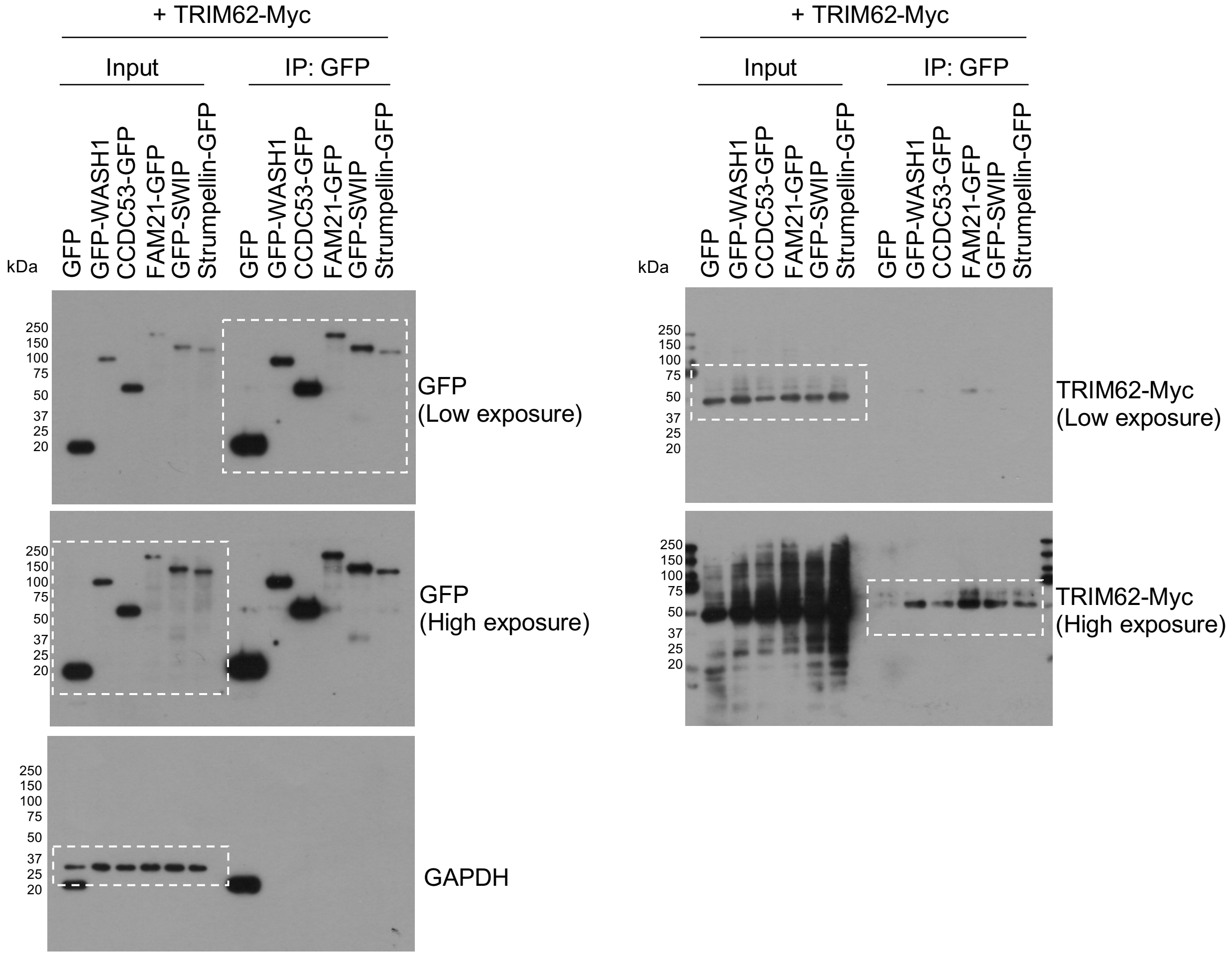
+ TRIM62-Myc
Input
IP: GFP
GFP
GFP-WASH1
CCDC53-GFP
FAM21-GFP
GFP-SWIP
Strumpellin-GFP
kDa
250
150
100
75
50
37
25
20
GFP
(Low exposure)
GFP
(High exposure)
GAPDH
TRIM62-Myc
(Low exposure)
TRIM62-Myc
(High exposure)
