# Supplementary material for: TRIM62 facilitates influenza A virus entry by regulating WASH-dependent endosomal trafficking
Source: EMBO Rep. 2026 Sep 19;27(20):5953–90. doi: 10.1038/s44319-026-00942-x (PMC13694701; doi:10.1038/s44319-026-00942-x)
Supplement: Supplementary file 9 [file 44319_2026_942_MOESM8_ESM.zip › Source data Fig. 6/6B/Figure 6B.pdf]

Figure 6B

-Trp, -Leu

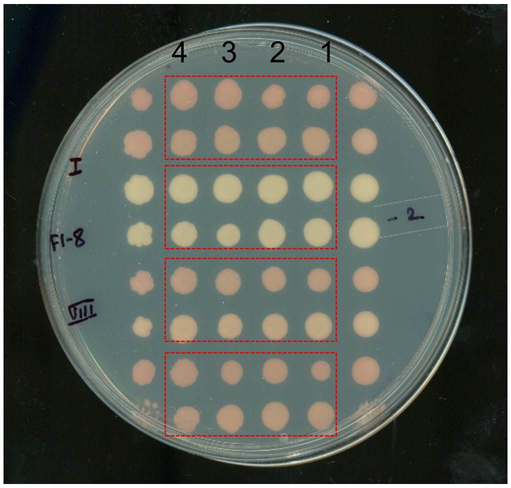

pGBK, pGAD
pGBK, TRIM62-pGAD
WASH1-pGBK, pGAD
WASH1-pGBK, TRIM62-pGAD
FAM21-pGBK, pGAD
FAM21-pGBK, TRIM62-pGAD
CCDC53-pGBK, pGAD
CCDC53-pGBK, TRIM62-pGAD

-Trp, -Leu, -His

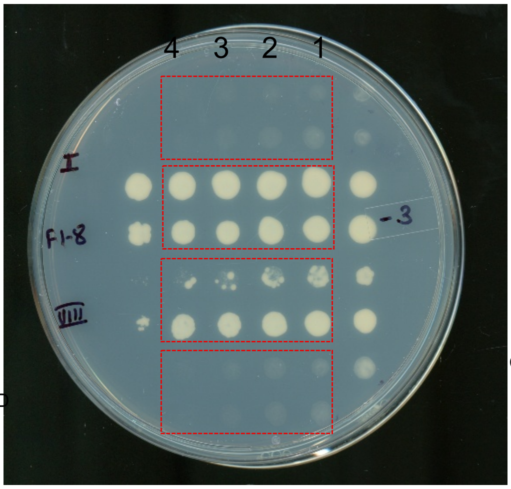

pGBK, pGAD
pGBK, TRIM62-pGAD
WASH1-pGBK, pGAD
WASH1-pGBK, TRIM62-pGAD
FAM21-pGBK, pGAD
FAM21-pGBK, TRIM62-pGAD
CCDC53-pGBK, pGAD
CCDC53-pGBK, TRIM62-pGAD

-Trp, -Leu

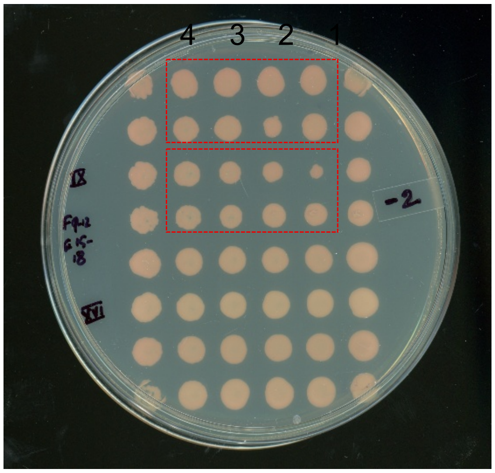

SWIP-pGBK, pGAD
SWIP-pGBK, TRIM62-pGAD
Strumpellin-pGBK, pGAD
Strumpellin-pGBK, TRIM62-pGAD

-Trp, -Leu, -His

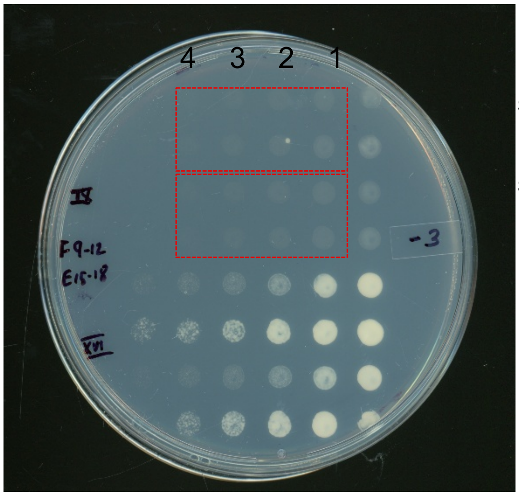

SWIP-pGBK, pGAD
SWIP-pGBK, TRIM62-pGAD
Strumpellin-pGBK, pGAD
Strumpellin-pGBK, TRIM62-pGAD
