# Supplementary material for: TRIM62 facilitates influenza A virus entry by regulating WASH-dependent endosomal trafficking
Source: EMBO Rep. 2026 Sep 19;27(20):5953–90. doi: 10.1038/s44319-026-00942-x (PMC13694701; doi:10.1038/s44319-026-00942-x)
Supplement: Supplementary file 9 [file 44319_2026_942_MOESM8_ESM.zip › Source data Fig. 6/6D/Figure 6D.pdf]

Figure 6D

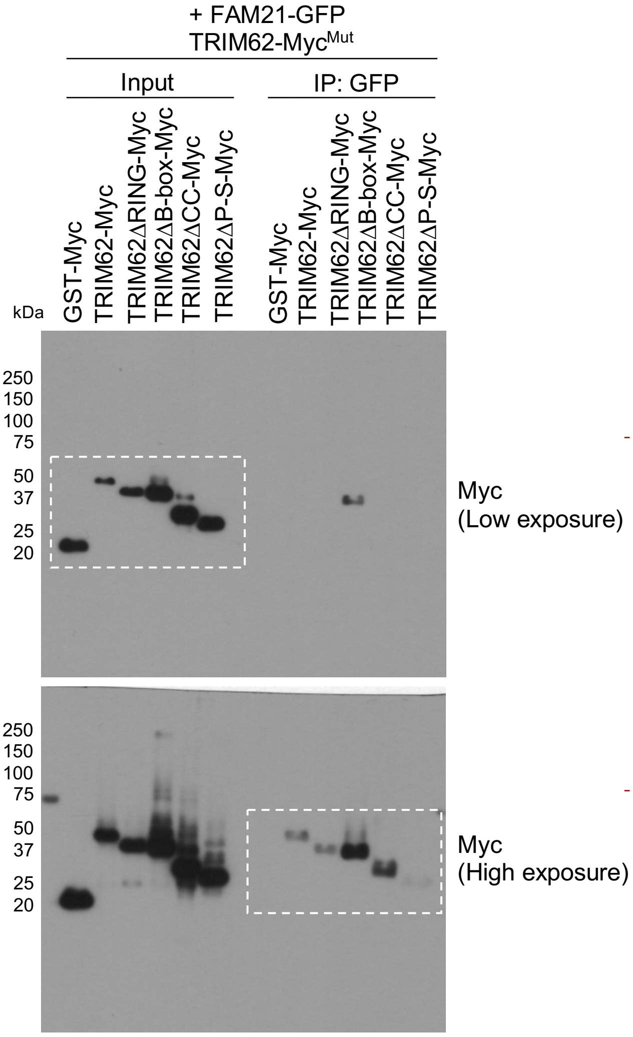
+ FAM21-GFP
TRIM62-Myc^Mut
Input
IP: GFP
GST-Myc
TRIM62-Myc
TRIM62ΔRING-Myc
TRIM62ΔB-box-Myc
TRIM62ΔCC-Myc
TRIM62ΔP-S-Myc
kDa
250
150
100
75
50
37
25
20
Myc
(Low exposure)
Myc
(High exposure)

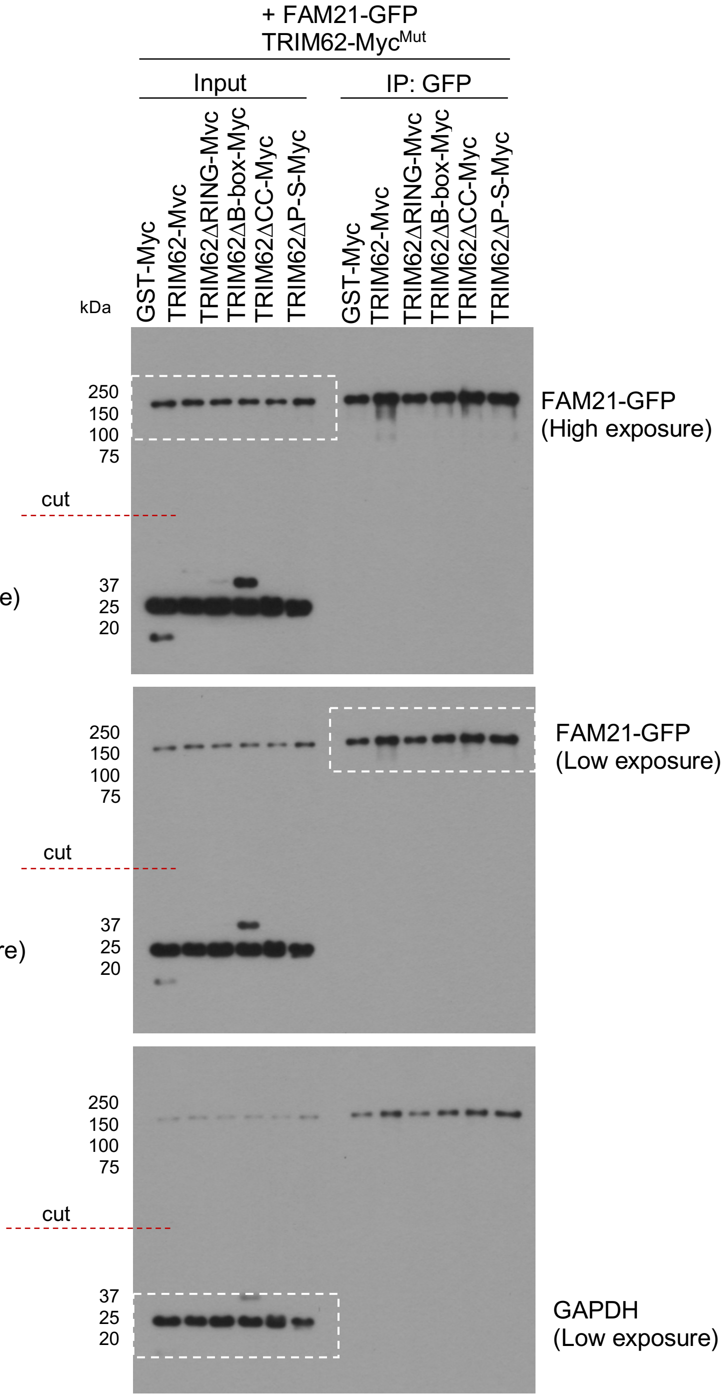
+ FAM21-GFP
TRIM62-Myc^Mut
Input
IP: GFP
GST-Myc
TRIM62-Myc
TRIM62ΔRING-Myc
TRIM62ΔB-box-Myc
TRIM62ΔCC-Myc
TRIM62ΔP-S-Myc
kDa
250
150
100
75
37
25
20
cut
FAM21-GFP
(High exposure)
FAM21-GFP
(Low exposure)
GAPDH
(Low exposure)
