# Supplementary material for: TRIM62 facilitates influenza A virus entry by regulating WASH-dependent endosomal trafficking
Source: EMBO Rep. 2026 Sep 19;27(20):5953–90. doi: 10.1038/s44319-026-00942-x (PMC13694701; doi:10.1038/s44319-026-00942-x)
Supplement: Supplementary file 9 [file 44319_2026_942_MOESM8_ESM.zip › Source data Fig. 6/6C/Figure 6C.pdf]

Figure 6C

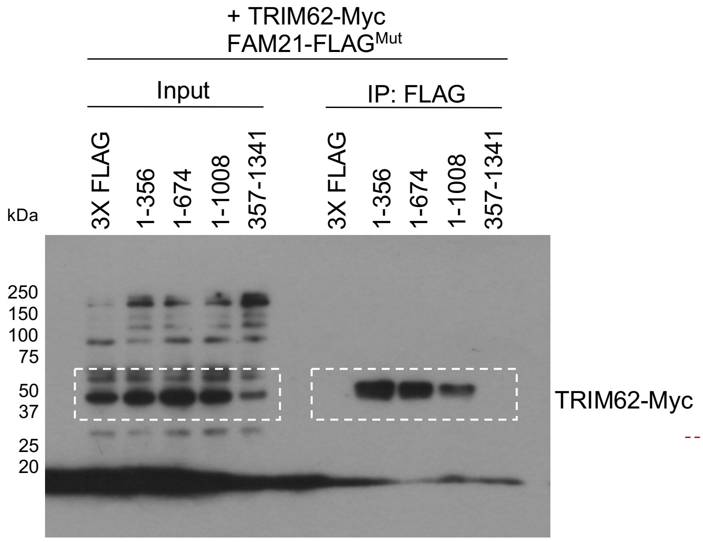
+ TRIM62-Myc
FAM21-FLAG^Mut
Input
IP: FLAG
3X FLAG
1-356
1-674
1-1008
357-1341
kDa
250
150
100
75
50
37
25
20
TRIM62-Myc

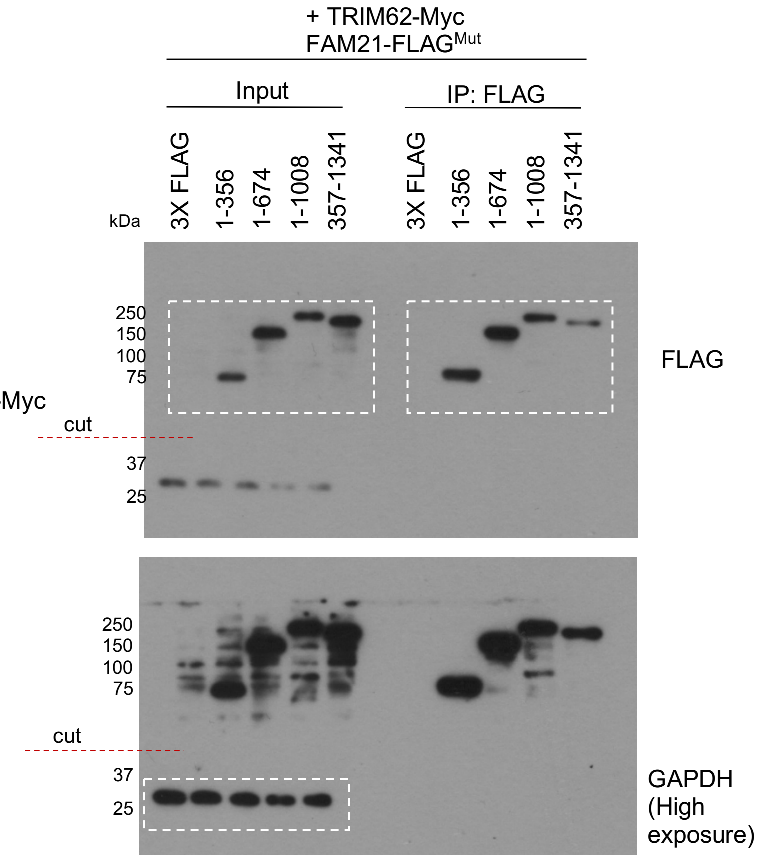
+ TRIM62-Myc
FAM21-FLAG^Mut
Input
IP: FLAG
3X FLAG
1-356
1-674
1-1008
357-1341
kDa
250
150
100
75
cut
37
25
FLAG
250
150
100
75
cut
37
25
GAPDH
(High
exposure)
